# Mechanistic Studies on Rhodium-Catalyzed Chemoselective Cycloaddition of Ene-Vinylidenecyclopropanes: Water-Assisted Proton Transfer

**DOI:** 10.3390/molecules29051085

**Published:** 2024-02-29

**Authors:** Ziqi Yu, Min Shi, Yin Wei

**Affiliations:** 1 Key Laboratory for Advanced Materials, Institute of Fine Chemicals, School of Chemistry and Molecular Engineering, East China University of Science and Technology, Meilong Road No. 130, Shanghai 200237, China; 18321678772@163.com; 2 State Key Laboratory of Organometallic Chemistry, Center for Excellence in Molecular Synthesis, Shanghai Institute of Organic Chemistry, Chinese Academy of Sciences, University of Chinese Academy of Sciences, 345 Lingling Road, Shanghai 200032, China

**Keywords:** mechanistic studies, rhodium-catalyzed cycloaddition, water-assisted proton transfer, temperature-controlled chemoselectivity

## Abstract

Rhodium-catalyzed cycloaddition reactions are a powerful tool for the construction of polycyclic compounds. Combined experimental and DFT studies were used to investigate the temperature-controlled chemoselectivity of cationic rhodium-catalyzed intramolecular cycloaddition reactions of ene-vinylidenecyclopropanes. After a series of mechanistic studies, it was found that trace amounts of water in the reaction system play an important role in generating the product with *endo* double bond located on a five-membered ring and revealed that trace amounts of water in the reaction system, including the rhodium catalyst, substrate and solvent, were sufficient to promote the formation of the product with *endo* double bond located on a five-membered ring, and additional water could not further accelerate the reaction. DFT calculation results show that the addition of water indeed significantly lowers the energy barrier of the proton transfer step, making the formation of the product with *endo* double bond located on a five-membered ring more likely to occur and confirming the rationality of water-assisted proton transfer occurring in the selective access to the product with *endo* double bond located on a five-membered ring.

## 1. Introduction

Transition-metal-catalyzed chemical transformations of vinylidenecyclopropanes (VDCPs), methylenecyclopropanes (MCPs), vinylcyclopropanes (VCPs) and other strained small ring compounds [1,2,3,4,5,6,7,8,9,10] with high tension have been frequently used in recent years to synthesize polycyclic skeletons, which have potential applications in the field of medicinal chemistry and materials [11,12,13,14,15]. Among them, rhodium catalysts play an important role in the field of catalytic cyclization. For example, in 2013, Cui’s group developed Rh(III)-catalyzed C–H activation/cycloaddition of benzamides and methylenecyclopropanes, constructing spiro dihydroisoquinolinones and furan-fused azepinones [16]. In 2017, Yu’s group developed a rhodium(I)-catalyzed intramolecular bridged [5 + 2] cycloaddition of cis-allene-VCPs to obtain bicyclo[4.3.1]decane skeleton [17]. In the same year, Evans’ group developed a rhodium-catalyzed ene-cycloisomerization of allylic-sulfide-containing alkenylidenecyclopropanes, which affords functionalized five-membered carbo- and heterocyclic rings with a transposed allylic sulfide [18]. In 2020, Wang’s group reported Rh(I)-catalyzed reaction of siloxyvinylcyclopropanes and diazoesters, which leads to the formation of siloxyvinylcyclobutane and 1,4-diene derivatives [19]. In these reactions, density functional theory (DFT) calculations were proved to be a powerful and effective tool for exploring the mechanistic insights of these rhodium-catalyzed reactions [20,21,22].

**Scheme 1:**
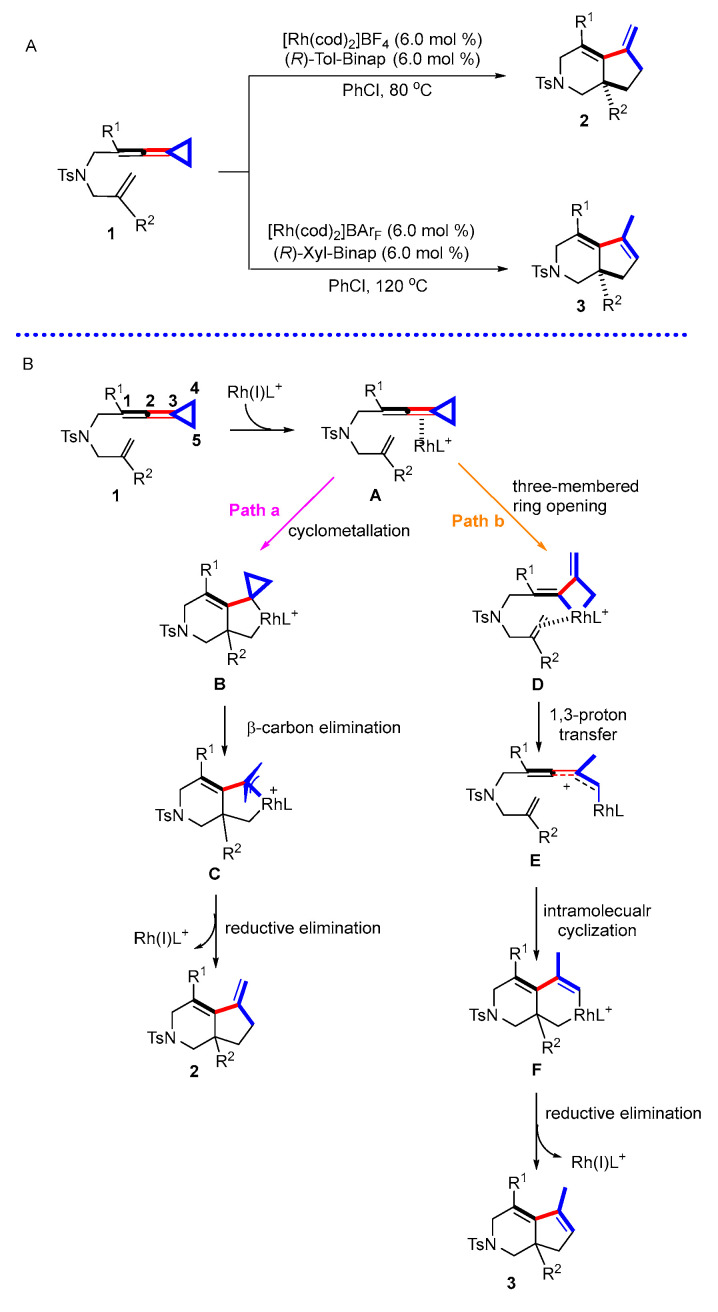
(**A**) Rh(I)-catalyzed stereoselective intramolecular cycloaddition reactions of ene-vinylidenecyclopropanes; (**B**) previously proposed reaction mechanism.

In 2019, our group reported Rh(I)-catalyzed stereoselective intramolecular cycloaddition reactions of ene-vinylidenecyclopropanes, constructing fused 6,5-bicyclic skeleton (Scheme 1A) [23]. Interestingly, when the substituent in the quaternary carbon center is a methyl group, the reaction shows exclusive temperature-controlled regioselectivity. When the temperature is 80 °C, a product with an *endo* double bond can be obtained, while when the temperature is 120 °C, a product with an *exo* double bond is obtained. Based on experimental results and previous studies, two possible reaction pathways were proposed in this paper (Scheme 1B). In pathway a, Rh first coordinates with the allene moiety C2-C3 double bond of VDCP to produce complex **A**, which undergoes cyclometallation to form a rhodacyclic intermediate **B**. The intermediate **B** undergoes β-carbon elimination to produce π-allyl rhodacyclic intermediate **C**, which then undergoes reductive elimination to render product **2**; in pathway b, complex **A** alternatively produces intermediate **D** through cleavage of the C4–C5 bond at the distal end of the three-membered ring, which undergoes 1,3-proton transfer to obtain intermediate **E** and, after successive cyclometallation, to produce intermediate **F** and then to generate product **3** through a reductive elimination process.

**Scheme 2:**
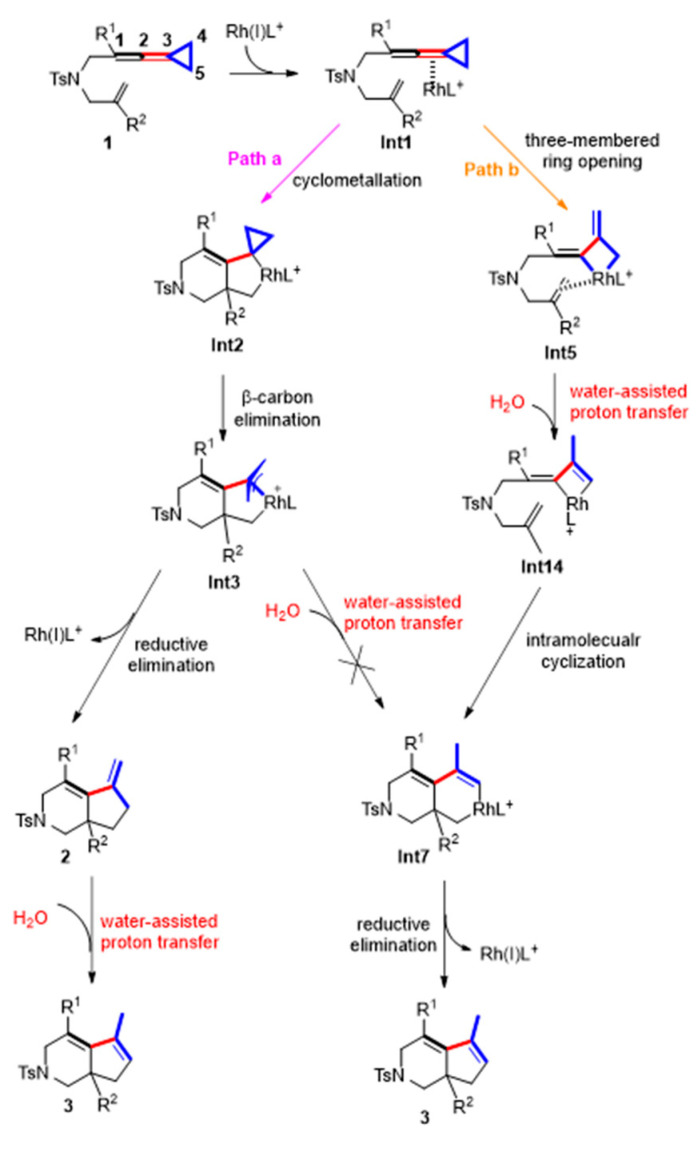
Currently proposed reaction mechanism.

Although we proposed a reaction mechanism in a previous report, the detailed mechanistic studies have not been conducted. Some important mechanistic issues still remain unanswered: (1) accessing different products from the same starting materials by fine-tuning the reaction conditions; (2) the critical factors to control product selectivity; (3) deep understanding of the reaction mechanisms. In this paper, we attempt to provide a thorough understanding of the reaction mechanism as well as the nature of product selectivity via a series of mechanistic studies, which is expected to enlighten the related rhodium-catalyzed reactions. In general, this direct 1,3-proton transfer is difficult due to the high energy barrier [24,25]; thus, we had to consider whether there are other factors to assist in the formation of product **3**. Previous reports [26,27,28,29,30] revealed that trace amounts of water in the reaction system could promote the reactions; therefore, we started to consider the influence of trace amounts of water in the reaction system on this reaction. Herein, we first perform DFT studies to explore whether this direct proton transfer process can proceed smoothly, and then, we explore the details of whether and how water plays a role in the reaction through DFT calculations in conjunction with the control experiments. We hypothesized that water-assisted proton transfer probably exists in the three processes, as shown in Scheme 2, to help generate product **3** at high temperature. DFT calculations were conducted to reveal that water-assisted proton transfer starting from **2** could take place to yield product **3**, or the intermediate **Int5** obtained through cleavage of the C4–C5 bond at the distal end of the three-membered ring undergoes water-assisted proton transfer to generate an intermediate **Int14**, which subsequently undergoes cyclometallation and reductive elimination, releasing rhodium catalysts to render product **3**. However, starting from π-allyl rhodacyclic intermediate **Int3**, the water in the reaction system does not play a role in reducing the reaction energy barrier to afford product **3**.

## 2. Results and Discussion

### 2.1. Mechanistic Studies through Experiments

**Scheme 3:**
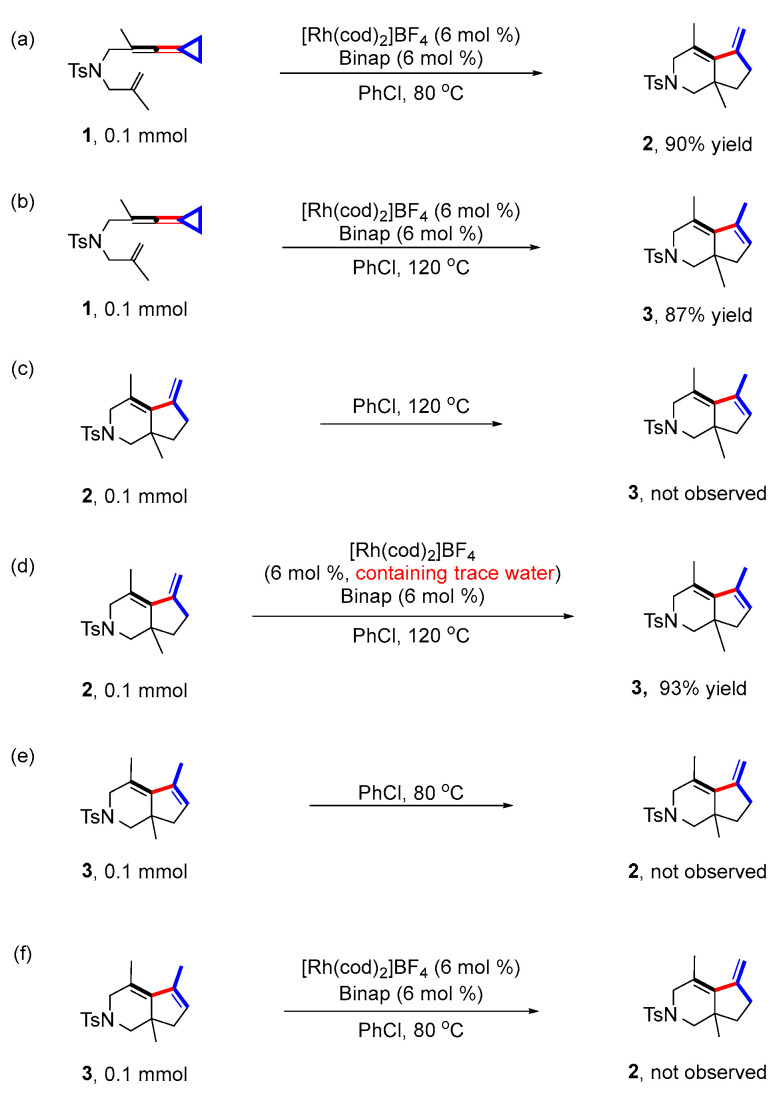
Control experiments. (**a**) The reaction was carried out at 80 °C with **1** as the substrate, PhCl as the solvent, [Rh(cod)_2_]BF_4_ as the catalyst and BINAP as the ligand; (**b**) The reaction was carried out at 120 °C with **2** as the substrate, PhCl as the solvent, [Rh(cod)_2_]BF_4_ as the catalyst and BINAP as the ligand; (**c**) The reaction was carried out at 120 °C with **2** as the substrate, PhCl as the solvent; (**d**) The reaction was carried out at 120 °C with **2** as the substrate, PhCl as the solvent, [Rh(cod)_2_]BF_4_ as the catalyst and BINAP as the ligand; (**e**) The reaction was carried out at 80 °C with **3** as the substrate, PhCl as the solvent; (**f**) The reaction was carried out at 80 °C with **3** as the substrate, PhCl as the solvent, [Rh(cod)_2_]BF_4_ as the catalyst and BINAP as the ligand.

First, we repeated the control experiments from our previous report [24] (Scheme 3) (for detailed information, see page S12 in the Supporting Information). The same results were obtained as shown in the previous report. Under the standard reaction conditions, the reaction of substrate **1** took place smoothly, affording product **2** with 90% yield at 80 °C and product **3** with 87% yield at 120 °C, respectively (Scheme 3a,b); the exocyclic double bond product **2** could not be directly converted to *endo* double bond product **3** at 120 °C without the rhodium catalyst and ligand (Scheme 3c). Surprisingly, product **2** was converted to **3** with 93% yield at 120 °C with [Rh(cod)_2_]BF_4_ as the catalyst and Binap as the ligand, which was different to previous findings [24] (Scheme 3d). We speculated that the system may already contain trace amounts of water to assist in the formation of product **3**. In addition, during the weighing of the rhodium catalyst, we found that the original red brown powder was very susceptible to moisture. Therefore, we measured the water content of our used rhodium catalyst via Karl Fischer coulometric titrations [31,32,33,34] and unsurprisingly found that the rhodium catalyst had a water content of 3.59% (for detailed information, see page S18 in the Supporting Information). Furthermore, the control experiments showed that **3** could not be converted to **2** at both 80 °C and under standard conditions (Scheme 3e,f).

**Scheme 4:**
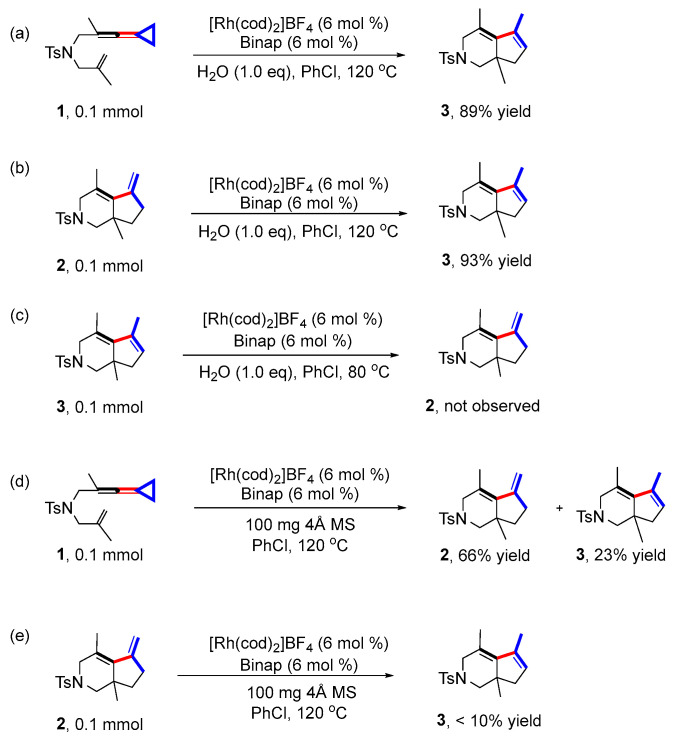
Control experiments involving water. (**a**) The reaction was carried out at 120 °C with **1** as the substrate, PhCl as the solvent, [Rh(cod)_2_]BF_4_ as the catalyst, BINAP as the ligand and H_2_O as additive; (**b**) The reaction was carried out at 120 °C with **2** as the substrate, PhCl as the solvent, [Rh(cod)_2_]BF_4_ as the catalyst, BINAP as the ligand and H_2_O as additive; (**c**) The reaction was carried out at 80 °C with **3** as the substrate, PhCl as the solvent [Rh(cod)_2_]BF_4_ as the catalyst, BINAP as the ligand and H_2_O as additive; (**d**) The reaction was carried out at 120 °C with **1** as the substrate, PhCl as the solvent, [Rh(cod)_2_]BF_4_ as the catalyst, BINAP as the ligand and 4Å molecular sieves; (**e**) The reaction was carried out at 120 °C with **2** as the substrate, PhCl as the solvent, [Rh(cod)_2_]BF_4_ as the catalyst, BINAP as the ligand and 4Å molecular sieves.

Subsequently, we conducted some control experiments through addition of water in the reaction system to investigate the role of water in the reaction (Scheme 4) (for detailed information, see page S14 in the Supporting Information). Employing **1** or **2** as the starting material, the reactions were conducted at 120 °C or under standard conditions with the addition of water (1.0 eq); product **3** was obtained with 89% and 93% yields, respectively, suggesting that additional water had a slight effect on the yields of **3** (Scheme 4a,b). Moreover, **3** was not converted to **2** with the addition of water (1.0 eq) under standard conditions at 80 °C, indicating that **3** cannot be converted to **2** with the assistance of water either (Scheme 4c). Then, when 4Å molecular sieves (100 mg) were added to get rid of trace amounts of water in the reaction system (Scheme 4d), **3** was obtained with 23% yield and **2** with 66% yield, suggesting that the water in the reaction system did not have a significant influence on the formation of product **2** but may have inhibited the formation of **3**. Subsequently, when 4Å molecular sieves (100 mg) were also added to the reaction system of **2**–**3** (Scheme 4e), only less than 10% of **3** was obtained. It was clear that the conversion of **2** to **3** was inhibited, indicating that trace amounts of water are likely to play a critical role in affording product **3**.

**Scheme 5:**
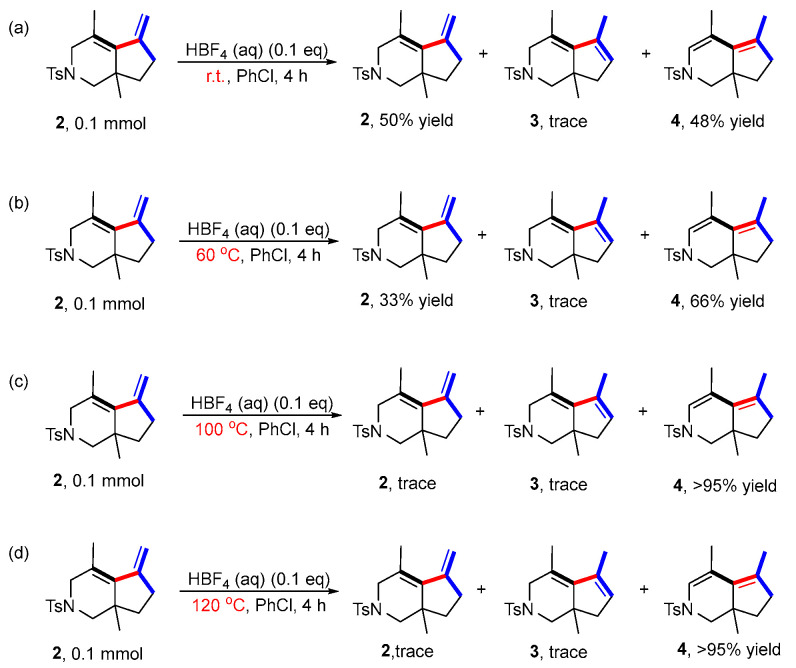
Control experiments involving HBF_4_. (**a**) The reaction was carried out at room temperature with **2** as the substrate, PhCl as the solvent, [Rh(cod)_2_]BF_4_ as the catalyst, BINAP as the ligand and HBF_4_ as additive; (**b**) The reaction was carried out at 60 °C with **2** as the substrate, PhCl as the solvent, [Rh(cod)_2_]BF_4_ as the catalyst, BINAP as the ligand and HBF_4_ as additive; (**c**) The reaction was carried out at 100 °C with **2** as the substrate, PhCl as the solvent, [Rh(cod)_2_]BF_4_ as the catalyst, BINAP as the ligand and HBF_4_ as additive; (**d**) The reaction was carried out at 120 °C with **2** as the substrate, PhCl as the solvent, [Rh(cod)_2_]BF_4_ as the catalyst, BINAP as the ligand and HBF_4_ as additive.

The presence of BF_4_^−^ anion in the system in situ may produce the strong Brϕnsted acid HBF_4_, which may promote the conversion of product **2** to product **3** under acid catalysis. Therefore, other control experiments were performed to investigate this hypothesis. An aqueous solution of HBF_4_ was added to the reaction in absence of the rhodium catalyst and ligand to investigate whether product **2** was converted to **3** at different reaction temperatures (Scheme 5) (for details, see page S15 in the Supporting Information). The results showed that only trace amounts of product **3** at room temperature, 60 °C, 100 °C and 120 °C were obtained. Moreover, a new compound **4**, isomerized from the two double bonds of product **2**, was obtained with increasing yield by increasing the reaction temperature, indicating that the conversion of **2** to **3** did not undergo the mechanism of acid catalysis.

**Figure 1:**
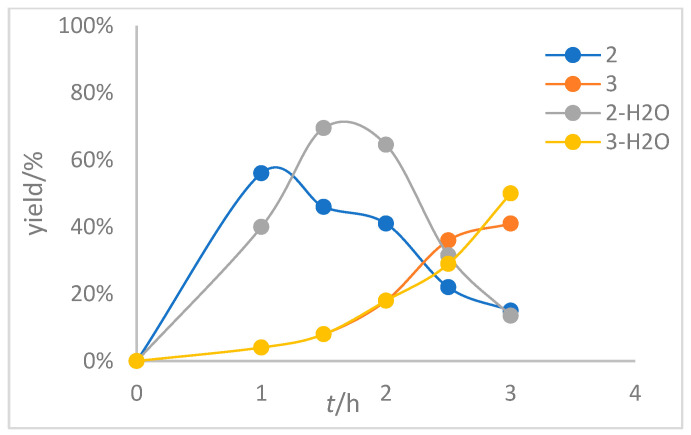
Variation curves of 2 and 3 with and without additional water (1.0 eq) using **1** as substrate. The reaction was carried out at 120 °C with **1** as the substrate, a mixture of toluene:DMF = 19:1 as the solvent, [Rh(cod)_2_]BF_4_ as the catalyst and BINAP as the ligand.

To further investigate the role of water in this reaction, we first added additional water (1.0 eq) to the reaction system, employing **1** as the substrate under standard conditions, and monitored the reaction course along with those systems without additional water for comparison; the experimental results are shown in Figure 1 (for detailed information, see Table S1 on page S17 and Table S2 on page S18 in the Supporting Information ). In Figure 1, we can find that, first, in the process of the formation of product **3**, the yield of product **2** increased first and then decreased gradually, indicating that product **3** may be converted from product **2**, which is also in line with the result shown in Scheme 4d; however, we cannot completely exclude the possibility of direct conversion from substrate **1** to **3**. Second, whether or not additional water is added has almost no effect on the rate of formation of product **3**. This indicates that trace amounts of water are already sufficient to promote the formation of product **3**, and more water in the reaction system cannot further accelerate the reaction. In terms of the difference in the rate of product **2** formation during the reaction, we attribute this to experimental errors.

**Figure 2:**
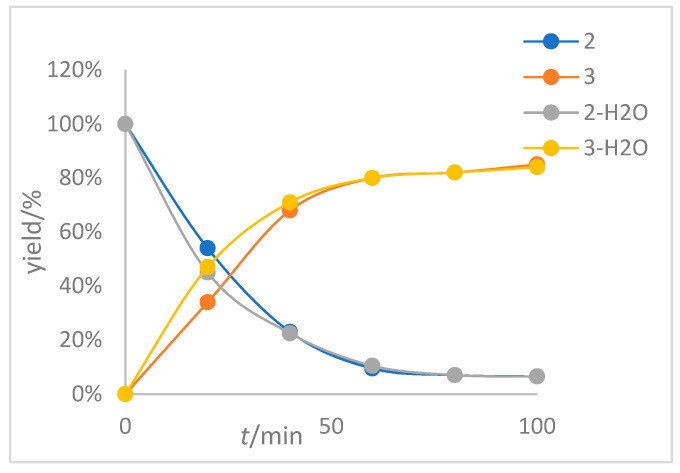
Variation curves of 2 and 3 with and without additional water (1.0 eq) with **2** as substrate. The reaction was carried out at 120 °C with **2** as the substrate, PhCl as the solvent, [Rh(cod)_2_]BF_4_ as the catalyst and BINAP as the ligand.

Subsequently, we added additional water to the reaction system utilizing **2** as the substrate under standard conditions at 120 °C and monitored the reaction course along with those systems without additional water (1.0 eq) for comparison; the experimental results are shown in Figure 2 (for detailed information, see Table S3 on page S19 and Table S4 on page S20 in the Supporting Information). It is clear that adding additional water or not has almost no effect on the rate of the reaction. As mentioned before, the rhodium catalyst had a water content of 3.59% (for detailed information, see page S18 in the Supporting Information). Subsequently, we similarly determined the water content of substrate **1** and found it contained 0.8% of moisture (for detailed information, see page S21 in the Supporting Information), and the solvent super dry chlorobenzene we used also contained a trace amount of water (≤50 ppm). These results indicate that the reaction system inevitably contains trace amounts of water, and the reaction outcomes are mainly influenced by the rhodium catalyst. This is in agreement with our speculation.

### 2.2. Two Proposed Reaction Pathways Investigated through DFT Calculations

**Scheme 6:**
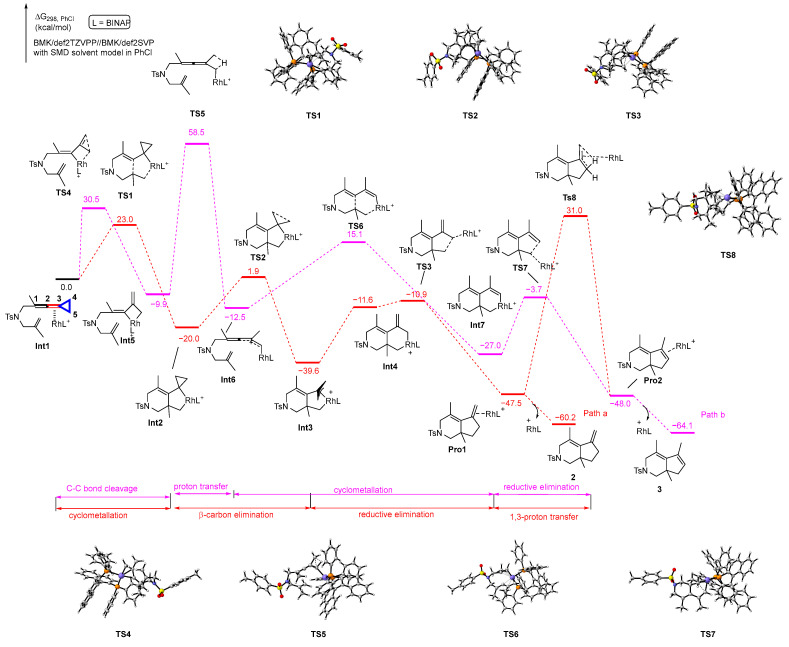
Solvation Gibbs free energy profiles for the reaction of two proposed pathways. Purple circle is Rh atom, blue circle is N atom, yellow circle is S atom, red circle is O atom, orange circle is P atom.

We first performed DFT calculations to investigate the reaction profiles of the proposed mechanisms for the generation of products **2** and **3** (Scheme 6). Initially, the reactant complex **Int1**, in which the rhodium catalyst coordinates with the allene moiety C2–C3 double bond of VDCP, undergoes cyclometallation to generate an intermediate **Int2** via transition state **TS1** with 23.0 kcal/mol energy barrier, which is exergonic at 20.0 kcal/mol; subsequently, the intermediate **Int2** undergoes β-carbon elimination, resulting in three-membered ring opening to render a π-allyl rhodacyclic intermediate **Int3** via transition state **TS2** with an energy barrier of 21.9 kcal/mol, which is exergonic at 19.6 kcal/mol. Then, the product complex **Pro1** is generated via reductive elimination from **Int3** via transition state **TS3** with 28.7 kcal/mol energy barrier, which is the highest barrier in the whole reaction pathway. The product complex **Pro1** can release the rhodium catalyst and ligand to generate the corresponding product **2**. During this course, an intermediate **Int4** is also obtained via isomerization of **Int3**; however, the energy of **Int4** is higher than that of **Int3** by 28.0 kcal/mol. Based on the energy profile along Path a, we found that this pathway is reasonable for proceeding to render product **2**. Subsequently, we also explored the 1,3-proton transfer process from **Pro1** to **Pro2** and found that the process had an energy barrier of 78.5 kcal/mol via transition state **TS8**, which indicated that it is impossible for the process to occur at 120 °C, meaning that product **2** cannot be converted to product **3** through direct 1,3-proton transfer. Alternatively, the reactant complex **Int1** undergoes C4-C5 bond cleavage to form the rhoda-cyclobutane intermediate **Int5**, passing through transition state **TS4** with 30.5 kcal/mol energy barrier. Subsequently, the 1,3-proton transfer occurs at the intermediate **Int5**, resulting in the formation of intermediate **Int6**, which passes through transition state **TS5** with 68.4 kcal/mol energy barrier. Afterward, the intermediate **Int6** undergoes cyclometallation to afford intermediate **Int7** via transition state **TS6** with 27.6 kcal/mol energy barrier. Passing through transition state **TS7** with 23.3 kcal/mol energy barrier, the intermediate **Int7** goes through reductive elimination to generate **Pro2**, which finally releases the rhodium and ligand to afford the corresponding product **3**. According to the calculation results for Path b, not surprisingly, we can observe that the 1,3-proton transfer process has such a high energy barrier of 68.4 kcal/mol that it cannot take place at that temperature. Thus, product **3** cannot be directly obtained through Path b.

### 2.3. Theoretical Investigation of the Reaction Pathways Involving Water-Assisted Proton Transfer

**Scheme 7:**
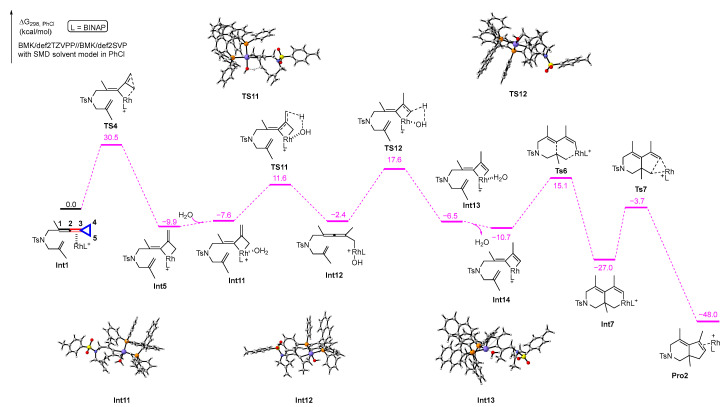
Solvation Gibbs free energy profiles for the process of water-assisted proton transfer in Path b. Purple circle is Rh atom, blue circle is N atom, yellow circle is S atom, red circle is O atom, orange circle is P atom.

We can conclude from the above calculations that product **2** is accessible, but product **3** is difficult to obtain whether the proton transfer occurs in the step from **Int5** to **Int6** or the step from **Pro1** to **Pro2**. Combined with the previous experimental results, we began to consider that the presence of trace water in the reaction system might have contributed to the formation of product **3**. Subsequently, we found in our experiments that the rhodium catalyst we used readily absorbed water, and together with some previous studies [23], we began to explore whether water could assist in the proton transfer process of this reaction and the details of the role of water with DFT calculations. We first performed DFT calculations to investigate the water-assisted proton transfer process in Path b. The results of the calculations are shown in Scheme 7. In this process, water is involved in the reaction starting from **Int5**. First, a molecule of water is coordinated with the rhodium atom of **Int5** to produce the intermediate **Int11**, which is a slightly endothermic process with 2.3 kcal/mol. Subsequently, a proton of water is transferred to the terminal carbon atom C4 of the *exo* double bond of the four-membered rhodium ring to generate the hydroxyl-rhodium intermediate **Int12**, passing through transition state **TS11** with an activation barrier of 19.2 kcal/mol; afterward, a hydrogen atom at the carbon atom C5 is abstracted by the hydroxyl group to obtain the double-bonded four-membered ring tetrameric rhodium intermediate **Int13**, which is required to overwhelm an energy barrier with 20.0 kcal/mol via transition state **TS12**. The intermediate **Int14** is generated when the water molecule on rhodium is released, which gives off 4.2 kcal/mol of energy; then, **Int14** undergoes cyclometallation to obtain the intermediate **Int7**, which passes through transition state **TS6** with an exothermic heat of 25.8 kcal/mol. Finally, **Pro2** is produced by the reductive elimination of **Int7** via transition state **TS7** with 23.3 kcal/mol energy barrier.

**Scheme 8:**
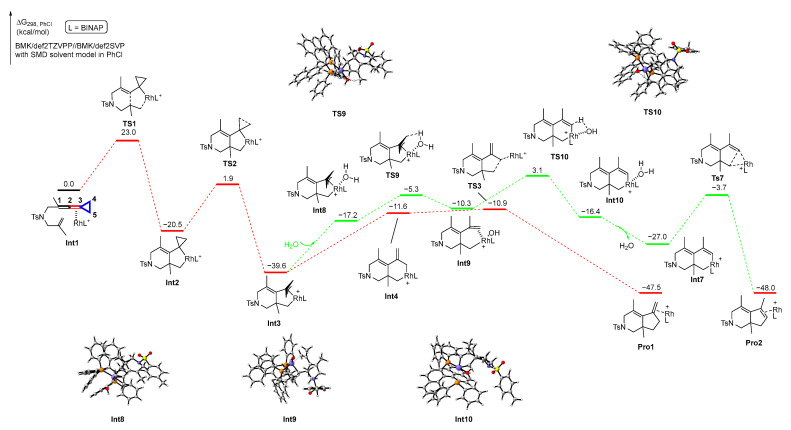
Solvation Gibbs free energy profiles for the process of water-assisted proton transfer in Path a. Purple circle is Rh atom, blue circle is N atom, yellow circle is S atom, red circle is O atom, orange circle is P atom.

Subsequently, we further investigated whether the formation of product **3** can be promoted in the presence of water starting from π-allyl rhodacyclic intermediate **Int3**. Thus, we continued to investigate the possible process with DFT calculations, and the results are shown in Scheme 8. Similar to the process in Path b, water is first coordinated with the rhodium atom of **Int3**, yielding intermediate **Int8**, which is an endothermic process with 22.4 kcal/mol. Subsequently, a proton on the water is transferred to the allyl group to produce the hydroxy-rhodium intermediate **Int9** through transition state **TS9**. Then, a proton on the carbon atom C5 is plucked off by the hydroxyl group through a four-membered ring transition state **TS10** with 13.4 kcal/mol energy barrier, resulting in **Int10**, which releases a molecule of water to generate **Int7**. Finally, **Pro2** is generated when reductive elimination occurs on **Int7** through transition state **TS7** with the energy barrier of 23.3 kcal/mol. However, with reference to **Int3**, the high energy barrier (42.7 kcal/mol) has to be overwhelmed in order to pass through the transition state **TS10**, which is difficult to overcome even when the reaction is conducted at 120 °C. Thus, we may exclude the possibility that the formation of product **3** can be promoted in the presence of water starting from π-allyl rhodacyclic intermediate **Int3**.

**Scheme 9:**
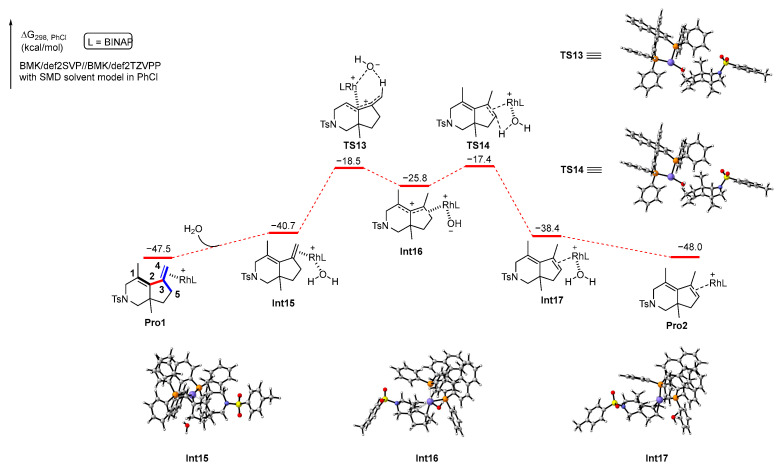
Solvation Gibbs free energy profiles for the reaction of **2** to produce **3**. Purple circle is Rh atom, blue circle is N atom, yellow circle is S atom, red circle is O atom, orange circle is P atom.

In addition, **Pro1**, which is the complex of product **2** and the rhodium catalyst, may also undergo proton transfer in the presence of water to produce **Pro2**, which leads to product **3**. We also performed DFT calculations to explore this process. The calculation results are shown in Scheme 9. First, a molecule of water is coordinated with the rhodium atom in **Pro1** to generate an intermediate **Int14**, which is an endothermic process with 6.8 kcal/mol of energy. Subsequently, a proton from the water passes through a six-membered ring transition state **TS13** with 22.2 kcal/mol energy barrier to the carbon atom C4 of the extra-ring double bond to obtain the intermediate **Int15**. Then, the hydrogen atom in the carbon atom C5 is plucked off by the hydroxyl group on the rhodium via transition state **TS14** with 8.4 kcal/mol energy barrier, resulting in **Int16**, which releases a molecule of water to produce **Pro2**. From an energetic point of view, the reaction energy barrier for proton transfer assisted by water is significantly lower compared to direct proton transfer. The calculation results agree with the result of the control experiment shown in Scheme 4b.

## 3. Materials and Methods

### 3.1. General Information

^1^H and ^13^C NMR spectra were recorded at 400 MHz and 600 MHz. Catalysts [Rh(COD)_2_]BF_4_ and ligands BINAP were purchased from Pepper Reagent. Toluene was distilled from sodium (Na) under argon (Ar) atmosphere. Super dry PhCl was purchased from Meryer, containing ≤50 ppm of water, and super dry DMF and HBF_4_(aq) were purchased from General-reagent^®^. Commercially obtained reagents were used without further purification. All reactions were monitored using TLC with silica gel coated plates (Huanghai GF254). Flash column chromatography was performed by using 300–400-mesh silica gel elution with ethyl acetate and petroleum ether at increased pressure. The content of water was determined by Metrohm 831KF.

### 3.2. Experimental Procedures for Control Experiments

#### 3.2.1. Experimental Procedures for **1** to **2** with Standard Conditions

A 10 mL dried tube was charged with **1** (0.1 mmol, 1.0 equiv), [Rh(COD)_2_]BF_4_ (6.0 mol %) and Binap (6.0 mol %). The reaction tube was evacuated and backfilled with argon (repeated three times). Then, PhCl (2.0 mL) was added into the tube. The reaction mixture was stirred at 80 °C for 12 h. The solvent was removed under reduced pressure, and the residue was purified using flash column chromatography (SiO_2_) to generate the corresponding product **2** (28.5 mg, 90%).

#### 3.2.2. Experimental Procedures for **1** to **3** with Standard Conditions

A 10 mL dried tube was charged with 1 (0.1 mmol, 1.0 equiv), [Rh(COD)_2_]BF_4_ (6.0 mol %) and Binap (6.0 mol %). The reaction tube was evacuated and backfilled with argon (repeated three times). Then, PhCl (2.0 mL) was added into the tube. The reaction mixture was stirred at 120 °C for 4 h. The solvent was removed under reduced pressure, and the residue was purified using flash column chromatography (SiO2) to generate the corresponding product **3** (27.6 mg, 87%).

#### 3.2.3. Experimental Procedures for **2** to **3** without Catalysts and Ligands

A 10 mL dried tube was charged with 2 (0.1 mmol, 1.0 equiv). The reaction tube was evacuated and backfilled with argon (repeated three times). Then, PhCl (2.0 mL) was added into the tube. The reaction mixture was stirred at 120 °C for 4 h. The solvent was removed under reduced pressure, and the residue was purified using flash column chromatography (SiO_2_). The product was confirmed with ^1^H NMR spectroscopic data. No product **3** was observed.

#### 3.2.4. Experimental Procedures for **2** to **3** with Standard Conditions

A 10 mL dried tube was charged with **2** (0.1 mmol, 1.0 equiv), [Rh(COD)_2_]BF_4_ (6.0 mol %) and Binap (6.0 mol %). The reaction tube was evacuated and backfilled with argon (repeated three times). Then, PhCl (2.0 mL) was added into the tube. The reaction mixture was stirred at 120 °C for 4 h. The solvent was removed under reduced pressure, and the residue was purified using flash column chromatography (SiO_2_) to generate the corresponding product **3** (29.4 mg, 93%). This indicates that product **2** can be converted to product **3** at 120 °C only under standard conditions.

#### 3.2.5. Experimental Procedures for **3** to **2** without Catalysts and Ligands

A 10 mL dried tube was charged with **3** (0.1 mmol, 1.0 equiv). The reaction tube was evacuated and backfilled with argon (repeated three times). Then, PhCl (2.0 mL) was added into the tube. The reaction mixture was stirred at 80 °C for 12 h. The solvent was removed under reduced pressure, and the residue was purified using flash column chromatography (SiO_2_). The product was analyzed with ^1^H NMR spectroscopic data. No product **2** was observed.

#### 3.2.6. Experimental Procedures for **3** to **2** with Standard Conditions

A 10 mL dried tube was charged with **3** (0.1 mmol, 1.0 equiv), [Rh(COD)_2_]BF_4_ (6.0 mol %) and Binap (6.0 mol %). The reaction tube was evacuated and backfilled with argon (repeated three times). Then, PhCl (2.0 mL) was added into the tube. The reaction mixture was stirred at 80 °C for 12 h. The solvent was removed under reduced pressure, and the residue was purified using flash column chromatography (SiO_2_). The product was confirmed with ^1^H NMR spectroscopic data. No product **2** was observed. It is suggested that product **3** cannot be converted to product **2** at 80 °C, whether or not standard conditions are applied.

### 3.3. Experimental Procedures for Control Experiments Involving Water

#### 3.3.1. Experimental Procedures for **1** to **3** with Standard Conditions and Water

A 10 mL dried tube was charged with **1** (0.1 mmol, 1.0 equiv), [Rh(COD)_2_]BF_4_ (6.0 mol %), Binap (6.0 mol %) and H_2_O (1.0 equiv). The reaction tube was evacuated and backfilled with argon (repeated three times). Then, PhCl (2.0 mL) was added into the tube. The reaction mixture was stirred at 120 °C for 4 h. The solvent was removed under reduced pressure, and the residue was purified using flash column chromatography (SiO_2_) to generate the corresponding product **3** (28.2 mg, 89%). There is no obvious difference between the two conditions of adding water and not adding water.

#### 3.3.2. Experimental Procedures for **2** to **3** with Standard Conditions and Water

A 10 mL dried tube was charged with **2** (0.1 mmol, 1.0 equiv), [Rh(COD)_2_]BF_4_ (6.0 mol %), Binap (6.0 mol %) and H_2_O (1.0 equiv). The reaction tube was evacuated and backfilled with argon (repeated three times). Then, PhCl (2.0 mL) was added into the tube. The reaction mixture was stirred at 120 °C for 4 h. The solvent was removed under reduced pressure, and the residue was purified using flash column chromatography (SiO_2_) to generate the corresponding product **3** (29.4 mg, 93%). This indicates that the addition of extra water did not have a significant effect on the conversion of **2** to **3**.

#### 3.3.3. Experimental Procedures for **3** to **2** with Standard Conditions and Water

A 10 mL dried tube was charged with **3** (0.1 mmol, 1.0 equiv), [Rh(COD)_2_]BF_4_ (6.0 mol %), Binap (6.0 mol %) and H_2_O (1.0 equiv). The reaction tube was evacuated and backfilled with argon (repeated three times). Then, PhCl (2.0 mL) was added into the tube. The reaction mixture was stirred at 120 °C for 4 h. The solvent was removed under reduced pressure, and the residue was purified using flash column chromatography (SiO_2_). The product was analyzed with ^1^H NMR spectroscopic data. No product **2** was observed.

#### 3.3.4. Experimental Procedures for **1** to **3** with Standard Conditions and 4Å Molecular Sieves

A 10 mL dried tube was charged with **1** (0.1 mmol, 1.0 equiv), [Rh(COD)_2_]BF_4_ (6.0 mol %), Binap (6.0 mol %) and 100 mg 4Å molecular sieves. The reaction tube was evacuated and backfilled with argon (repeated three times). Then, PhCl (2.0 mL) was added into the tube. The reaction mixture was stirred at 120 °C for 4 h. After the reaction was completed, the reaction mixture was cooled to ambient temperature. Then, the organic solvent was removed under reduced pressure to afford the crude product. Finally, yields of 23% for **3** and 66% for **2** were obtained according to ^1^H NMR spectroscopy using 1,3,5-trimethoxybenzene as an internal standard.

#### 3.3.5. Experimental Procedures for **2** to **3** with Standard Conditions and 4Å Molecular Sieves

A 10 mL dried tube was charged with **2** (0.1 mmol, 1.0 equiv), [Rh(COD)_2_]BF_4_ (6.0 mol %), Binap (6.0 mol %) and 100 mg 4Å molecular sieves. The reaction tube was evacuated and backfilled with argon (repeated three times). Then, PhCl (2.0 mL) was added into the tube. The reaction mixture was stirred at 120 °C for 4 h. After the reaction was completed, the reaction mixture was cooled to ambient temperature. Then, the organic solvent was removed under reduced pressure to afford the crude product. Finally, less than 10% of **3** was obtained according to ^1^H NMR spectroscopy.

#### 3.3.6. Experimental Procedures for **2** to **3** with HBF_4_

Four 10 mL dried tubes were charged with **2** (0.1 mmol, 1.0 equiv). The reaction tube was evacuated and backfilled with argon (repeated three times). Then, PhCl (1.0 mL) and 50% wt HBF_4_ (aqueous) (0.01 mmol, 0.1 equiv) were added into these tubes. The reaction mixture was stirred at room temperature, 60 °C, 100 °C and 120 °C for 4 h. After the reaction was completed, the reaction mixture was cooled to ambient temperature. The solvent was removed under reduced pressure, and the residue was purified using flash column chromatography (SiO_2_). The product was analyzed with ^1^H NMR spectroscopic data. At all temperatures, almost no product **3** was observed, and product **2** was gradually converted to product **4** as the temperature increased.

### 3.4. Experimental Procedures for Control Experiments to Monitor the Course of Reaction

#### 3.4.1. Experimental Procedures for **1** to **3** with Standard Conditions

Five 10 mL dried tubes were charged with **1** (0.1 mmol, 1.0 equiv), [Rh(COD)_2_]BF_4_ (6.0 mol %) and Binap (6.0 mol %). The reaction tube was evacuated and backfilled with argon (repeated three times). Then, 2.0 mL of the mixed solvent (toluene:DMF = 19:1) was added into all tubes. The reaction mixture was stirred at 120 °C for 1 h, 1.5 h, 2.0 h, 2.5 h and 3 h, respectively. A set of reaction mixtures were concentrated in vacuo and analyzed with ^1^H NMR spectra using 1,3,5-trimethoxybenzene as an internal standard.

#### 3.4.2. Experimental Procedures for **1** to **3** with Standard Conditions and Water

Five 10 mL dried tubes were charged with **1** (0.1 mmol, 1.0 equiv), [Rh(COD)_2_]BF_4_ (6.0 mol %), Binap (6.0 mol %) and 1.0 equiv H_2_O. The reaction tube was evacuated and backfilled with argon (repeated three times). Then, 2.0 mL of the mixed solvent (toluene:DMF = 19:1) was added into all tubes. The reaction mixture was stirred at 120 °C for 1 h, 1.5 h, 2.0 h, 2.5 h and 3 h, respectively. A set of reaction mixtures were concentrated in vacuo and analyzed with ^1^H NMR spectra using 1,3,5-trimethoxybenzene as an internal standard.

#### 3.4.3. Experimental Procedures for **2** to **3** with Standard Conditions

Five 10 mL dried tubes were charged with **2** (0.1 mmol, 1.0 equiv), [Rh(COD)_2_]BF_4_ (6.0 mol %) and Binap (6.0 mol %). The reaction tube was evacuated and backfilled with argon (repeated three times). Then, 2.0 mL PhCl was added into all tubes. The reaction mixture was stirred at 120 °C for 20 min, 40 min, 60 min, 80 min and 100 min, respectively. A set of reaction mixtures were concentrated in vacuo and analyzed with ^1^H NMR spectra using 1,3,5-trimethoxybenzene as an internal standard.

#### 3.4.4. Experimental Procedures for **2** to **3** with Standard Conditions and Water

Five 10 mL dried tubes were charged with **2** (0.1 mmol, 1.0 equiv), [Rh(COD)_2_]BF_4_ (6.0 mol %), Binap (6.0 mol %) and 1.0 equiv H_2_O. The reaction tube was evacuated and backfilled with argon (repeated three times). Then, 2.0 mL PhCl was added into all tubes. The reaction mixture was stirred at 120 °C for 20 min, 40 min, 60 min, 80 min and 100 min, respectively. A set of reaction mixtures were concentrated in vacuo and analyzed with ^1^H NMR spectra using 1,3,5-trimethoxybenzene as an internal standard.

### 3.5. Computational Methods

All calculations were performed using the Gaussian 16 program [35]. The geometries of all minima and transition states were optimized using the BMK pure functional [36] and the def2SVP basis set [37,38] for all atoms. The BMK pure functional was found to perform well in rhodium-catalyzed cycloaddition reactions [39,40]. The subsequent frequency calculations on stationary points were carried out at the same level of theory to ascertain the nature of the stationary points as minima or first-order saddle points on the respective potential energy surfaces. Zero point energy (ZPE) correction is included in the frequency calculations. All transition states were characterized by only one imaginary frequency pertaining to the desired reaction coordinate. Intrinsic reaction coordinate (IRC) calculations were carried out at the same level of theory to further authenticate the transition states. Thermochemical corrections to 298.15 K were calculated for all minima from unscaled vibrational frequencies obtained at the same level. The solvent effect was estimated using the IEFPCM method with radii and non-electrostatic terms for SMD solvation model in PhCl (ε = 5.6968) [41]. Solvation single-point computations utilized the def2TZVPP basis set for all atoms. The 3D images of the calculated structure were prepared using CYLView [42].

Each of the molecules presented in this study has an electronic multiplicity of 1. They are all in a singlet state. The total charge of the molecules involving rhodium complexes is 1, while the total charge of the molecules without rhodium complexes is 0. The Cartesian coordinates and energies of all optimized structures are shown on page S26 in the Supporting Information.

## 4. Conclusions

**Scheme 10:**
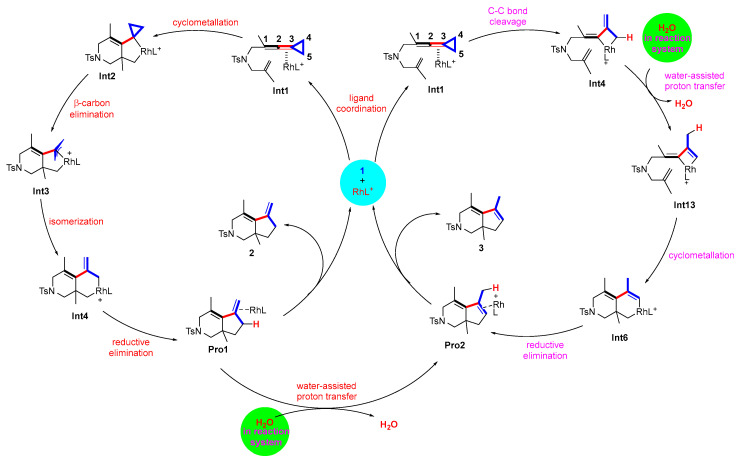
The most plausible reaction mechanism.

In summary, we conducted a series of control experiments and monitored the generation course of the *exo* double bond product **2** and *endo* double bond product **3** during the reaction; we subsequently found that trace amounts of water in the reaction system, including the rhodium catalyst, substrate and solvent, were sufficient to promote the formation of product **3**, and additional water could not further accelerate the reaction to generate product **3**. The water in the reaction did not have a significant influence on the generation of product **2**. The DFT calculation results are in line with experimental results. The most plausible reaction mechanism is summarized in Scheme 10. First, under the standard conditions at 80 °C, substrate **1** is coordinated with the rhodium catalyst to obtain the intermediate **Int1**. After cyclometallation, β-carbon elimination and reductive elimination, **Pro1** is obtained, which releases the rhodium catalyst to generate product **2**. Under the standard conditions at 120 °C, **Pro1** preferably undergoes water-assisted proton transfer to produce **Pro2**, which releases the rhodium catalyst to generate product **3**. Meanwhile, **Pro2** may also be obtained from **Int1** via C4–C5 bond cleavage, water-assisted proton transfer, cyclometallation and reductive elimination, which release the rhodium catalyst to generate product **3**.

In conclusion, without the assistance of water, the generation of product **2** is kinetically favorable, whereas product **3** is difficult to obtain because of its high energy barrier. When water is present, product **3** can be converted via proton transfer of product **2** under standard conditions or via water-assisted proton transfer in Path b rather than from **Int3**. This mechanistic insight of trace amounts of water assisting proton transfer will provide a deeper understanding of subsequent related studies.

## Data Availability

Data are contained within the article and Supplementary Materials.

## Supplementary Materials

The following supporting information can be downloaded at: https://www.mdpi.com/article/10.3390/molecules29051085/s1, Table S1: The yield of products **2** and **3** at 0 h, 1 h, 1.5 h, 2 h, 2.5 h, 3 h, respectively; Table S2: The yield of products **2** and **3** at 0 h, 1 h, 1.5 h, 2 h, 2.5 h, 3 h, respectively; Table S3: The yield of products **2** and **3** at 0 min, 20 min, 40 min, 60 min, 80 min, and 100 min, respectively; Table S4: The yield of products **2** and **3** at 0 min, 20 min, 40 min, 60 min, 80 min, and 100 min, respectively; Table S5: The total energies, enthalpies and free energies of all species in PhCl, ε = 5.6968 [43].
