# Supplementary material for: Mechanistic Studies on Rhodium-Catalyzed Chemoselective Cycloaddition of Ene-Vinylidenecyclopropanes: Water-Assisted Proton Transfer
Source: Molecules. 2024 Feb 29;29(5):1085. doi: 10.3390/molecules29051085 (PMC10934677; doi:10.3390/molecules29051085)
Supplement: Supplementary file 1 [file molecules-29-01085-s001.zip › molecules-2881838-supplementary.pdf]

Supporting Information

Mechanistic Studies on Rhodium-Catalyzed Chemoselective Cycloaddition of Ene-Vinylidenecyclopropanes: Water-Assisted Proton Transfer

Ziqi Yu [1], Min Shi [1,2]* and Yin Wei [2,]*

[1] Key Laboratory for Advanced Materials and Institute of Fine Chemicals, School of Chemistry and Molecular Engineering, East China University of Science and Technology, Meilong Road No. 130, Shanghai 200237, China; 18321678772@163.com

[2] State Key Laboratory of Organometallic Chemistry, Center for Excellence in Molecular Synthesis, Shanghai Institute of Organic Chemistry, Chinese Academy of Sciences, University of Chinese Academy of Sciences, 345 Lingling Road, Shanghai 200032, China

* Correspondence: mshi@mail.sioc.ac.cn (M.S.); weiyin@sioc.ac.cn (Y.W.)

Contents

1. General remarks S3
2. Characterization data of compounds **1**, **2**, **3** and **4** .... S**Error! Bookmark not defined.**
3. Control experiments S12
4. Conctrol experiment with water .... S**Error! Bookmark not defined.**
5. Control experiments to monitor the course of the reaction S17
6. Mass fraction of water in the Rh catalyst and substrate **1** S21
7. Computational details S24
8. Cartesian coordinates and energies of all optimized structures……………………… …..…...S26
9. References……………………………………………………………………………..S108

1. General remarks

^{1}H and ^{13}C NMR spectra were recorded at 400 MHz and 600 MHz. Catalysts $[Rh(COD)_2]BF_4$ and ligands BINAP were purchased from Pepper Reagent. Toluene was distilled from sodium (Na) under argon (Ar) atmosphere. Super dry PhCl was purchased from Meryer, which contains ≤ 50 ppm of water, and super dry DMF and HBF_4(aq) was purchased from General-reagent®. Commercially obtained reagents were used without further purification. All reactions were monitored by TLC with silica gel coated plates (Huanghai GF254). Flash column chromatography was performed by using 300-400 mesh silica gel eluting with ethyl acetate and petroleum ether at increased pressure. The content of water was determined by Metrohm 831KF.

2. Characterization data of compounds 1, 2, 3 and 4

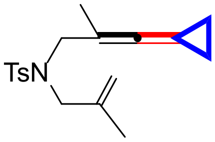

Compound 1: This is a known compounds and its spectroscopic data are consistent with those reported in the previous literature.[1] A light yellow oil, ^{1}H NMR ($CDCl_3$, 600 MHz, TMS) δ 1.32-1.34 (m, 2H), 1.39-1.42 (m, 2H), 1.63 (s, 6H), 2.41 (s, 3H), 3.75 (s, 2H), 3.80 (s, 2H), 4.84 (s, 1H), 4.86 (s, 1H) 7.27 (d, J = 7.8 Hz, 2H), 7.70 (d, J = 8.4 Hz, 2H). ^{13}C NMR ($CDCl_3$, 150 MHz, TMS) δ 6.7, 17.1, 20.0, 21.5, 51.1, 52.8, 77.6, 97.4, 114.4, 127.2, 129.4, 137.7, 140.1, 142.9, 188.7.

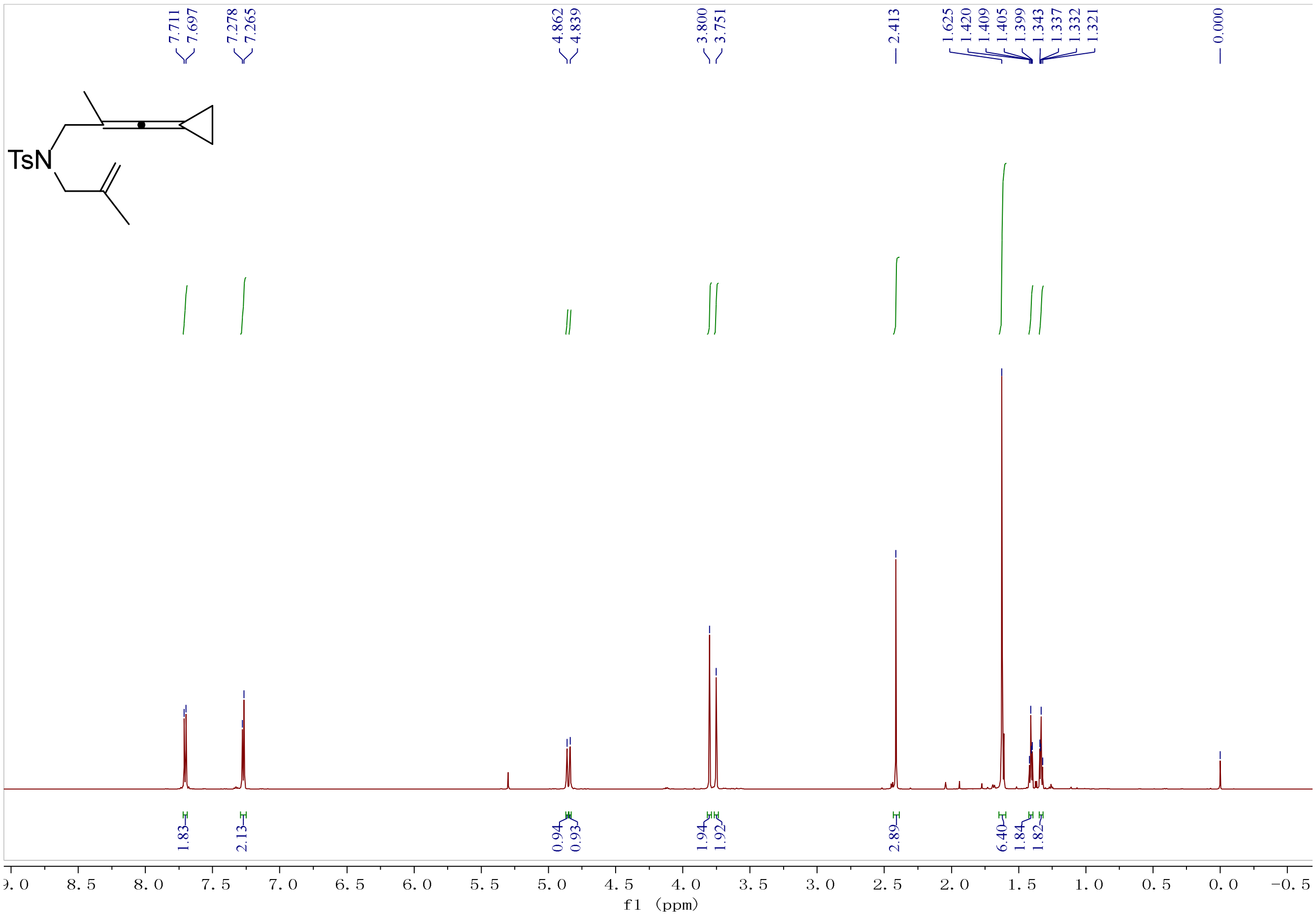

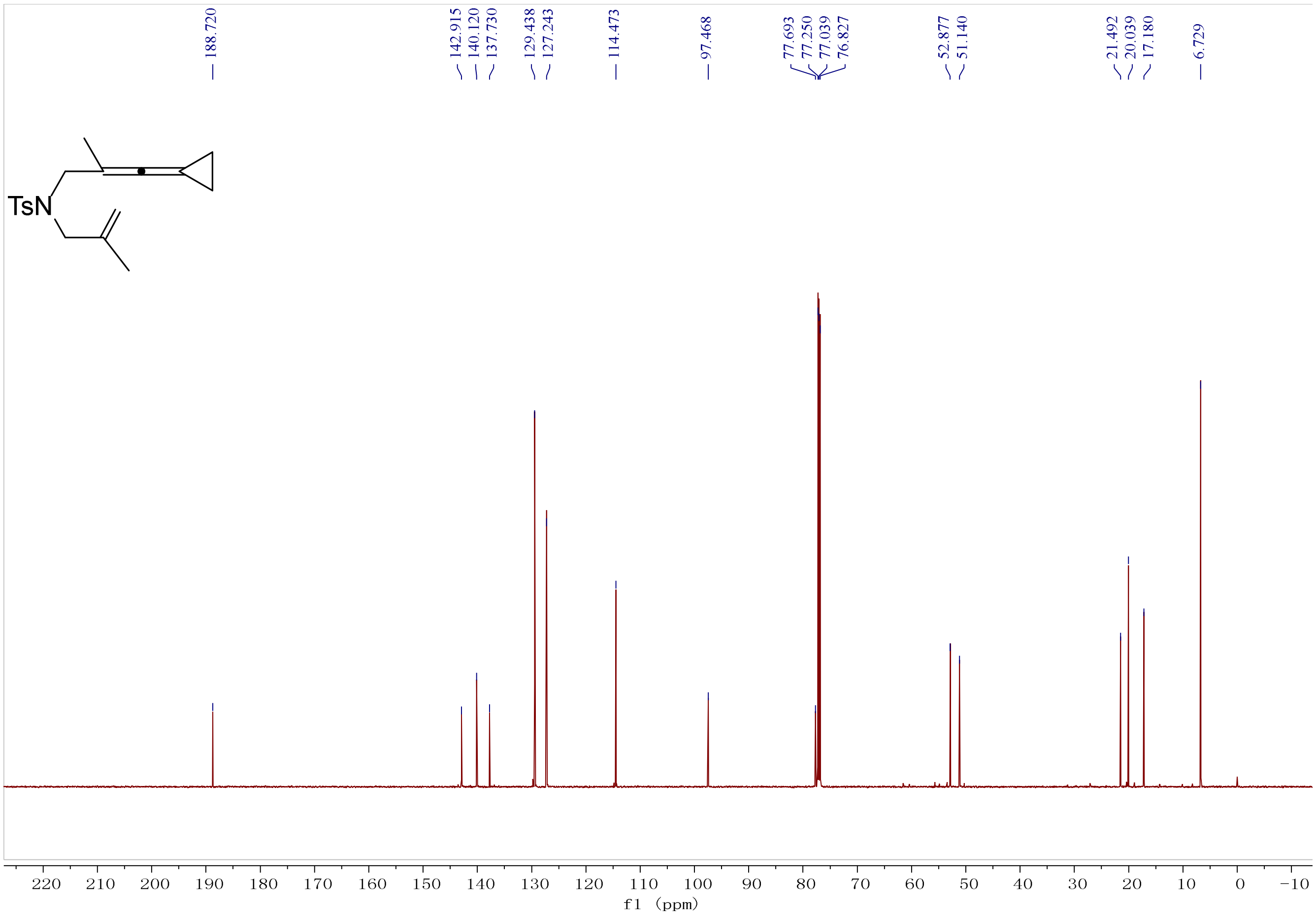

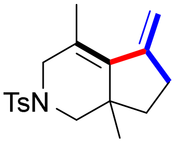

Compound 2: This is a known compounds and its spectroscopic data are consistent with those reported in the previous literature.[1] A white solid, ^{1}H NMR (600 MHz, $CDCl_3$, TMS) δ 1.13 (s, 3H), 1.21-1.29 (m, 1H), 1.57-1.62 (m, 1H), 1.75 (s, 3H), 2.11 (d, *J* = 10.8 Hz, 1H), 2.37-2.43 (m, 4H), 2.56-2.65 (m, 1H), 2.97 (d, *J* = 16.8 Hz, 1H), 3.77 (d, *J* = 10.8 Hz, 1H), 4.03 (d, *J* = 16.8 Hz, 1H), 5.12 (s, 2H), 7.30 (d, *J* = 7.8 Hz, 2H), 7.69 (d, *J* = 8.4 Hz, 2H). ^{13}C NMR (150 MHz, $CDCl_3$, TMS) δ 17.0, 21.5, 23.4, 30.3, 35.1, 43.3, 49.6, 54.6, 110.3, 122.7, 127.6, 129.6, 133.5, 138.9, 143.4, 146.2.

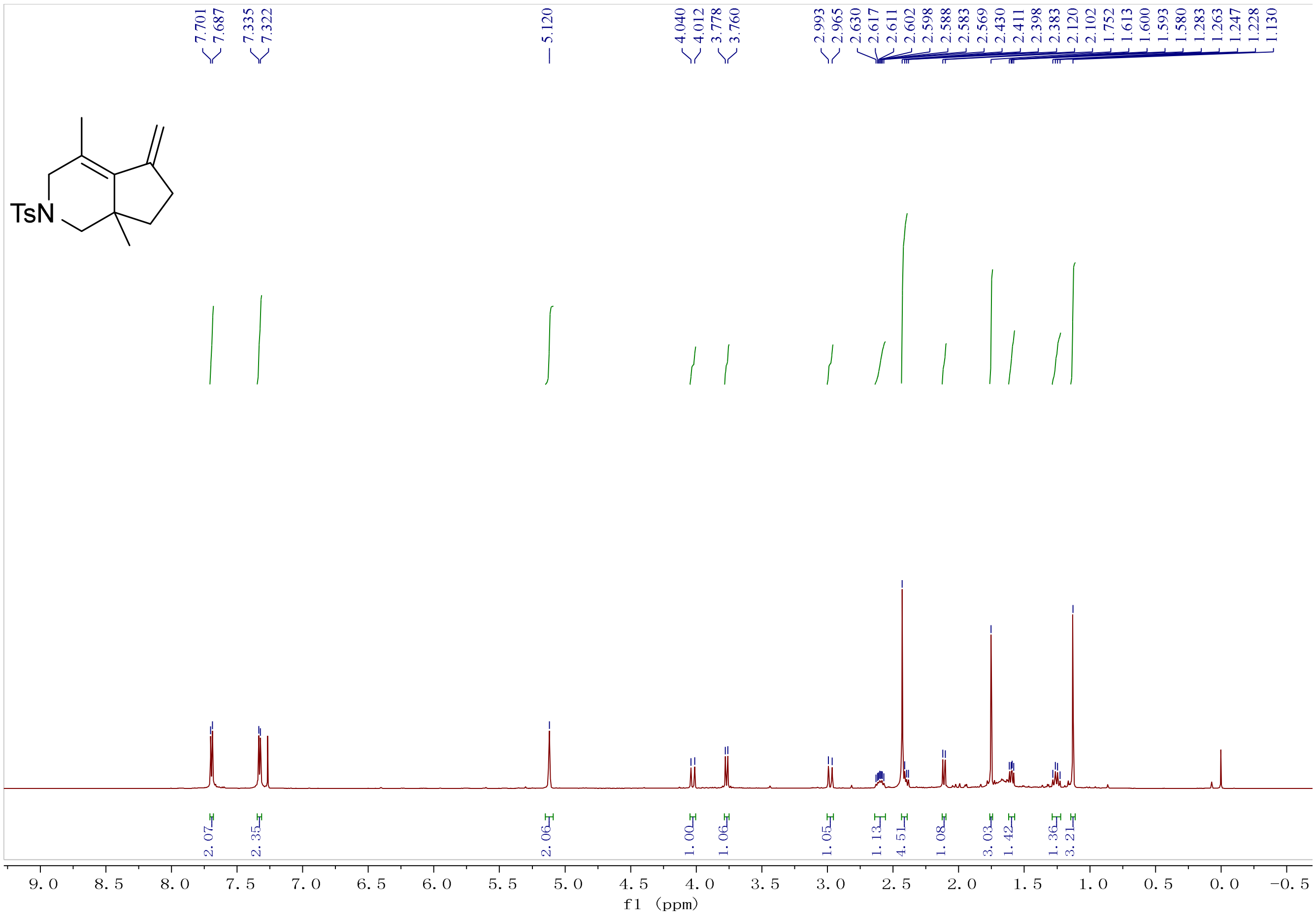
TsN
7.701
7.687
7.335
7.322
5.120
4.040
4.012
3.778
3.760
2.993
2.965
2.630
2.617
2.611
2.602
2.598
2.588
2.583
2.569
2.430
2.411
2.398
2.383
2.120
2.102
1.752
1.613
1.600
1.593
1.580
1.283
1.263
1.247
1.228
1.130
2.07
2.35
2.06
1.00
1.06
1.05
1.13
4.51
1.08
3.03
1.42
1.36
3.21
9.0 8.5 8.0 7.5 7.0 6.5 6.0 5.5 5.0 4.5 4.0 3.5 3.0 2.5 2.0 1.5 1.0 0.5 0.0 -0.5
f1 (ppm)

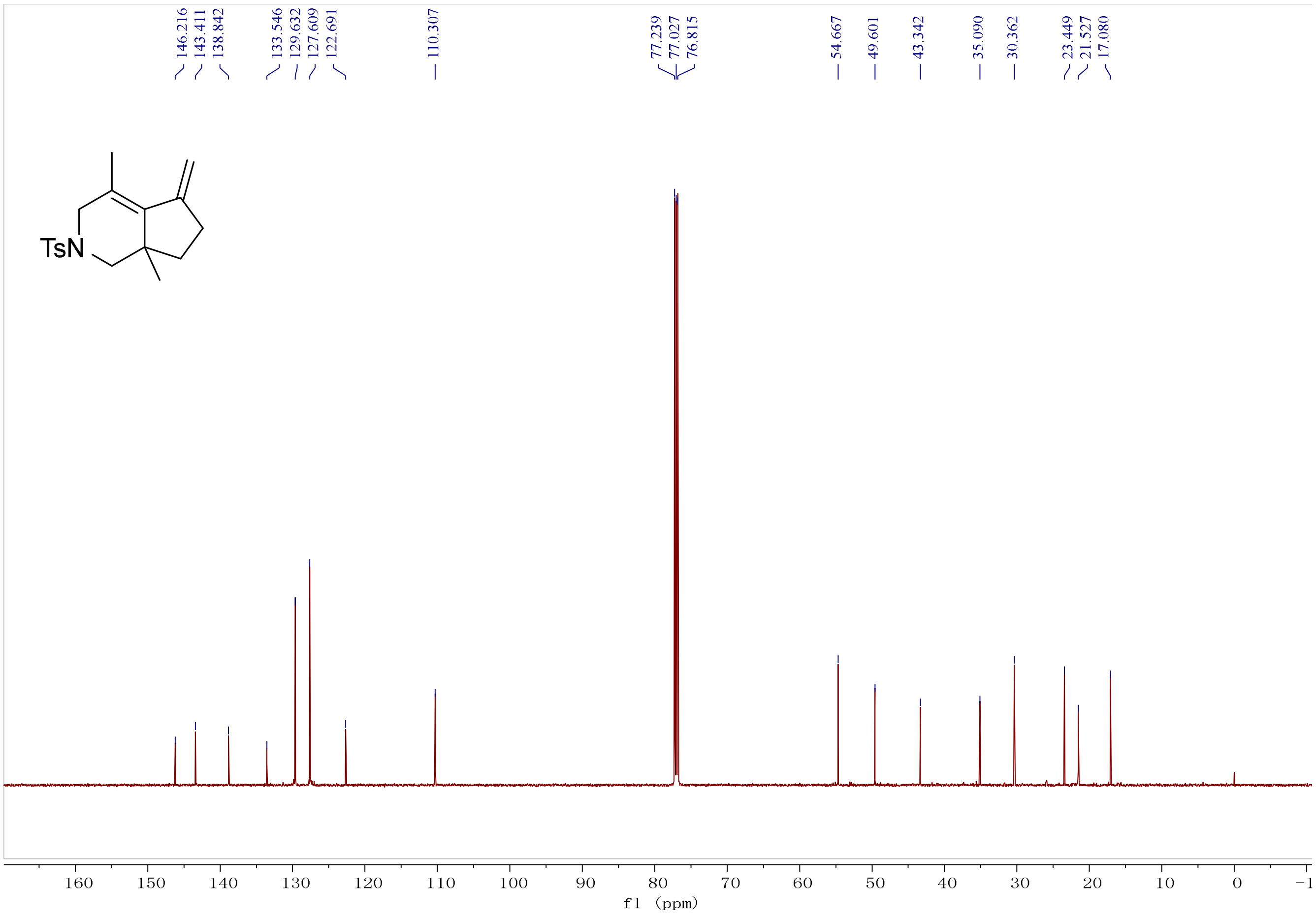

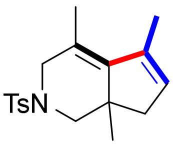

Compound 3: This is a known compounds and its spectroscopic data are consistent with those reported in the previous literature.[1] A white solid, ^{1}H NMR (400 MHz, $CDCl_3$, TMS) δ 1.17 (s, 3H),1.73 (s, 3H), 1.95 (s, 3H), 2.07 (q, J = 15.6 Hz, 2H), 2.39 (d, J = 10.4 Hz, 1H), 2.42 (s, 3H), 3.06 (d, J = 16.0 Hz, 1H), 3.55 (d, J = 10.8 Hz, 1H), 3.94 (d, J = 16.4 Hz, 1H), 5.60 (s, 1H), 7.32 (d, J = 8.0 Hz, 2H), 7.69 (d, J = 8.0 Hz, 2H). ^{13}C NMR (100 MHz, $CDCl_3$, TMS) δ 16.0, 17.3, 21.5, 26.0, 41.7, 44.0, 48.8, 53.0, 117.1, 127.5, 129.5, 131.3, 133.8, 138.4, 143.2, 143.6.

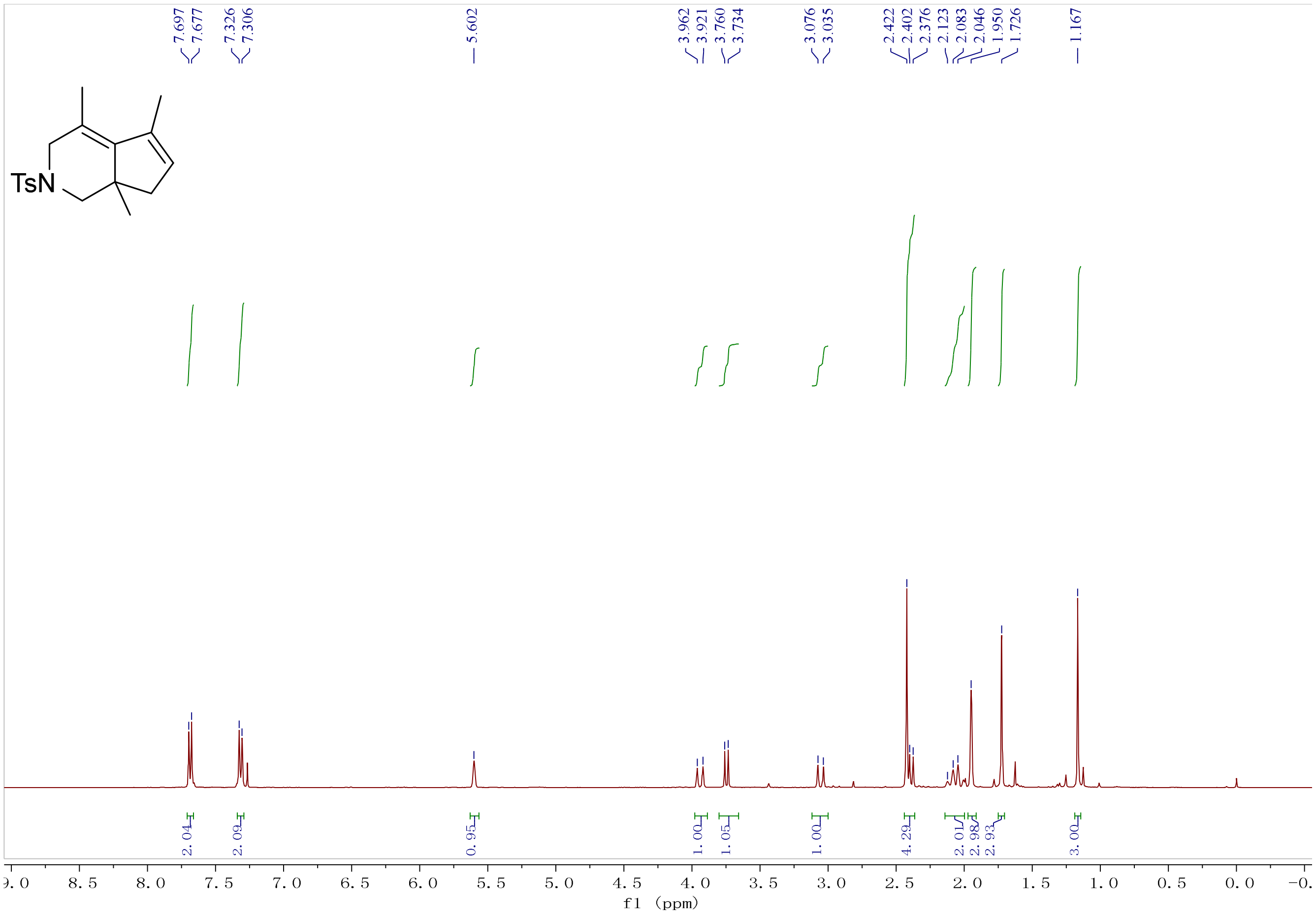

7.697
7.677
7.326
7.306
5.602
3.962
3.921
3.760
3.734
3.076
3.035
2.422
2.402
2.376
2.123
2.083
2.046
1.950
1.726
1.167
TsN
2.04
2.09
0.95
1.00
1.05
1.00
4.29
2.01
2.98
2.93
3.00
f1 (ppm)

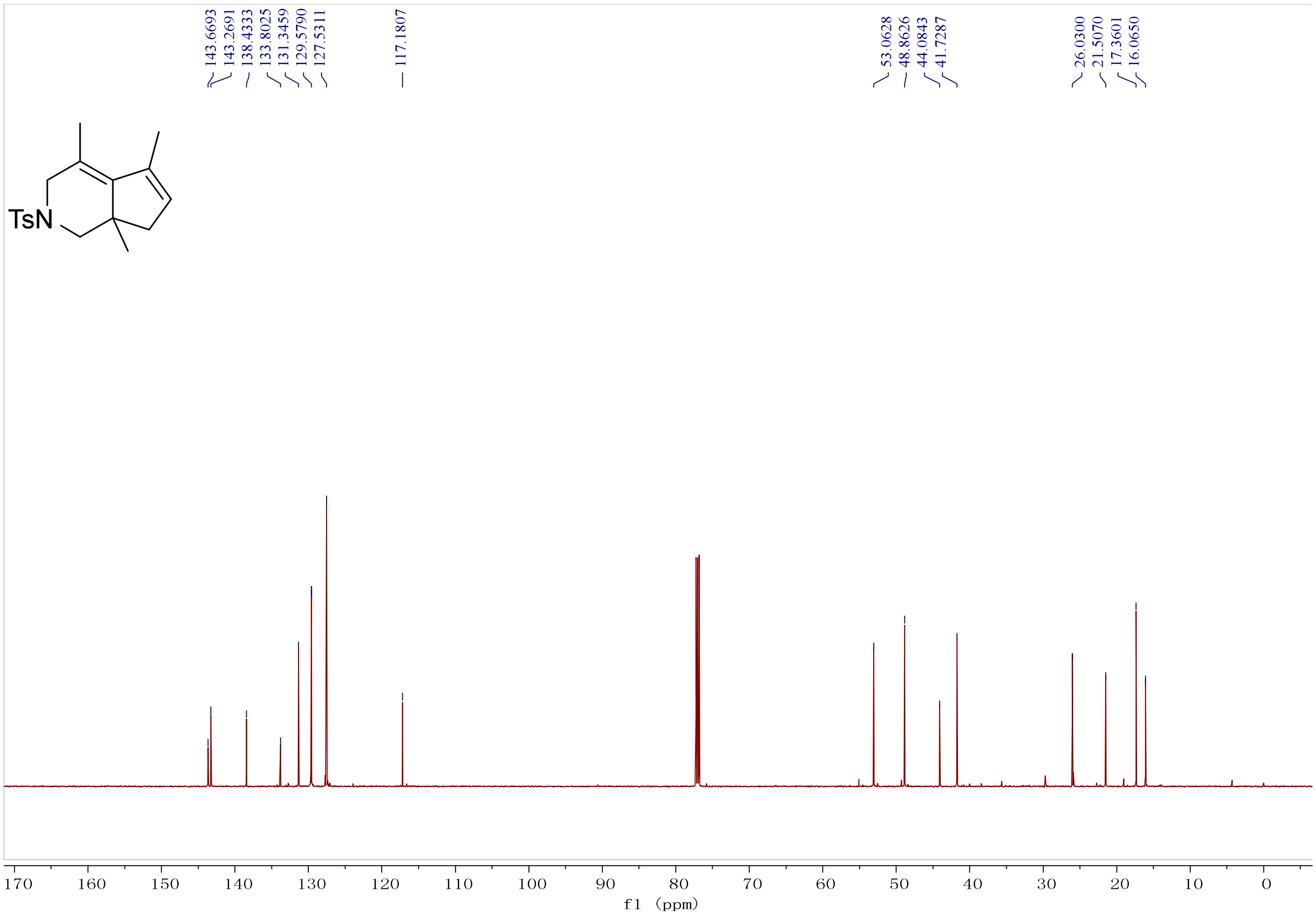

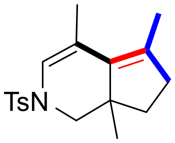

Compound 4: A white solid. M. P. 130 - 132 ºC. ^{1}H NMR (400 MHz, $CDCl_3$, TMS) δ 0.86 (s, 3H),1.37 - 1.45 (m, 1H), 1.64 - 1.69 (m, 1H), 1.83 (s, 1H), 1.94 (s, 1H), 2.08 - 2.15 (m, 1H), 2.41 (s, 1H), 2.50 - 2.56 (m, 2H), 3.73 (d, *J* = 10.8 Hz, 1H), 6.40 (s, 1H), 7.29 (d, *J* = 8.0 Hz, 2H), 7.67 (d, *J* = 8.4 Hz, 2H). ^{13}C NMR (101 MHz, $CDCl_3$, TMS) δ 15.7, 19.4, 21.5, 21.6, 35.6, 37.4, 45.9, 55.3, 112.0, 120.9, 127.0, 129.7, 130.9, 133.8, 135.3.2, 143.5. IR (neat) *v* 2958, 2924, 2856, 1609, 1457, 1347, 1213, 1092, 1012, 946 cm^{-1}. HRMS (ESI) calcd. for $C_{18}H_{23}NO_2NaS$ $(M+Na)^+$: 340.1342, Found: 340.1341.

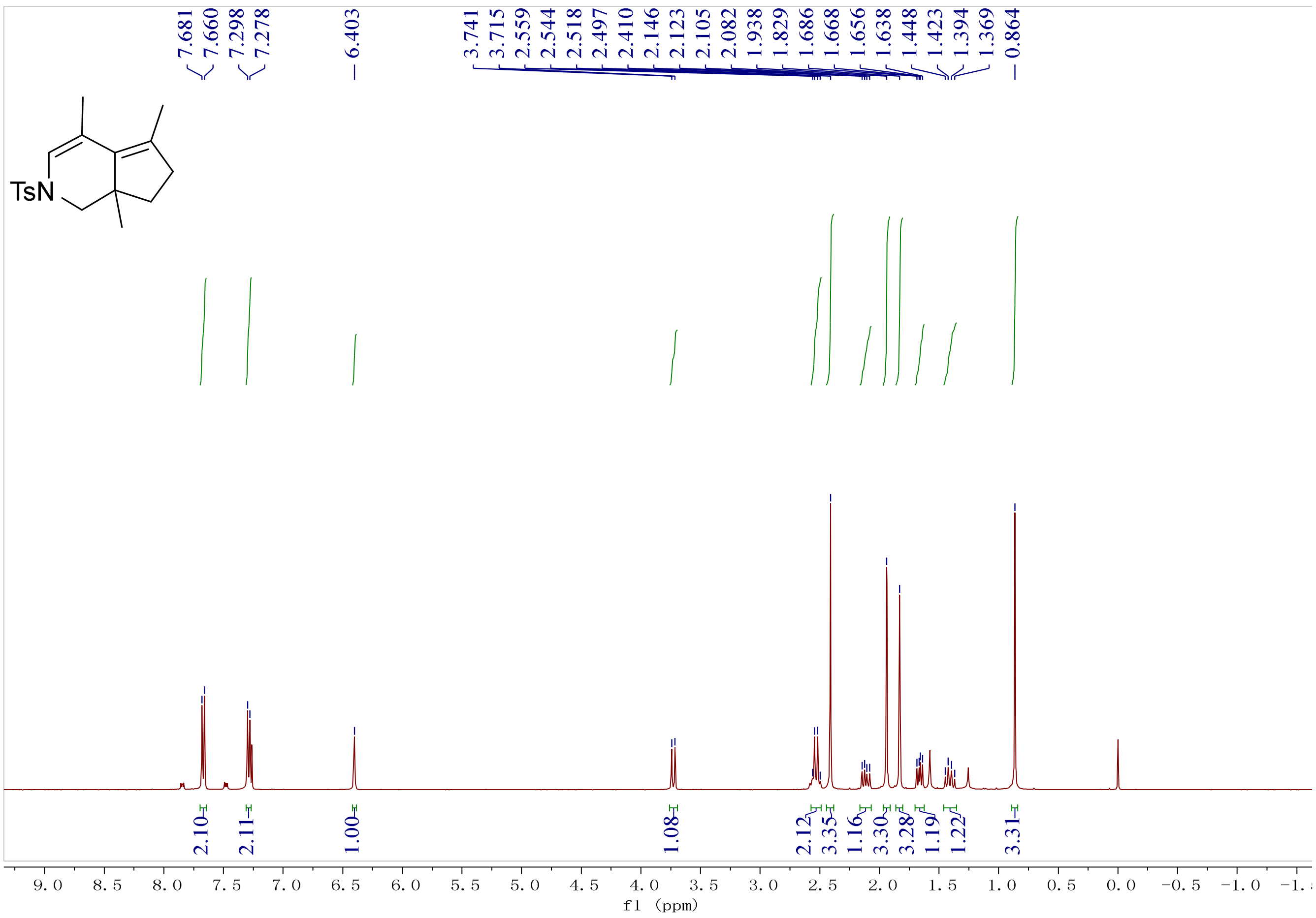

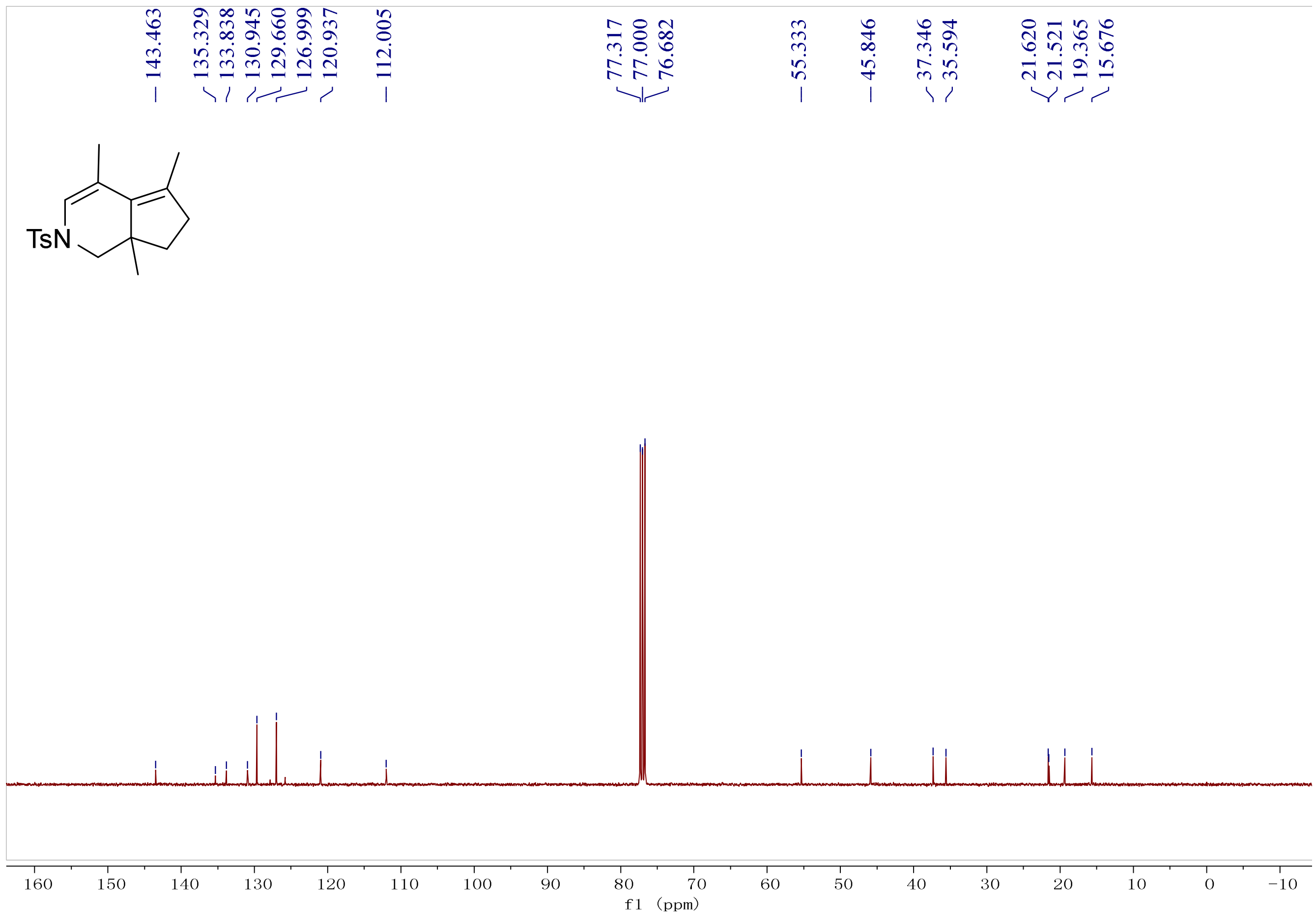
143.463
135.329
133.838
130.945
129.660
126.999
120.937
112.005
77.317
77.000
76.682
55.333
45.846
37.346
35.594
21.620
21.521
19.365
15.676
TsN
f1 (ppm)

3. Control experiments

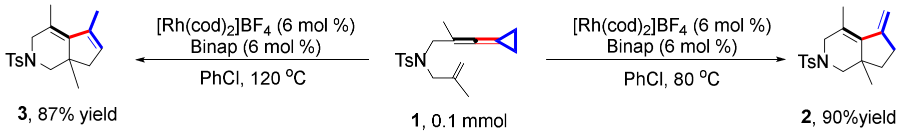

To a 10 mL dried tube was charged with **1** (0.1 mmol, 1.0 equiv), $[Rh(COD)_2]BF_4$ (6.0 mol %) and Binap (6.0 mol %). The reaction tube was evacuated and backfilled with argon (repeated three times). Then, PhCl (2.0 mL) was added into the tube. The reaction mixture was stirred at 80 °C for 12 h. The solvent was removed under reduced pressure and the residue was purified by a flash column chromatography (SiO_2) to give the corresponding product **2** (28.5 mg, 90%).

To a 10 mL dried tube was charged with **1** (0.1 mmol, 1.0 equiv), $[Rh(COD)_2]BF_4$ (6.0 mol %) and Binap (6.0 mol %). The reaction tube was evacuated and backfilled with argon (repeated three times). Then, PhCl (2.0 mL) was added into the tube. The reaction mixture was stirred at 120 °C for 4 h. The solvent was removed under reduced pressure and the residue was purified by a flash column chromatography (SiO_2) to give the corresponding product **3** (27.6 mg, 87%).

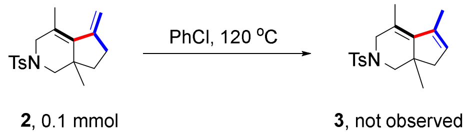

To a 10 mL dried tube was charged with **2** (0.1 mmol, 1.0 equiv). The reaction tube was evacuated and backfilled with argon (repeated three times). Then, PhCl (2.0 mL) was added into the tube. The reaction mixture was stirred at 120 °C for 4 h. The solvent was removed under reduced pressure and the residue was purified by a flash column chromatography (SiO_2). The product was confirmed by ^{1}H NMR spectroscopic data. None of product **3** was observed.

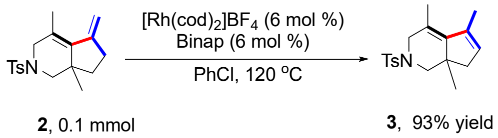

To a 10 mL dried tube was charged with **2** (0.1 mmol, 1.0 equiv), $[Rh(COD)_2]BF_4$ (6.0 mol %) and Binap (6.0 mol %). The reaction tube was evacuated and backfilled with argon (repeated three times). Then, PhCl (2.0 mL) was added into the tube. The reaction mixture was stirred at 120 °C for 4 h. The solvent was removed under reduced pressure and the residue was purified by a flash column

chromatography (SiO_2) to give the corresponding product **3** (29.4 mg, 93%). This indicates that product **2** can be converted to product **3** at 120 °C only under the standard conditions.

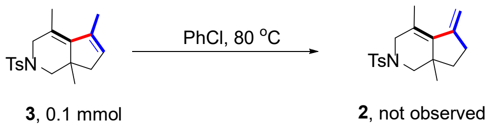

To a 10 mL dried tube was charged with **3** (0.1 mmol, 1.0 equiv). The reaction tube was evacuated and backfilled with argon (repeated three times). Then, PhCl (2.0 mL) was added into the tube. The reaction mixture was stirred at 80 °C for 12 h. The solvent was removed under reduced pressure and the residue was purified by a flash column chromatography (SiO_2). The product was analyzed by ^{1}H NMR spectroscopic data. None of product **2** was observed.

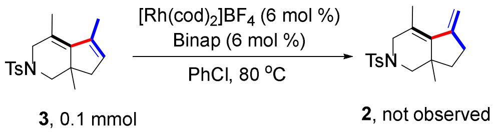

To a 10 mL dried tube was charged with **3** (0.1 mmol, 1.0 equiv), $[Rh(COD)_2]BF_4$ (6.0 mol %) and Binap (6.0 mol %). The reaction tube was evacuated and backfilled with argon (repeated three times). Then, PhCl (2.0 mL) was added into the tube. The reaction mixture was stirred at 80 °C for 12 h. The solvent was removed under reduced pressure and the residue was purified by a flash column chromatography (SiO_2). The product was confirmed by ^{1}H NMR spectroscopic data. None of product **2** was observed. It is suggested that product **3** cannot be converted to product **2** at 80 °C whether or not it is under the standard conditions.

4. Control experiment involving water.

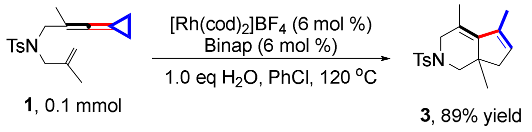

To a 10 mL dried tube was charged with **1** (0.1 mmol, 1.0 equiv), [Rh(COD)$_2$]BF$_4$ (6.0 mol %) , Binap (6.0 mol %) and H_2O (1.0 equiv). The reaction tube was evacuated and backfilled with argon (repeated three times). Then, PhCl (2.0 mL) was added into the tube. The reaction mixture was stirred at 120 °C for 4 h. The solvent was removed under reduced pressure and the residue was purified by a flash column chromatography (SiO_2) to give the corresponding product **3** (28.2 mg, 89%). There is no obvious difference between the two conditions of adding water and not adding water.

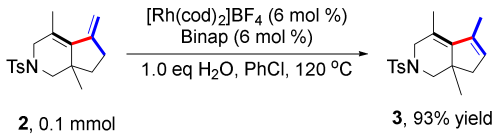

To a 10 mL dried tube was charged with **2** (0.1 mmol, 1.0 equiv), [Rh(COD)$_2$]BF$_4$ (6.0 mol %) , Binap (6.0 mol %) and H_2O (1.0 equiv). The reaction tube was evacuated and backfilled with argon (repeated three times). Then, PhCl (2.0 mL) was added into the tube. The reaction mixture was stirred at 120 °C for 4 h. The solvent was removed under reduced pressure and the residue was purified by a flash column chromatography (SiO_2) to give the corresponding product **3** (29.4 mg, 93%). This indicates that the addition of extra water did not have a significant effect on the conversion of **2** to **3**.

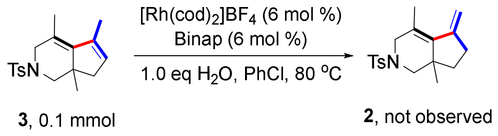

To a 10 mL dried tube was charged with **3** (0.1 mmol, 1.0 equiv), [Rh(COD)$_2$]BF$_4$ (6.0 mol %) , Binap (6.0 mol %) and H_2O (1.0 equiv). The reaction tube was evacuated and backfilled with argon (repeated three times). Then, PhCl (2.0 mL) was added into the tube. The reaction mixture was stirred at 120 °C for 4 h. The solvent was removed under reduced pressure and the residue was purified by a flash column chromatography (SiO_2). The product was analyzed by ^{1}H NMR spectroscopic data. None of product **2** was observed.

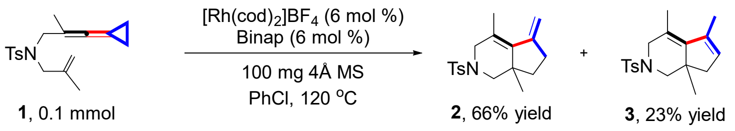

To a 10 mL dried tube was charged with **1** (0.1 mmol, 1.0 equiv), [Rh(COD)$_2$]BF$_4$ (6.0 mol %) , Binap (6.0 mol %) and 100 mg 4Å molecular sieves. The reaction tube was evacuated and backfilled with argon (repeated three times). Then, PhCl (2.0 mL) was added into the tube. The reaction mixture was stirred at 120 °C for 4 h. After the reaction was finished, the reaction mixture was cooled to ambient temperature. Then, organic solvent was removed under reduced pressure to afford the crude product. Finally, 23% yield of **3** and 66% yield of **2** was afforded according to the ^{1}H NMR spectroscopy, using 1,3,5-trimethoxybenzene as an internal standard.

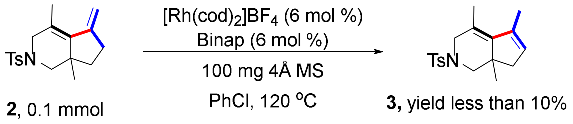

To a 10 mL dried tube was charged with **2** (0.1 mmol, 1.0 equiv), [Rh(COD)$_2$]BF$_4$ (6.0 mol %) , Binap (6.0 mol %) and 100 mg 4Å molecular sieves. The reaction tube was evacuated and backfilled with argon (repeated three times). Then, PhCl (2.0 mL) was added into the tube. The reaction mixture was stirred at 120 °C for 4 h. After the reaction was finished, the reaction mixture was cooled to ambient temperature. Then, organic solvent was removed under reduced pressure to afford the crude product. Finally, less than 10% of **3** was afforded according to the ^{1}H NMR spectroscopy.

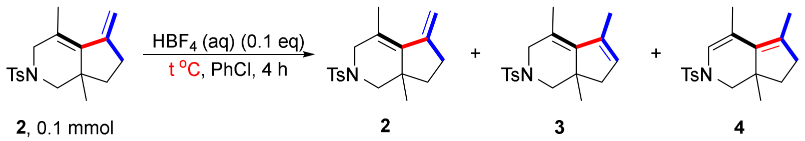

t/°C	r.t	60	100	120
**2** (yield)	50%	33%	trace	trace
**3** (yield)	trace	trace	trace	trace
**4** (yield)	48%	66%	>95%.	>95%

To four 10 mL dried tubes was charged with **2** (0.1 mmol, 1.0 equiv). The reaction tube was evacuated and backfilled with argon (repeated three times). Then, PhCl (1.0 mL) and 50% wt HBF$_4$ (aqueous) (0.01 mmol, 0.1 equiv) was added into these tubes. The reaction mixture was stirred at room temperature, 60 °C, 100 °C and 120 °C for 4 h. After the reaction was finished, the reaction mixture was cooled to ambient temperature. The solvent was removed under reduced pressure and the residue was

purified by a flash column chromatography (SiO_2). The product was analyzed by ^1H NMR spectroscopic data. At all temperatures, almost no product **3** was observed, and product **2** was gradually converted to product **4** as the temperature increased.

5. Control experiments to monitor the course of the reaction

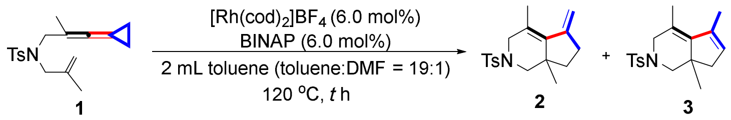

To five 10 mL dried tubes was charged with **1** (0.1 mmol, 1.0 equiv), $[Rh(COD)_2]BF_4$ (6.0 mol %), Binap (6.0 mol %). The reaction tube was evacuated and backfilled with argon (repeated three times). Then, 2.0 mL of mixed solvent (toluene:DMF = 19:1) was added into the all tubes. The reaction mixture was stirred at 120 °C for 1 h, 1.5 h, 2.0 h, 2.5 h and 3 h, respectively. A set of the reaction mixtures were concentrated in vacuo and analyzed by ^{1}H NMR spectrum using 1,3,5-trimethoxybenzene as an internal standard.

Table S1. The yield of products **2** and **3** at 0 h, 1 h, 1.5 h, 2 h, 2.5 h, 3 h, respectively

Time/h	0	1.0	1.5	2.0	2.5	3
**2**	0	56%	46%	41%	22%	15%
**3**	0	4%	8%	18%	36%	41%

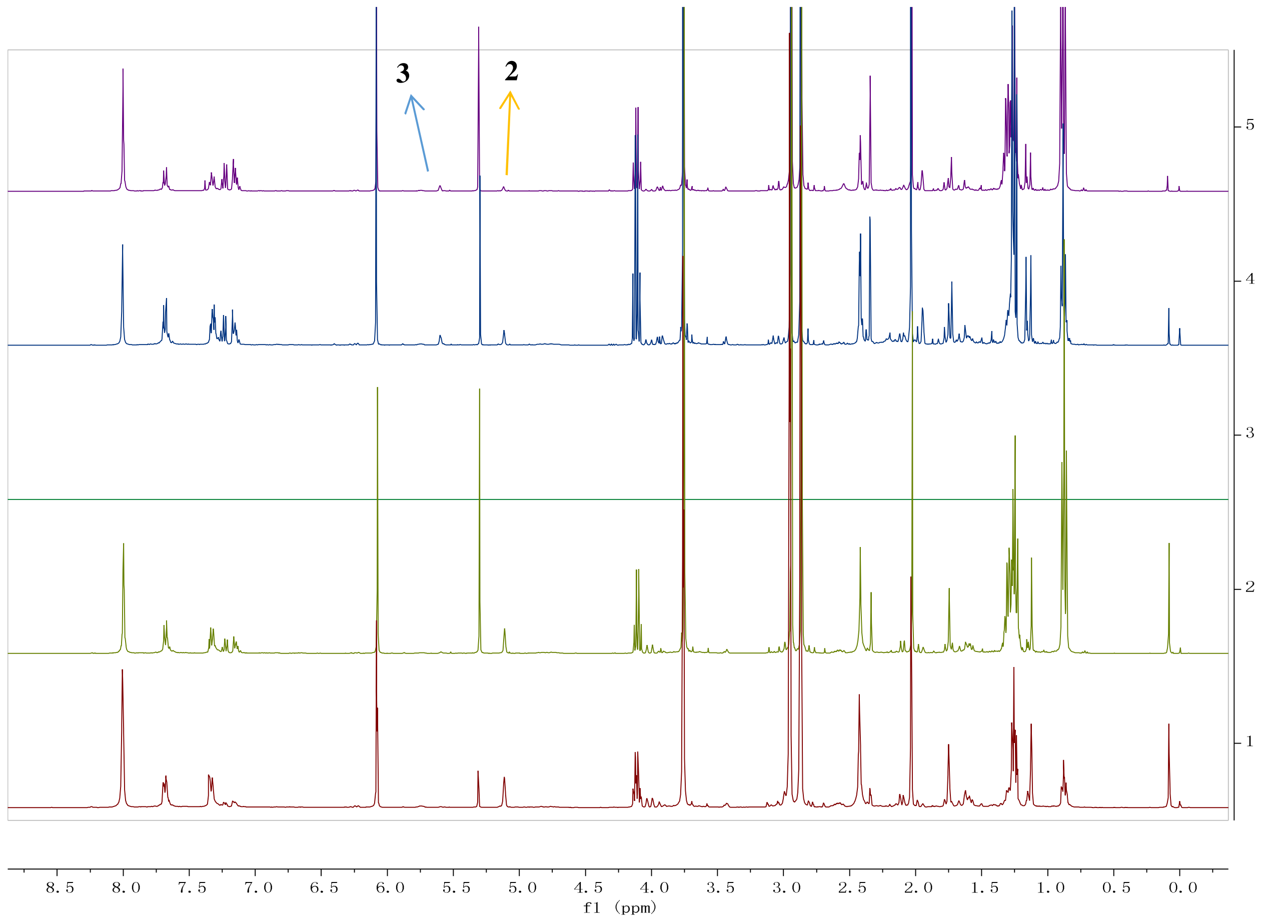

NMR crude spectra at 1 h, 1.5 h, 2 h, 2.5 h, and 3 h from bottom to top, respectively.

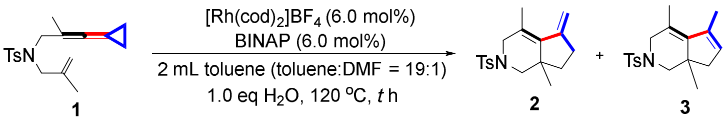

To five 10 mL dried tubes was charged with **1** (0.1 mmol, 1.0 equiv), [Rh(COD)$_2$]BF$_4$ (6.0 mol %), Binap (6.0 mol %) and 1.0 equiv H_2O. The reaction tube was evacuated and backfilled with argon (repeated three times). Then, 2.0 mL of mixed solvent (toluene:DMF = 19:1) was added into the all tubes. The reaction mixture was stirred at 120 °C for 1 h, 1.5 h, 2.0 h, 2.5 h and 3 h, respectively. A set of the reaction mixtures were concentrated in vacuo and analyzed by ^{1}H NMR spectrum using 1,3,5-trimethoxybenzene as an internal standard.

Table S2. The yield of products **2** and **3** at 0 h, 1 h, 1.5 h, 2 h, 2.5 h, 3 h, respectively

Time/h	0	1.0	1.5	2.0	2.5	3
**2**	0	40%	69.5%	64.5%	31.5%	13.5%
**3**	0	4%	8%	18%	29%	50%

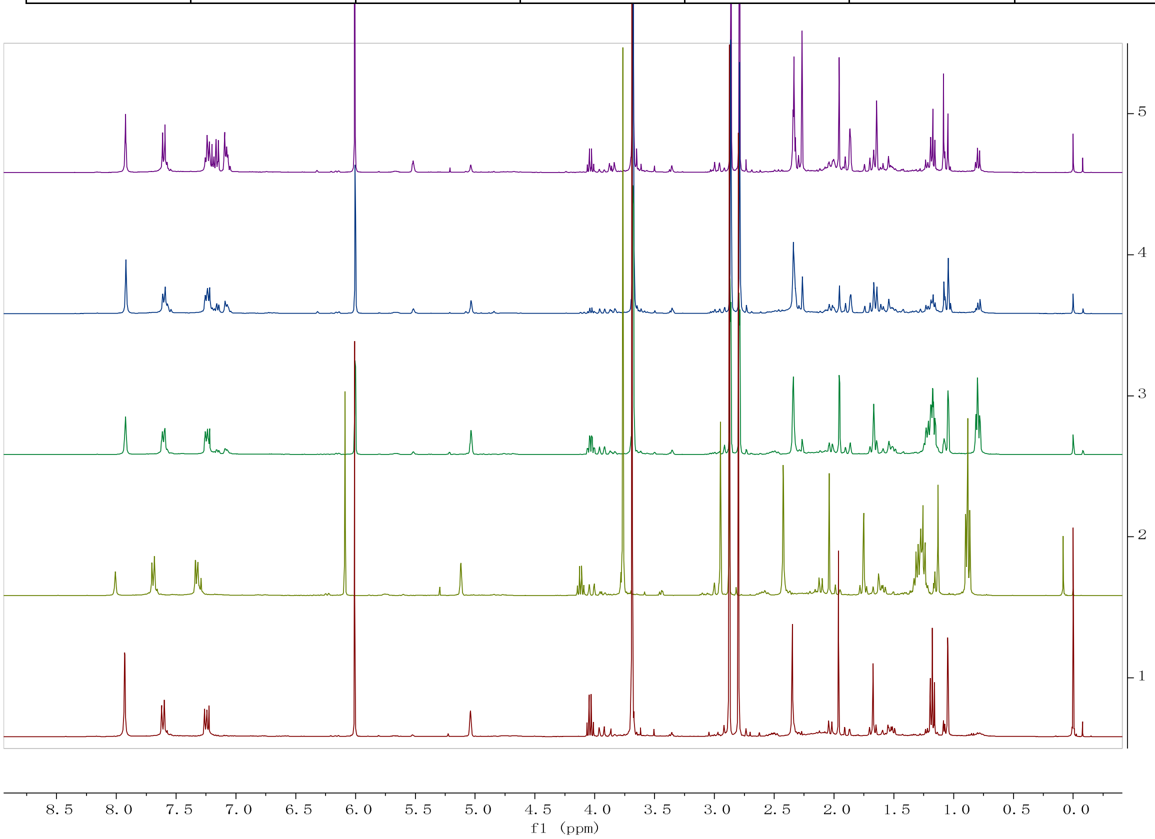

NMR crude spectra at 1 h, 1.5 h, 2 h, 2.5 h, and 3 h from bottom to top, respectively.

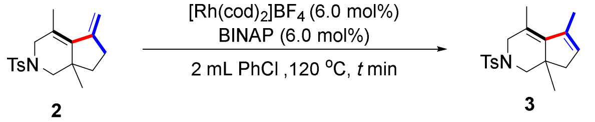

To five 10 mL dried tubes was charged with **2** (0.1 mmol, 1.0 equiv), [Rh(COD)$_2$]BF$_4$ (6.0 mol %), Binap (6.0 mol %). The reaction tube was evacuated and backfilled with argon (repeated three times). Then, 2.0 mL PhCl was added into the all tubes. The reaction mixture was stirred at 120 °C for 20 min, 40 min, 60 min, 80 min and 100 min, respectively. A set of the reaction mixtures were concentrated in vacuo and analyzed by ^{1}H NMR spectrum using 1,3,5-trimethoxybenzene as an internal standard.

Table S3. The yield of products **2** and **3** at 0 mine, 20 min, 40 min, 60 min, 80 min, and 100 min, respectively

Time/min	0	20	40	60	80	100
**2**	100%	54%	23%	9.5%	7%	6.5%
**3**	0	34%	68%	80%	82%	85%

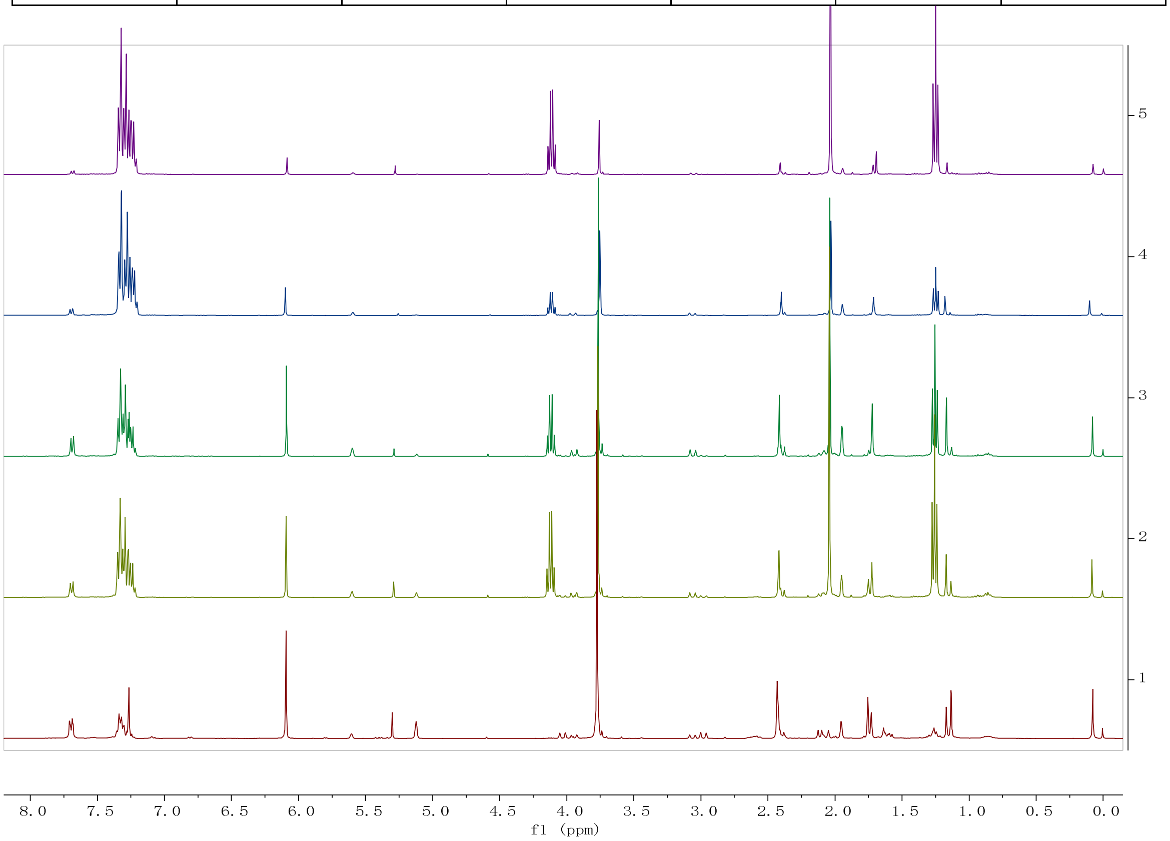

NMR crude spectra at 20 min, 40 min, 60 min, 80 min, and 100 min from bottom to top, respectively.

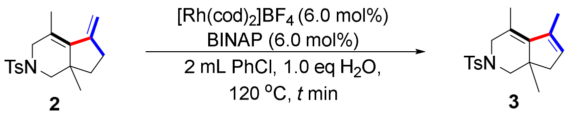

To five 10 mL dried tubes was charged with **2** (0.1 mmol, 1.0 equiv), $[Rh(COD)_2]BF_4$ (6.0 mol %), Binap (6.0 mol %) and 1.0 equiv H_2O. The reaction tube was evacuated and backfilled with argon (repeated three times). Then, 2.0 mL PhCl was added into the all tubes. The reaction mixture was stirred at 120 °C for 20 min, 40 min, 60 min, 80 min and 100 min, respectively. A set of the reaction mixtures were concentrated in vacuo and analyzed by ^{1}H NMR spectrum using 1,3,5-trimethoxybenzene as an internal standard.

Table S4. The yield of products **2** and **3** at 0 min, 20 min, 40 min, 60 min, 80 min, and 100 min, respectively

Time/min	0	20	40	60	80	100
**2**	100%	45%	22.5%	10.5%	7%	6.5%
**3**	0	47%	71%	80%	82%	84%

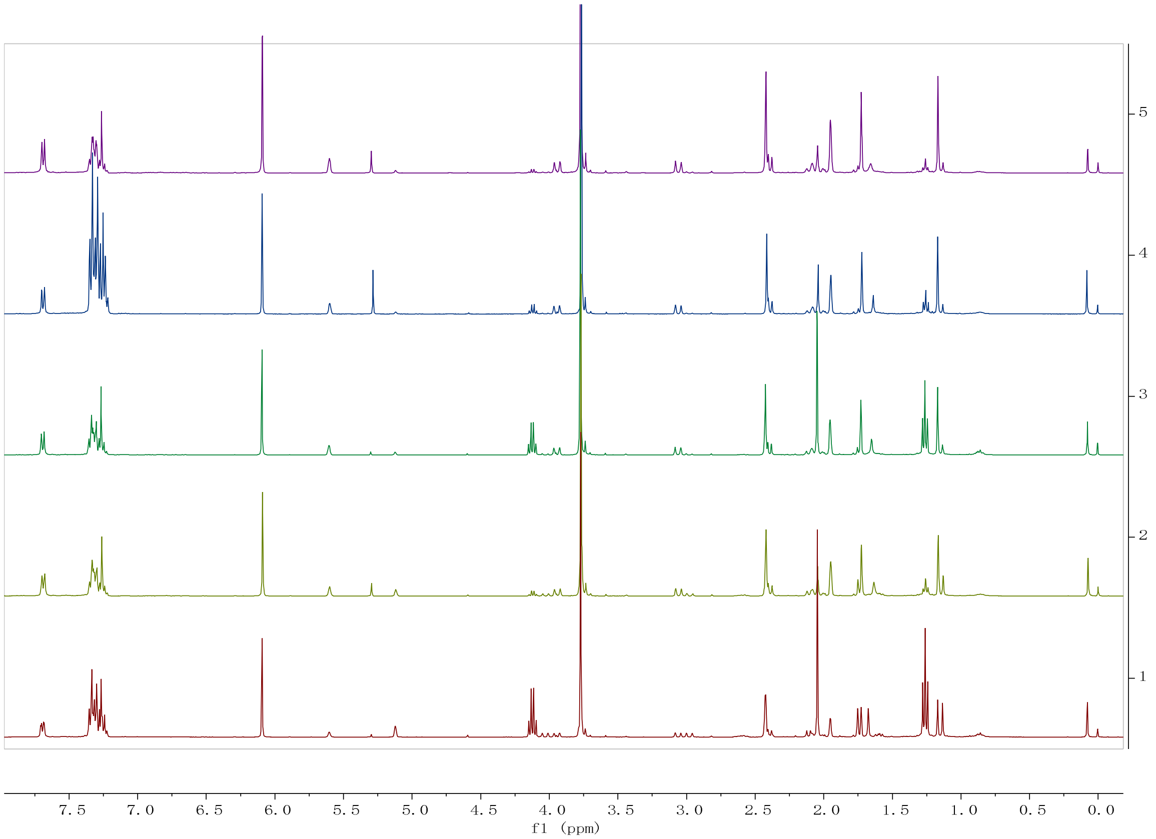

NMR crude spectra at 20 min, 40 min, 60 min, 80 min, and 100 min from bottom to top, respectively.

6. Mass fraction of water in Rh catalyst and substrate 1

The quantity of water was determined by Metrohm 831KF. The measurement principle is based on the Karl Fisher chemical reaction:[2]

$$CH_3OH + SO_2 + RN \longrightarrow [RNH]SO_3CH_3RN$$

$$I_2 + H_2O + [RNH]SO_3CH_3 + 2RN \longrightarrow [RNH]SO_4CH_3 + 2(RNH)I$$

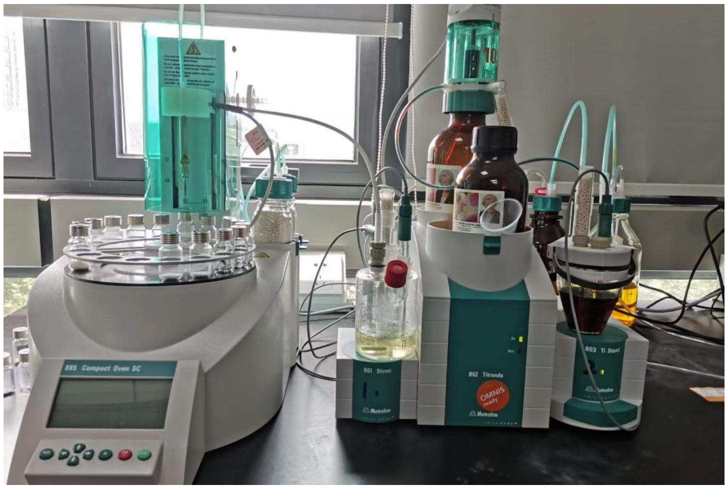

Karl Fischer coulometric titration was used to confirm the quantity of water in the Rh catalyst and substrate **1**, the analysis reports show that the mass fraction of water in the Rh catalyst is 3.59% (3.59 mg, 0.199 mmol H_2O/100 mg) and the mass fraction of water in the substrate **1** is 0.8%. Commercially obtained solvent super dry chlorobenzene shows a water content of less than 50 ppm by K. F..

Analytical Testing Center, Shanghai Institute of Organic Chemistry, Chinese Academy of Sciences, China Elemental Analysis Report

Sample number:	**S230209-1998008-001 (Work No. :1998008)**	State:	**User confirmed**
Operator:	**Feng-Jun Zhu**	Contact information:	**54925547**

Request for analysis:	Moisture Measurement-100mg-Rh(cod)2BF	Elemental content of the sample:	Rh、C、H、O、B、F
Solid, melting point:		Liquid, Boiling Point:	
Sample properties:	☑ General ☐ Photographic ☐ Absorption ☐ Volatilization ☐ Toxicity ☐ Radioactivity ☐ Explosive		
Possible molecular or structural formula :	Rh(cod)2BF4	Estimated Percentage Contained Predicted Percentage Content:	0-20%
Test Requirements:	☑ General ☐ Anhydrous and Oxygen Free ☐ Phone Sample ☐ Anhydrous and Oxygen Free for Phone Sample Delivery		
	☐ Others:		

Experimental results

Billing method:	According to the number of times (300.0 Yuan/time)	Test Fee:	300.00元

Sample weight		0.000 mg	0.000 mg	0.000 mg
Percentage content	H2O	3.59 %	0.0000 %	0.0000 %
		0.0000 %	0.0000 %	0.0000 %
		0.0000 %	0.0000 %	0.0000 %
Remarks				

File name	Type	Version Information	Operation

Sample name:	**yzq-01**	Estimated Cost:	**300元**

Analytical Testing Center, Shanghai Institute of Organic Chemistry, Chinese Academy of Sciences, China Elemental Analysis Report

Sample number:	**S230907-2020125-001 (Work No.:2020125)**	State:	**User confirmed**
Operator:	**Feng-Jun Zhu**	Contact information:	**54925547**
Sample name:	**yzq-01**	Estimated Cost:	**300元**

Request for analysis:	Moisture Measurement 300mg C18H23O2NS	Elemental content of the sample:	C,H,O,N,S
Solid, melting point:		Liquid, Boiling Point:	
Sample properties:	☑ General ☐ Photographic ☐ Absorption ☐ Volatilization ☐ Toxicity ☐ Radioactivity ☐ Explosive		
Possible molecular or structural formula :	C18H23O2NS	Estimated Percentage Contained Predicted Percentage Content:	0-20%
Test Requirements:	☑ General ☐ Anhydrous and Oxygen Free ☐ Phone Sample ☐ Anhydrous and Oxygen Free for Phone Sample Delivery		
	☐ Others:		

Experimental results

Billing method:	According to the number of times (300.0 Yuan/time)	Test Fee:	300.00元

Sample weight		0.000 mg	0.000 mg	0.000 mg
Percentage content	H2O	0.80 %	0.0000 %	0.0000 %
		0.0000 %	0.0000 %	0.0000 %
		0.0000 %	0.0000 %	0.0000 %
Remarks				

File name	Type	Version Information	Operation

7. Computational details

The geometries of compounds have been optimized at BMK/def2SVP level. The subsequent frequency calculations on the stationary points were carried out at the same level of theory to ascertain the nature of the stationary points as minima on the respective potential energy surfaces. Thermochemical corrections to 298.15 K have been calculated for all minima from unscaled vibrational frequencies obtained at this same level. All transition states were characterized by only one imaginary frequency pertaining to the desired reaction coordinate. The intrinsic reaction coordinate (IRC) calculations were carried out at the same level of theory to further authenticate the transition states. The thermochemical corrections calculated at BMK/def2SVP level have been combined with single-point energies calculated at the BMK/def2-TZVPP, SMD(PhCl) level to yield free energy G_{298} at 298.15 K. The solvent effect was estimated by the IEFPCM method with radii and nonelectrostatic terms for SMD salvation model in chlorobenzene (ε = 5.6968). Solution-phase single point energy calculations were performed based on the gas phase optimized structures. All quantum mechanical calculations have been performed with Gaussian 16. The Gibbs free energies in ether (ΔG) obtained at the BMK/def2-TZVPP, SMD(PhCl)//BMK/def2SVP level were discussed throughout this paper unless otherwise specified. The 3D images of the calculated structure were prepared using CYLView.

Table S5. The total energies, enthalpies and free energies of all species in PhCl, ε = 5.6968[a]

Sub	E_{tot}	H_{298}	G_{298}
**Int1**	-3790.652896	-3789.583710	-3789.749998
**Ts1**	-3790.630900	-3789.561113	-3789.713293
**Int2**	-3790.694469	-3789.623918	-3789.782648
**Ts2**	-3790.647558	-3789.579001	-3789.735686
**Int3**	-3790.727957	-3789.655756	-3789.813116
**Int4**	-3790.690286	-3789.617415	-3789.768498
**Ts3**	-3790.686645	-3789.615914	-3789.767364
**Pro1**	-3790.747545	-3789.673032	-3789.825771
**2**	-1302.786141	-1302.38385	-1302.457064
**Ts4**	-3790.605248	-3789.538493	-3789.701382
**Int5**	-3790.668798	-3789.600590	-3789.765690
**Ts5**	-3790.560830	-3789.495320	-3789.656720

**Int6**	-3790.672997	-3789.604040	-3789.770108
**Ts6**	-3790.641215	-3789.570851	-3789.725899
**Int7**	-3790.706982	-3789.634604	-3789.793091
**Ts7**	-3790.671739	-3789.601094	-3789.755920
**Pro2**	-3790.745303	-3789.67102	-3789.826546
**3**	-1302.790578	-1302.388538	-1302.463299
**Ts8**	-3790.598327	-3789.532068	-3789.685157
**RhL^+**	-2487.945462	-2487.278097	-2487.388927
**Int8**	-3867.148006	-3866.044973	-3866.202302
**Ts9**	-3867.119908	-3866.024985	-3866.183334
**Int9**	-3867.135667	-3866.032601	-3866.191314
**Ts10**	-3867.111628	-3866.01596	-3866.169887
**Int10**	-3867.140224	-3866.03949	-3866.201002
**Int11**	-3790.695633	-3789.621234	-3789.778167
**Ts11**	-3790.66891	-3789.598089	-3789.74916
**Int12**	-3867.118392	-3866.019706	-3866.18701
**Ts12**	-3867.086254	-3865.994203	-3866.156269
**Int13**	-3867.116359	-3866.018079	-3866.185152
**Int14**	-3790.672078	-3789.60248	-3789.767083
**Int15**	-3867.181337	-3866.078736	-3866.239653
**Ts13**	-3867.133982	-3866.042283	-3866.204356
**Int16**	-3867.160489	-3866.057764	-3866.215941
**Ts14**	-3867.138561	-3866.042439	-3866.202513
**Int17**	-3867.175873	-3866.073048	-3866.236021
**H_2O**			

a. Calculated at BMK/def2-TZVPP, SMD(PhCl)//BMK/def2-SVP level

8. Cartesian coordinates and energies of all optimized structures

Int1

```
Opt @ BMK/def2SVP in PhCl
SCF Done: E(BMK) = -3790.652896a.u.
Zero-point correction = 1.006001Hartree/Particle
Sum of electronic and thermal Free Energies = -3786.298339a.u.
-----------------------------------------------------------------
N 5.27534600,0.50889200,0.46439300
 C 5.59485400,1.92825400,0.35991500
 H 6.52248300,2.10310300,0.93052200
 H 5.80063700,2.19150700,-0.69484300
 C 4.53154000,2.84974600,0.93365600
 C 4.24921200,-0.04527400,-0.41054900
 H 4.54205400,-1.06914900,-0.69726600
 H 4.20737300, 0.56655200,-1.32738800
 C 2.86107800,-0.12460200,0.22787700
 C 1.83336100,0.49784000,-0.35340000
 C 1.31703900,1.19953500,-1.43365400
 C 1.30701400,2.65493600,-1.91601900
 C 1.38002300,1.45955100,-2.90558700
 H 2.20756600,3.24400500,-1.70771400
 H 0.36774600,3.22551000,-1.90855800
 H 0.48603200,1.28373500,-3.51642500
 H 2.32846200,1.25879400,-3.41769400
 C 4.11281600,3.91788400,0.23828500
 S 6.33883200,-0.48318700,1.25283900
 O 6.70150800,0.18831600,2.48444600
 O 5.73240000,-1.80088400,1.24714700
 C 7.82438900,-0.56330300,0.23595700
 C 7.89150200,-1.49745300,-0.80427300
 C 8.89357800,0.30231400,0.50040700
 C 9.03546500,-1.53805400,-1.60792000
 H 7.07027300,-2.20214400,-0.96020400
 C 10.02822100,0.24671800,-0.31355400
 H 8.84295000,0.98615500,1.35208500
 C 10.11593900,-0.66586500,-1.38216900
 H 9.09722500,-2.27305400,-2.41667100
 H 10.86969900,0.91568200,-0.10565200
 C 11.34715600,-0.70135700,-2.25909300
 H 11.35980900,-1.59598800,-2.90001500
 H 11.38332400,0.18633400,-2.91486300
 H 12.26653000,-0.69817300,-1.65088000
 H 3.40929600,4.63908900,0.66731200
 H 4.49029400,4.12499900,-0.76963000
 C 4.06762400,2.52644500,2.33208400
 H 3.42127500,1.63209800,2.32143300
 H 4.92806800,2.28441100,2.97986500
 H 3.50300100,3.36297200,2.77140000
 C 2.74838300,-0.97329300,1.46879500
 H 3.04682000,-2.01232200,1.24515900
 H 3.44834600,-0.62559600,2.24790600
 H 1.72611400,-0.96795700,1.87285600
 Rh -0.20342300,0.91223500,-0.14573200
 C 0.31903600,-2.34569300,-1.09069600
 C 0.81784900,-1.98065800,-2.36019600
```

C 0.86025400,-3.47762800,-0.45402900
C 1.79546200,-2.75108000,-2.99280200
H 0.44856500,-1.07734400,-2.85198900
C 1.85069100,-4.24245800,-1.08664800
H 0.52897200,-3.76494000,0.54550500
C 2.31351100,-3.88894400,-2.35710100
H 2.16131800,-2.45854200,-3.98116300
H 2.26182300,-5.11709500,-0.57478700
H 3.08291500,-4.49106600,-2.84897200
C -1.20653600,-1.97495800,1.35460400
C -1.75968200,-3.26461100,1.48289600
C -0.90163800,-1.24190400,2.51632800
C -1.98954800,-3.81133900,2.74852600
H -2.02186700,-3.84087200,0.58952600
C -1.14470900,-1.78576400,3.78261100
H -0.47629000,-0.23568700,2.43292800
C -1.68579700,-3.07191100,3.89944900
H -2.41890300,-4.81319000,2.83662200
H -0.90673000,-1.20483200,4.67786000
H -1.87081000,-3.49996000,4.88875100
C -2.34777000,3.37585200,0.90104700
C -3.13181100,4.49442000,0.56076100
C -1.34918600,3.52093500,1.88964600
C -2.93360000,5.72026100,1.20870100
H -3.89234800,4.41613200,-0.21995400
C -1.15896300,4.74417500,2.54097800
H -0.71713200,2.66643900,2.16259400
C -1.95326000,5.84705100,2.20029500
H -3.54895500,6.58147100,0.93299500
H -0.38599100,4.83748900,3.30896400
H -1.80228800,6.80686200,2.70275400
C -3.44128600,2.01854000,-1.42529400
C -2.78803200,1.93724200,-2.66891400
C -4.81962500, 2.30670400,-1.38902600
C -3.49793200,2.14833400,-3.85659200
H -1.72189300,1.68834700,-2.70085400
C -5.52606100,2.52048300,-2.57663100
H -5.34680700,2.34552300,-0.43003500
C -4.86648100,2.44113000,-3.81083800
H -2.98305700, 2.07990200,-4.81893500
H -6.59685600,2.73967200,-2.53965900
H -5.42252800,2.60438400,-4.73849600
C -3.50540900,0.70455300,1.18546500
C -3.89482800,-0.57253600,0.76511200
C -3.74445200,1.11879500,2.53174000
C -4.54575600,-1.45943400,1.69601800
C -4.34317600,0.27117900,3.43589400
H -3.45116000,2.12199300,2.84875200
C -4.75033300,-1.03610400,3.04960100
C -4.99324100,-2.76556600,1.32442400
H -4.52148000,0.60214800,4.46380900
C -5.37284900,-1.92115100,3.97968500
C -5.60223000,-3.59587700,2.24286100
H -4.85215500,-3.11100200,0.29827000
C -5.79229400,-3.17459100,3.58729100
H -5.52053500,-1.58092700,5.00941300

H -5.94280800,-4.58848300,1.93412300
H -6.27709000-3.84340200,4.30416400
C -2.48881000,-1.44086200,-1.20122200
C -3.71980200,-1.03394100,-0.66094600
C -2.43527400,-1.89709700,-2.55660600
C -4.90598000,-1.06675800,-1.48512900
C -3.55567500,-1.91632000,-3.35373600
H -1.49413900,-2.25860300,-2.96993500
C -4.81506900,-1.49393000,-2.84903800
C -6.19128700,-0.67462200,-0.99336200
H -3.48670300,-2.27328300,-4.38600100
C -5.98150600,-1.50681500,-3.67026200
C -7.30489800,-0.70455300,-1.80747300
H -6.29561700,-0.34549100,0.04181000
C -7.20355500,-1.12158400,-3.16253500
H -5.88745900,-1.83726400,-4.70933400
H -8.27691700,-0.40467900,-1.40525700
H -8.09554600,-1.14099800,-3.79514000
P -0.90596700,-1.26648600,-0.28532900
P -2.49048600,1.75352100,0.09107800

Ts1

Opt @ BMK/def2SVP in PhCl
SCF Done: E(BMK) = -3790.6309a.u.
Zero-point correction = 1.009319Hartree/Particle
Sum of electronic and thermal Free Energies = -3786.282223a.u.
Imaginary Frequency is -225.92 cm^{-1}

C 3.70697600,-2.21455700,-0.61099200
H 4.33750700,-3.12436600,-0.49757900
H 4.37022900,-1.42226700,-1.00758900
C 2.64878900,-2.48024600,-1.65317100
C 1.33677600,-2.41394100,-1.40032000
C 0.07961900,-2.60610200,-2.05893800
S 4.43613200,-1.32841700,1.75499000
O 3.75503900,-0.91583500,2.96839200
O 5.40782400,-2.40447500,1.78451500
C 5.25035900,0.11622900,1.05826400
C 6.38993000,-0.05797400,0.26262700
C 4.85713200,1.38772800,1.49227800
C 7.11892700,1.06701900,-0.13224600
H 6.71949100,1.06439900, -0.00957800
C 5.60921700,2.49918900,1.10133600
H 3.99421000,1.50074700,2.15267200
C 6.75010400,2.35876200,0.28908800
H 8.01097300,0.93556300,-0.75287800
H 5.31562700,3.49387000,1.45202900
C 7.59029000,3.55899400,-0.08288700
H 7.00117500,4.48898600,-0.05987000
H 8.42370200,3.67654200,0.63263700
H 8.03186100,3.44624500,-1.08564600
Rh -0.26476100,-1.58145600,-0.35616300
C -3.84329600,-2.05245300,-0.52221100
C -3.61010100,-2.39637200,-1.86534600
C -5.05189800,-2.45995400,0.07869100
C -4.55644200,-3.12130600,-2.59949100

H -2.67187700,-2.09717300,-2.33398800
C -5.99496800,-3.19226200,-0.65198200
H -5.26349900,-2.20294900,1.11969300
C -5.75164000,-3.52371200,-1.99143400
H -4.35542300,-3.37845300,-3.64365800
H -6.92642500,-3.50382600,-0.17048800
H -6.49112300,-4.09668500,-2.55835400
C -3.02384300,-1.37042300,2.10443600
C -3.45722900,-0.36289200,2.98190900
C -2.94925300,-2.69589400,2.58761100
C -3.77055800,-0.65958900,4.31552600
H -3.57055000,0.66035700,2.62657000
C -3.27191300,-2.99268700,3.91434700
H -2.67661400,-3.51112000,1.91407800
C -3.67242200,-1.97140200,4.78787800
H -4.10056200,0.14375800,4.98041100
H -3.21608000,-4.02690200,4.26608100
H -3.91993400,-2.20343200,5.82768900
C 1.77152600,0.95554600,-1.88760600
C 2.68258700,1.86576100,-1.32420100
C 2.09731300,0.33681100,-3.11279300
C 3.89500100,2.14431200,-1.97072000
H 2.45384100,2.37222100,-0.38285600
C 3.30256600,0.62342200,-3.75922000
H 1.39375900,-0.36687100,-3.56844700
C 4.20868800,1.52630400,-3.18525500
H 4.59233000,2.85366300,-1.51840900
H 3.53205700,0.14764500,-4.71730600
H 5.15142000,1.75450500,-3.69068800
C -0.90700400,1.61435000,-2.32002000
C -1.99911000,1.05152700,-3.00151800
C -0.56192300,2.95840200,-2.56792400
C -2.74447600,1.81907500,-3.90500100
H -2.27942100,0.01239500,-2.81108900
C -1.31306000,3.72649900,-3.46090100
H 0.30122400,3.40518400,-2.06524200
C -2.40480500,3.15738900,-4.13206800
H -3.59207500,1.36892100,-4.42926600
H -1.04153700,4.77056500,-3.63997900
H -2.98555200,3.75769200,-4.83839000
C 0.06156500,1.15770600,0.50379900
C -1.09016200,1.67021000,1.11415900
C 1.19492500,0.81036500,1.31568700
C -1.05726500,2.02365200,2.51344900
C 1.19926800,1.04836200,2.67201800
H 2.05531100,0.31834000,0.85602300
C 0.09943300,1.69985400,3.29604500
C -2.14000400,2.69526600,3.16565700
H 2.05678800,0.72701300,3.27287400
C 0.12966000,2.02429900,4.68523500
C -2.07357100,3.01666700,4.50573600
H -3.02747700,2.97113600,2.59275100
C -0.93124200,2.67214600,5.27997700
H 1.01768300,1.75565100,5.26556600
H -2.90752400,3.54459000,4.97734100
H -0.89690600,2.92688700,6.34299300

C -3.17242900,0.69388200,0.05630000
C -2.38200800,1.81416100,0.35826100
C -4.43414000,0.89366400,-0.58846300
C -2.81493600,3.13125000,-0.02846600
C -4.86647200,2.14858800,-0.95160000
H -5.07145900,0.03728400,-0.81080300
C -4.06480500,3.29616500,-0.70638200
C -2.02067300,4.29410800,0.21810200
H -5.83514500,2.27084800,-1.44619600
C -4.47764200,4.59771700,-1.11905600
C -2.44414800,5.54113200,-0.19353200
H -1.06590500,4.19152800,0.73903900
C -3.68485300,5.69807700,-0.87090500
H -5.43696500,4.70594700,-1.63457100
H -1.82215000,6.41876300,0.00476100
H -4.00864700,6.69353900,-1.18793200
P -2.60368500,-1.03921000,0.35672700
P 0.11729600,0.58540700,-1.22948800
C 3.20753100,-2.96700400,-2.97168100
H 3.91617300,-2.22758500,-3.38711600
H 3.76885700,-3.91017300,-2.83537300
H 2.41724900,-3.14662400,-3.71438900
C 1.54593000,-4.71526400,0.01118200
H 1.51429100,-5.43155400,0.85307800
H 0.81862600,-5.05079200,-0.74133800
H 2.55418100,-4.73692200,-0.42370600
C -0.57024200,-3.80820000,-2.72133800
C -0.36320800,-2.49311000,-3.50381400
H 0.07199600,-4.65805500,-2.98201200
H -1.59252700,-4.08281300,-2.43863400
H 0.40539500,-2.47219800,-4.28547300
H -1.24429400,-1.88971500,-3.74959700
N 3.19337000,-1.76045500,0.67510700
C 2.24492700,-2.63949200,1.39714600
C 1.16889900,-3.35377700,0.57891900
C -0.17509400,-3.21544800,1.03096100
H -0.35237200,-2.71662900,1.99207200
H -0.86372600,-4.03651300,0.79894500
H 2.79364600,-3.44725800,1.92661600
H 1.76502900,-2.01540800,2.16361600

Int2

Opt @ BMK/def2SVP in PhCl
SCF Done: E(BMK) = -3790.694469a.u.
Zero-point correction = 1.009218Hartree/Particle
Sum of electronic and thermal Free Energies = -3786.330501a.u.

C 4.85710500,-1.77550700,-0.89662800
H 5.38736900,-2.75285400,-0.89024400
H 5.58789800,-1.01626900,-1.21961700
C 3.70315300,-1.79214500,-1.88719100
C 2.45802800,-2.12726900,-1.47412500
C 1.19571800,-1.93083100,-2.23502000
S 5.61954800,-1.24011500,1.57829800
O 4.95309900,-0.96255700,2.83412000
O 6.54748100,-2.35086400,1.46224900

C 6.47250500,0.24336300,1.03454700
C 7.71132600,0.12297800,0.39588400
C 5.92939300,1.49521200,1.34392800
C 8.40202800,1.28509000,0.03744400
H 8.12809800,-0.86951400,0.20391200
C 6.63573900,2.64442100,0.98099600
H 4.97787300,1.55861100,1.87766100
C 7.87926500,2.55925600,0.32473300
H 9.37228100,1.19962500,-0.46225300
H 6.22204200,3.62854100,1.22293400
C 8.65796200,3.80773300,-0.02298600
H 7.99103400,4.67221400,-0.16617500
H 9.36026200,4.06074300,0.79133700
H 9.25229200,3.66775200,-0.93965600
Rh -0.32751900,-1.29945100,-1.09249500
C -3.35144200,-2.92682200,-0.49880500
C -3.44217500,-3.03394200,-1.90412100
C -3.89355200,-3.95934200, 0.28787200
C -4.06038800,-4.13315200,-2.50585000
H -3.05033600,-2.23271100,-2.54134900
C -4.50434100,-5.06793500,-0.31645100
H -3.84098100,-3.90632600,1.37798200
C -4.58955100,-5.15908900,-1.70919100
H -4.12791600,-4.19166800,-3.59599600
H -4.91691700,-5.86284000,0.31146400
H -5.06718000,-6.02522700,-2.17602400
C -2.44200100,-1.86110100,1.99236200
C -3.43692400,-1.41405200,2.88016800
C -1.42368400,-2.70657200,2.47678300
C -3.39885800,-1.78276400,4.22999600
H -4.24742000,-0.77718100,2.51860700
C -1.39288300,-3.08156300,3.82423700
H -0.66190700,-3.09288700,1.79524800
C -2.37641200,-2.61296400,4.70510100
H -4.17471200,-1.42065400,4.91053900
H -0.59832600,-3.73988600,4.18662300
H -2.34842500,-2.90007900,5.76019700
C 1.09809900,1.93875900,-1.32741900
C 1.30471600,3.24614500,-0.83828200
C 1.97418500,1.42814400,-2.30288500
C 2.36280300,4.02299300,-1.32002200
H 0.64050100,3.66338300,-0.07604300
C 3.02636900,2.21406700,-2.78861400
H 1.82612200,0.41613500,-2.68790800
C 3.22287100,3.51026900,-2.30018600
H 2.51155700,5.03460800,-0.93162000
H 3.69214100,1.81111800,-3.55703500
H 4.04552900,4.12164800,-2.68187900
C -1.60322400,1.67566400,-2.08344200
C -2.22452400,0.82111700,-3.01171300
C -1.86460200,3.05725800,-2.15211400
C -3.10191000,1.32778200,-3.97782400
H -2.02372600,-0.25700700,-2.98942700
C -2.74514500,3.56437400,-3.11115700
H -1.38390400,3.74461400,-1.45064200
C -3.36550900,2.70146400,-4.02500500

H -3.57563400,0.65040700,-4.69366800
H -2.94589600,4.63870700,-3.14691000
H -4.04946200,3.10183000,-4.77877700
C -0.82676900,1.46165000,0.80133000
C -2.13701800,1.47508400,1.29170700
C 0.27020900,1.65135800,1.70601400
C -2.37256400,1.75066100,2.69096800
C 0.06000200,1.87006900,3.04559200
H 1.29369500,1.62486500,1.32871500
C -1.26004900,1.93693400,3.57305100
C -3.68810300,1.84574700,3.24414000
H 0.91176400,2.00293100,3.71963800
C -1.48677900,2.18998700,4.95807600
C -3.87900400,2.10192900,4.58677800
H -4.55632700,1.72102300,2.59461000
C -2.76887400,2.27177600,5.45801000
H -0.62188000,2.32328500,5.61518600
H -4.89527800,2.17810200,4.98415700
H -2.93529000,2.47186200,6.52026900
C -3.66698600,-0.08608200,0.00139800
C -3.33448200,1.20989400,0.41947100
C -4.87391000,-0.29279500,-0.73799200
C -4.17460300,2.32165700,0.05651400
C -5.68309500,0.76152000,-1.09553900
H -5.16928100,-1.30185200,-1.02731300
C -5.34571700,2.09463600,-0.73541200
C -3.86647400,3.66607900,0.43395700
H -6.60028000,0.57893600,-1.66415200
C -6.15272800,3.20026000,-1.13750500
C -4.66520200,4.71666300,0.03073700
H -2.98542700,3.86460600,1.04817200
C -5.81983100,4.48576900,-0.76697800
H -7.04417400,3.00725200,-1.74239800
H -4.41135100,5.73745300,0.33095000
H -6.44481100,5.32846300,-1.07607800
P -2.49166600,-1.48062100,0.21953900
P -0.39584700,0.99185500,-0.90735500
C 4.09992000,-1.35156400,-3.27670900
H 4.61588200,-0.37639600,-3.22119200
H 4.82589100,-2.06201100,-3.71442800
H 3.25761200,-1.25416600,-3.97193800
C 1.51027200,-3.82431000,0.22953000
H 1.47736300,-4.02931100,1.31397300
H 0.48369100,-3.91148400,-0.15673900
H 2.12085900,-4.61437400,-0.23883200
C 0.27011300,-3.18610900,-2.63903100
C 0.95110400,-2.28031600,-3.67231400
H 0.67436900,-4.18747000,-2.46405700
H -0.83260100,-3.15180500,-2.60107400
H 1.76520600,-2.73115600,-4.25019100
H 0.29167000,-1.63832800,-4.26942100
N 4.38347900,-1.42947900,0.44211000
C 3.38831500,-2.39450100,0.90088000
C 2.13583900,-2.42577000,-0.00562600
C 1.13734400,-1.30606400,0.34922600
H 1.68617600,-0.35136200,0.33206700

H 0.66094400,-1.43244000,1.33722600
H 3.83629800,-3.41068000,0.92118300
H 3.09190400,-2.13967100,1.92966300

Ts2

Opt @ BMK/def2SVP in PhCl
SCF Done: E(BMK) = -3790.647558a.u.
Zero-point correction = 1.007730Hartree/Particle
Sum of electronic and thermal Free Energies = -3786.295705a.u.
Imaginary Frequency is -499.37 cm^{-1}

C 4.36758300,-2.15746700,-0.12191800
H 5.02239200,-2.95582600,0.29503400
H 5.01135800,-1.46990500,-0.69333700
C 3.33389800,-2.72957100,-1.08324600
C 2.05543900,-2.94481300,-0.66561800
C 0.92641600,-3.01786600,-1.62997400
S 4.67011000,-0.55256800,2.01094300
O 3.76925800,0.35788000,2.69446800
O 5.47057800,-1.48735700,2.78151900
C 5.78142900,0.39426700,0.96883200
C 7.14082500,0.05680200,0.98317400
C 5.31285500,1.51153400,0.26998600
C 8.04229100,0.85303900,0.27263600
H 7.47745300,-0.80774300,1.56111700
C 6.23326300,2.29727000,-0.43008200
H 4.24888100,1.76901400,0.27359100
C 7.60635800,1.98834800,-0.43607800
H 9.10659800,0.59688600,0.28171100
H 5.87724200,3.17750700,-0.97308100
C 8.60086600,2.87072300,-1.15535100
H 9.11569000,3.53276600,-0.43663800
H 9.37428800,2.27062300,-1.66114500
H 8.10827500,3.50950700,-1.90432200
Rh -0.55342500,-1.62521700,-0.91738700
C -3.72434600,-2.16312900,-1.05753300
C -3.61331100,-1.91287900,-2.44337000
C -4.54230100,-3.21985900,-0.62299300
C -4.30360500,-2.70152900,-3.36928600
H -3.01153700,-1.06870100,-2.79956500
C -5.23001300,-4.01188200,-1.55481400
H -4.64919000,-3.42927500,0.44450500
C -5.11166700,-3.75880600,-2.92561000
H -4.21881700,-2.48600200,-4.43844100
H -5.86632900,-4.82847800,-1.20136900
H -5.65274700,-4.37708800,-3.64765200
C -3.07504600,-1.80591300,1.72343400
C -4.01323600,-1.18634100,2.56946300
C -2.46052400,-3.00264300,2.14495200
C -4.32369900,-1.75004800,3.81351300
H -4.51262900,-0.26661000,2.25608000
C -2.77931200,-3.56791800,3.38349600
H -1.73433600,-3.49648800,1.49335800
C -3.70838800,-2.93843700,4.22302200
H -5.05326200,-1.25768900,4.46253600
H -2.29818500,-4.49884400,3.69665400

H -3.95245700,-3.37554200,5.19555900
C 1.57319200,1.29879500,-1.70655300
C 1.98299900,2.64784600,-1.66356900
C 2.38156800,0.36283400,-2.37113100
C 3.16398400,3.04841200,-2.29321000
H 1.36662700,3.39265100,-1.15033100
C 3.56775100,0.76517900,-3.00191200
H 2.06300900,-0.68115900,-2.41114300
C 3.95438100,2.10787900, -2.97222000
H 3.46069500,4.10133000,-2.26800400
H 4.18027000,0.02904300,-3.53096300
H 4.87103600,2.42462100,-3.47799300
C -1.11171100,1.70866100,-2.29506400
C -1.49644600,1.05817200,-3.48276300
C -1.42875500,3.06892700,-2.13356000
C -2.20219200,1.74467200,-4.47857800
H -1.21616500,0.01090800,-3.64542500
C -2.14356800,3.75305900,-3.12240500
H -1.12501100,3.59820000,-1.22676000
C -2.53395700,3.09242900,-4.29486000
H -2.48869600,1.22843300,-5.39930600
H -2.39365600,4.80776600,-2.97564100
H -3.08927100,3.63040000,-5.06854200
C -0.24540200,1.47463900,0.56176900
C -1.47859300,1.61009600,1.21761900
C 0.96684500,1.56648400,1.32114300
C -1.50552900,1.83062700,2.64592800
C 0.95535400,1.77882100,2.67950500
H 1.93224200,1.41086400,0.83368000
C -0.27517500,1.91038200,3.37737700
C -2.72641400,1.97695200,3.37864200
H 1.90394400   1.80366900   3.22340100
C -0.29904500,2.11637700,4.78939200
C -2.71918200,2.17753000,4.74379800
H -3.68070300,1.93768500,2.85120500
C -1.49417600,2.24751300,5.46226600
H 0.65420900,2.16920000,5.32418100
H -3.66657300,2.28796400,5.27917100
H -1.50520000,2.40779300,6.54402900
C -3.44341400,0.50487200,-0.00945100
C -2.80016200,1.64297700,0.49094900
C -4.73418900,0.63366200,-0.61930800
C -3.43634500,2.93512500,0.35847900
C -5.34720500,1.85634000,-0.74967500
H -5.25041800,-0.25467300,-0.98540800
C -4.71352300,3.03919900,-0.27937300
C -2.82384800,4.14184300,0.82438600
H -6.33363700,1.92868300,-1.21808600
C -5.33136400,4.31605800,-0.43236300
C -3.44063100,5.36523900,0.65834700
H -1.85074100,4.09693600,1.31692000
C -4.71016600,5.45828000,0.02469400
H -6.30942400,4.36966200,-0.92054200
H -2.95015900,6.27227800,1.02325700
H -5.18981600,6.43399600,-0.09432600
P -2.69027900,-1.16379700,0.07133900

P -0.07484000,0.78195200,-1.11989700
C 3.83165100,-2.90624300,-2.50279000
H 4.72910600,-2.29241500,-2.68047700
H 4.11737700,-3.95337200,-2.70855500
H 3.07433700,-2.62465500,-3.25680500
C 1.19103900,-4.11936400,1.44789600
H 1.12545400,-3.97749300, 2.54075900
H 0.20824500,-4.47484800,1.11815000
H 1.93090000,-4.91431900,1.25584500
C -0.53672200,-3.61106200,-1.24667700
C 0.94221600,-3.75636500,-2.79805700
N 3.69543900,-1.41039900,0.93428200
C 2.81200400,-2.30701500, 1.67326800
C 1.62970500,-2.78342200,0.79620400
C 0.52078100,-1.71205800,0.82302500
H 1.01341600,-0.73896300,0.92211900
H -0.15499400,-1.84750300,1.68364300
H 3.39192900,-3.17962300,2.03654400
H 2.41116200,-1.78124600,2.55441200
H 0.17905000,-3.63398900,-3.57152600
H 1.71610100,-4.50974400,-2.99910700
H -1.24381400,-3.77754200,-2.07613300
H -0.47029600,-4.50992300,-0.62584200

Int3

Opt @ BMK/def2SVP in PhCl
SCF Done: E(BMK) = -3790.727957a.u.
Zero-point correction = 1.011430Hartree/Particle
Sum of electronic and thermal Free Energies = -3786.375298a.u.

C 4.80213400,-2.17529100,-0.57097300
H 5.22585600,-3.16446700,-0.29327600
H 5.63014600,-1.57541900,-0.98541800
C 3.73098400,-2.30652900,-1.63643500
C 2.42914200,-2.34636300,-1.28812600
C 1.33465800,-2.11498800,-2.28644800
S 5.39433700,-1.11809500,1.78469400
O 4.64612500,-0.51163200,2.86659300
O 6.23007700,-2.28101300,2.02093300
C 6.40990200,0.14059700,1.00520200
C 7.68542900,-0.19973900,0.54096000
C 5.94530000,1.45958000,0.96207400
C 8.49572400,0.80190400,-0.00247400
H 8.03603500,-1.23207700,0.62426700
C 6.77233100,2.44756300,0.42003500
H 4.95833600,1.70507700,1.36381600
C 8.05630000,2.13725400,-0.06915500
H 9.49453200,0.54357700,-0.36859900
H 6.42183800,3.48440800,0.39181200
C 8.96308900,3.21774300,-0.61269400
H 8.38942300,4.09246600,-0.95587000
H 9.66052500,3.56235800,0.17150700
H 9.57018300,2.84540500,-1.45308200
Rh -0.22237900,-0.89561600,-1.35164100
C -3.03039600,-2.31942100,-1.81001600
C -3.16203600,-1.59835900,-3.01824600

C -3.17467400,-3.71821500,-1.83593700
C -3.43762000,-2.26372500,-4.21828800
H -3.07233700,-0.50575200,-3.01954800
C -3.45013600,-4.38016500,-3.04038900
H -3.06669100,-4.29947400,-0.91591500
C -3.57971500,-3.65820300,-4.23280000
H -3.54908300,-1.69068100,-5.14332600
H -3.56481900,-5.46802000,-3.04344300
H -3.79642600,-4.17893200,-5.16986900
C -2.82728300,-2.48437400,1.08822700
C -4.14712200,-2.96277000,1.24226300
C -1.88804300,-2.74151800,2.10087500
C -4.50475200,-3.69984200,2.37355600
H -4.89997600,-2.75795600,0.47434800
C -2.25091100,-3.47547400,3.23869700
H -0.86989500,-2.36229000,2.01349500
C -3.55553200,-3.95829100,3.37446000
H -5.52842900,-4.07097800,2.47759200
H -1.50938800,-3.66358100,4.01992000
H -3.83847100,-4.53342600,4.26092800
C 1.04915700,2.52911900,-1.01683200
C 0.89600200,3.92929000,-1.09335100
C 2.34710400,1.99043500,-1.12371800
C 2.00347500,4.76294700,-1.27830200
H -0.09586900,4.38116400,-1.02352200
C 3.45472200,2.82708000,-1.31527600
H 2.51435200,0.91404000,-1.05283600
C 3.28609500,4.21369500,-1.39651600
H 1.85950200,5.84541100,-1.33975000
H 4.45192500,2.38637900,-1.40280600
H 4.14963300,4.86588900,-1.55617600
C -1.68421500,2.32432500,-1.76327100
C -1.83773400,1.95110400,-3.11329700
C -2.44877500,3.39275000,-1.26160600
C -2.74639500,2.62262100,-3.93983800
H -1.22805100,1.14089000,-3.52911300
C -3.36490500,4.05674600,-2.08505100
H -2.33503500,3.70766400,-0.22103000
C -3.51673700,3.67215500,-3.42355800
H -2.84943900,2.32892500,-4.98836000
H -3.95994400,4.87871800,-1.67680400
H -4.23083200,4.19494500,-4.06636500
C -0.79564200,1.52065900,0.99753200
C -2.00441600,1.06370900,1.54532200
C 0.31874300,1.75621600,1.86543000
C -2.07790700,0.74556400,2.95045000
C 0.24812200,1.49600600,3.21499100
H 1.25142800,2.14428600,1.45200000
C -0.93470300,0.95513000,3.78908800
C -3.26053200,0.20167100,3.54520700
H 1.11550600,1.68608800,3.85464200
C -1.00493600,0.62649900,5.17625400
C -3.29567300,-0.11528400,4.88699300
H -4.14051200,0.01827200,2.92448800
C -2.15970900,0.10236800,5.71576100
H -0.12310000,0.79796200,5.80134100

H -4.20682100,-0.53834300,5.31921300
H -2.20678700,-0.14741700,6.77954600
C -3.58912900,-0.04848600,-0.12271000
C -3.28720900,0.98956500,0.75878000
C -4.85702700,-0.06936300,-0.78825400
C -4.25409900,2.03876800,0.98793900
C -5.78006000,0.92950800,-0.59136100
H -5.10207300,-0.89308300,-1.46078500
C -5.50509100,2.01039800,0.29274300
C -4.00382500,3.13398900,1.87344500
H -6.74311100,0.89754200,-1.11035200
C -6.45357200,3.05597400,0.49616200
C -4.93798800,4.13563800,2.04770000
H -3.05877800,3.18198700,2.41847500
C -6.17856700,4.09948100,1.35441800
H -7.40549900,3.01296000,-0.04223300
H -4.72370100,4.96275100,2.73061600
H -6.91133900,4.89687900,1.50743500
P -2.41398900,-1.43623900,-0.34046100
P -0.41896900,1.45641300,-0.79334500
C 4.23949900,-2.33789400,-3.05723200
H 4.73462900,-1.38570800,-3.32074200
H 5.00253000,-3.13043200,-3.16124300
H 3.44417900,-2.53714400,-3.78947300
C 1.19444400,-3.64037800,0.57464600
H 1.16505800,-3.70059800,1.67678500
H 0.15402100,-3.67417400,0.22832900
H 1.72512300, -4.53465300,0.20795900
C 1.25598200,-0.82189100,-2.90044900
C 0.12933700,-2.86159700,-2.29236200
H 0.06228200,-3.81418600,-1.76648000
H -0.54072600,-2.76423400,-3.15758200
N 4.25148400,-1.48866700,0.59669200
C 3.13172500,-2.24900200,1.14646600
C 1.93637700,-2.34234800,0.16030100
C 1.01952900,-1.10532400,0.28477600
H 1.65020800,-0.20638600,0.35121900
H 0.40299000,-1.13995300,1.19578900
H 3.46983900,-3.27635300,1.39486400
H 2.79619100,-1.77140100,2.07908800
H 0.63726600,-0.71633000,-3.80571200
H 2.11617900,-0.15044100,-2.85568400

Int4
Opt @ BMK/def2SVP in PhCl
SCF Done: E(BMK) = -3790.690286a.u.
Zero-point correction = 1.012834Hartree/Particle
Sum of electronic and thermal Free Energies = -3786.332162a.u.

C 3.31021900,-2.29894900,-0.62123000
H 4.13739500,-3.04575800,-0.64872300
H 3.62454900,-1.43896200,-1.23787000
C 2.12198400,-2.97012500,-1.28231900
C 1.04488400,-3.42488800,-0.56743700
C -0.10859300,-4.19050400,-1.16547400
S 4.47605500,-1.42861100,1.59382100

O 4.01452300,-0.91094500,2.86763100
O 5.37767400,-2.56434600,1.53809800
C 5.27594200,-0.08104000,0.71054400
C 6.21523000,-0.38110300,-0.28462300
C 5.14189200,1.21991400,1.21074900
C 7.00255500,0.64841700,-0.80605100
H 6.35804200,-1.41424800,-0.61247400
C 5.94918800,2.23382300,0.68716300
H 4.44369000,1.42409300,2.02636100
C 6.89445600,1.96526500,-0.31991800
H 7.74126100,0.41757200,-1.58019400
H 5.86072100,3.24881300,1.08782200
C 7.80754300,3.05358700,-0.83644000
H 7.36205800,4.05230100,-0.70680100
H 8.76314000,3.04333100,-0.28221900
H 8.04536200,2.90875400,-1.90219600
Rh -0.66160200,-1.64712200,-0.70379100
C -3.91070400,-1.41232700,-1.36954400
C -3.41510200,-1.61472800,-2.67263400
C -5.27502400,-1.66722000,-1.12435800
C -4.25541100,-2.04157800,-3.70722000
H -2.35202200,-1.45870300,-2.89068900
C -6.11609900,-2.09890500,-2.15649200
H -5.68630100,-1.52851800,-0.12033600
C -5.61028600,-2.28319700,-3.44983700
H -3.84858500,-2.19462500,-4.71071700
H -7.17193100,-2.29316700,-1.94710800
H -6.26952200,-2.62236900,-4.25407100
C -3.43112000,-1.56609500,1.46192400
C -3.45857700,-0.82825000,2.65718800
C -3.86815000,-2.90921000,1.48337500
C -3.89992500,-1.41599200,3.84989300
H -3.13886100,0.21279200,2.66845000
C -4.31492000,-3.49034300,2.67368700
H -3.88046900,-3.50160900,0.56619900
C -4.32676200,-2.74736600,3.86270500
H -3.91024800,-0.82227100,4.76825500
H -4.65938900,-4.52848200,2.67054900
H -4.67498600,-3.20571100,4.79280100
C 1.91097600,0.91476900,-1.78394400
C 2.85387000,1.89522800,-1.42111200
C 2.14300500,0.15047700,-2.94378900
C 3.99264000,2.10399800,-2.20678400
H 2.69954900,2.51096600,-0.53043300
C 3.28458400,0.35702700,-3.72664500
H 1.41082600,-0.60406800,-3.25062400
C 4.21242800,1.33806900,-3.35857700
H 4.70836100,2.87784000,-1.91985300
H 3.44141600,-0.23797900,-4.63146700
H 5.10048900,1.51234600,-3.97268400
C -0.60487300,2.02591800,-2.00529500
C -1.62980100,1.70379800,-2.90905900
C -0.18435300,3.36720800,-1.90665500
C -2.22908000,2.69760500,-3.69441200
H -1.98124600,0.67277200,-2.99571900
C -0.78545100,4.36026400,-2.68357300

H 0.61320100,3.64216000,-1.21019000
C -1.80893600,4.02688900,-3.58206500
H -3.02692400,2.42828500,-4.39218300
H -0.45405500,5.39809400,-2.58809000
H -2.27543300,4.80400400,-4.19434000
C 0.28733700,1.25155600,0.68619000
C -0.89741100,1.59574100,1.34835500
C 1.47967200,1.01370900,1.43751100
C -0.87899100,1.78163100,2.78176100
C 1.49968500,1.13822700,2.80708100
H 2.37500200,0.67023000,0.92069600
C 0.32983800,1.53469600,3.51267700
C -2.02352600,2.22503900,3.51931300
H 2.41389100,0.89907300,3.36016600
C 0.34900400,1.69661600,4.93057400
C -1.97210200,2.38475900,4.88933700
H -2.95198600,2.45334600,2.99158700
C -0.77626300,2.11187200,5.60884400
H 1.28073400,1.49060100,5.46638800
H -2.85951800,2.73213200,5.42642800
H -0.75177300,2.24011200,6.69460500
C -3.05504700,0.98120000,0.04925100
C -2.17022700,1.92211600,0.60426900
C -4.26015800,1.45220400,-0.57011500
C -2.46294100,3.33530600,0.48106500
C -4.55301400,2.78907200,-0.67764300
H -4.96728300,0.73977400,-0.99040800
C -3.65450200,3.76933200,-0.18102800
C -1.57292100,4.34373600,0.97086900
H -5.48018700,3.10812900,-1.16348900
C -3.92036900,5.16147600,-0.33844200
C -1.85198100,5.68482700,0.80415800
H -0.65317900,4.05230400,1.47996300
C -3.03753900,6.10418900,0.14239400
H -4.84020700,5.46497800,-0.84772300
H -1.15322800,6.43371400,1.18807500
H -3.24681900,7.17084400,0.02115200
P -2.76337500,-0.84222100,-0.06735400
P 0.25619100,0.72377500,-1.05229900
C 2.35917000,-3.24750100,-2.74958300
H 2.98929300,-2.46423300,-3.19751000
H 2.90188600,-4.20365400,-2.86455300
H 1.42224700,-3.33844600,-3.31850400
C 0.56583200,-4.52760500,1.68633900
H 0.62394000,-4.37839300,2.77795500
H -0.45641700,-4.84743200,1.44626800
H 1.25169400,-5.34643400,1.41300400
C 0.01705000,-5.21241300,-2.03129400
C -1.40082700,-3.56763500,-0.72302400
N 3.05650100,-1.84345700,0.73945600
C 2.35334500,-2.86733600,1.52686900
C 0.96445500,-3.21686600,0.96978700
C -0.06549500,-2.08554000,1.19231300
H 0.39666100,-1.18034900,1.61133000
H -0.90973300,-2.40186900,1.82300300
H 2.96396400,-3.79251700,1.56064500

H 2.24401900,-2.49614000,2.55639600
H -2.21572700,-3.71983800,-1.45077700
H -1.73197700,-3.92366200,0.26232400
H 0.99324300,-5.60330500,-2.33068600
H -0.87058800,-5.71218600,-2.43296900

Ts3

Opt @ BMK/def2SVP in PhCl
SCF Done: E(BMK) = -3790.67818a.u.
Zero-point correction = 1.011038Hartree/Particle
Sum of electronic and thermal Free Energies = -3786.328241a.u.
Imaginary Frequency is -471.52 cm^{-1}

C -4.95255900,0.88631700,-1.64727500
H -5.80920100,1.33116500,-2.18398400
H -5.09893200,-0.21630400,-1.68867700
C -3.67699200,1.18001800,-2.40230400
C -2.70058400,1.93029400,-1.85066400
C -1.34404100,2.17722300,-2.38586500
S -6.31603100,1.06912600,0.62126200
O -6.17497700,1.82117500,1.84835800
O -7.43607800,1.23714400,-0.27679000
C -6.21724000,-0.68339100,1.03611100
C -6.85655400,-1.62423500,0.21957200
C -5.48277400,-1.08249900,2.16056500
C -6.73662300,-2.98442100,0.52728400
H -7.46125800,-1.28561800,-0.62638200
C -5.37245100,-2.44589600,2.45087100
H -5.03289800,-0.32797800,2.81229300
C -5.99544000,-3.41703900,1.64277200
H -7.24532000,-3.72305900,-0.10071600
H -4.80944900,-2.76336100,3.33461000
C -5.91492700,-4.88444500,1.99779400
H -4.96647400,-5.12486100,2.50331000
H -6.73560900,-5.15707700,2.68513200
H -6.00679400,-5.52103000,1.10406600
Rh 0.14959300,1.40844100,-0.96115600
C -0.84601400,-1.75935400,-1.56581400
C -1.95091600,-1.55982700,-0.71145100
C -1.01769300,-2.56827000,-2.70440700
C -3.17893400,-2.18044900,-0.95594000
H -1.84024500,-0.92264200,0.17112700
C -2.25569800,-3.17636800,-2.96333000
H -0.18745200,-2.72721000,-3.39675400
C -3.33281100,-2.99344500,-2.08897000
H -4.01470100,-2.03166600,-0.26336300
H -2.37075200,-3.80312800,-3.85252900
H -4.29201300, -3.48045700,-2.28750900
C 1.88477900,-1.39470200,-2.45595600
C 2.25693700,-2.74798600,-2.59455500
C 2.45818200,-0.43791600,-3.31031800
C 3.16888500,-3.13242500,-3.58087700
H 1.83974000,-3.50311700,-1.91999400
C 3.37377100,-0.82449000,-4.29733500
H 2.20917900,0.61802500, -3.17838700
C 3.72828400,-2.17068300,-4.43507900

```
H 3.45157000,-4.18436300,-3.67919700
H 3.81751700,-0.06941700,-4.95241900
H 4.44532200,-2.47313700,-5.20366100
C 2.48040600,3.38279500,0.94069400
C 3.78708700,3.62076300,1.42378900
C 1.63677900,4.48975600,0.73929600
C 4.22144800,4.91835500,1.70709500
H 4.48464700,2.78923400,1.55595500
C 2.07115800,5.79168200,1.02445500
H 0.62853500,4.34114600,0.35417000
C 3.36269000,6.00917600,1.51227100
H 5.23813700,5.07895500,2.07719300
H 1.39531800,6.63584300,0.86095600
H 3.70438000,7.02428300,1.73397200
C 1.57141300,1.06902800,2.35361800
C 0.35019000,0.43699800,2.65529000
C 2.51386900,1.23707400,3.38693800
C 0.07613000,-0.01593500,3.95046400
H -0.38319600,0.27231300,1.86187000
C 2.24418800,0.77768100,4.67939800
H 3.46977900,1.72692000,3.19158100
C 1.02427100,0.15159400,4.96571100
H -0.87847600,-0.50483900,4.16370100
H 2.99040400,0.91281600,5.46724700
H 0.81298900,-0.20053500,5.97941100
C 3.32725200,0.82942800,-0.04030000
C 3.54427300,-0.54222100,0.09775200
C 4.17788700,1.59303800,-0.90613600
C 4.67098700,-1.16478000,-0.55908800
C 5.24690100,1.01380300,-1.54665200
H 3.98226100,2.65692000,-1.05558100
C 5.53776000,-0.36971300,-1.37621000
C 4.96091600,-2.55836700,-0.43235500
H 5.89111000,1.61711600,-2.19404400
C 6.65940500,-0.97299300,-2.01572600
C 6.05804600,-3.11567700,-1.05891300
H 4.30903100,-3.19351100,0.16976200
C 6.92045100,-2.31820100,-1.85732400
H 7.31227900,-0.34686300,-2.63186100
H 6.26478500,-4.18309000,-0.93949600
H 7.78759000,-2.77306100,-2.34455300
C 1.33773500,-1.74059200,0.34858400
C 2.59782700,-1.41847200,0.86975800
C 0.50647000,-2.66086000,1.05959000
C 3.01864900,-1.95791400,2.13533000
C 0.89932600,-3.19180900,2.26820500
H -0.45337100,-2.96340500,0.64008100
C 2.14725000,-2.84026100,2.85122000
C 4.28067100,-1.63064600,2.72206400
H 0.24804000,-3.89853300,2.79213500
C 2.55122400,-3.35632500,4.11833500
C 4.64580200,-2.14573100,3.94877500
H 4.96212400,-0.96200300,2.19129600
C 3.77342200,-3.01649100,4.65823100
H 1.87414100,-4.03079300,4.65176000
H 5.61607100,-1.88376500,4.38049000
```

H 4.07830200,-3.41863100,5.62860900
P 0.71270300,-0.89000500,-1.16339900
P 1.86235100,1.68288100,0.65817300
C -3.62753700,0.53065100,-3.76437100
H -3.31217200,-0.52639100,-3.67634000
H -4.63104800,0.53151100,-4.22245100
H -2.93733500,1.04576600,-4.44751200
C -3.56266000,3.95137100,-0.53791300
H -3.54805000,4.43837500,0.45211600
H -3.10784100,4.63468200,-1.27131700
H -4.61416100,3.81027500,-0.82834700
C -0.56352200,1.28562200,-3.12543300
C -0.66145900,3.28948700,-1.69766400
N -4.95831600,1.37441400,-0.27505200
C -3.69754600,1.62598500,0.39860300
C -2.84825700,2.58105300,-0.47006300
C -1.41456100,2.70445800,0.10351600
H -3.92030200,2.08710300,1.37189000
H -3.13092400,0.68862400,0.57093400
H -1.22676900,3.64272800,0.63844200
H -1.20588100,1.87519800,0.80471500
H 0.31807700,1.64190700,-3.66442300
H -0.98894900,0.33483200,-3.45584200
H -1.18924500,4.22565400,-1.50978000
H 0.37356500,3.49908100,-2.02462700

Pro1
Opt @ BMK/def2SVP in PhCl
SCF Done: E(BMK) = -3790.747545a.u.
Zero-point correction = 1.014679Hartree/Particle
Sum of electronic and thermal Free Energies = -3786.402604a.u.

C 3.43858200,-1.39716000,-1.06476900
H 4.14117700,-1.97355300,-1.69297500
H 3.32349800,-0.41330400,-1.55648500
C 2.11218900,-2.14672200,-1.16006400
C 1.68026700,-2.84186600,-0.03061800
C 0.40636800,-3.52228800,0.14156400
S 5.66775300,-1.51201800,0.40044500
O 5.92378000,-1.86627300,1.78125300
O 6.07403400,-2.37827300,-0.69133900
C 6.42138400,0.09170500,0.09712000
C 6.65258700,0.50616600,-1.22056800
C 6.80564900,0.88198200,1.18458400
C 7.24858400,1.75068500,-1.44408800
H 6.39545600,-0.14967000,-2.05695700
C 7.41522200,2.11763000,0.94113900
H 6.64522900,0.52080200,2.20358100
C 7.64351700,2.57348000,-0.37039400
H 7.43447500,2.07992700,-2.47156300
H 7.73359600,2.73362900,1.78842800
C 8.34354800,3.88880100,-0.62673300
H 8.23088200,4.57852000,0.22389500
H 9.42478200,3.72267100,-0.77992800
H 7.95397300,4.38159500,-1.53156900
Rh -0.08724600,-1.49779400,-0.48074500

C -2.96293400,-2.17554900,-2.18845100
C -2.27509400,-1.95998300,-3.40267900
C -3.90748300,-3.21484800,-2.12975700
C -2.53873800,-2.74444600,-4.52831700
H -1.53209400,-1.15840500,-3.47123500
C -4.16424200,-4.00947600,-3.25774300
H -4.44290800,-3.42301500,-1.20067100
C -3.48635900,-3.77659600,-4.45756300
H -2.00235100,-2.55323000,-5.46228800
H -4.90021800,-4.81585100,-3.19047700
H -3.69045000,-4.39645000,-5.33527200
C -3.53327600,-1.73303400,0.62900900
C -4.93822200,-1.63653100,0.55469500
C -2.94202600,-2.21404700,1.80785300
C -5.72998600,-2.04497600,1.63112900
H -5.41425700,-1.23167600,-0.34448300
C -3.73654600,-2.61792100,2.88867600
H -1.85242400,-2.22693500,1.88858600
C -5.13020400,-2.53882900,2.79929800
H -6.81904100,-1.96939300,1.56372700
H -3.26447000,-2.98243400,3.80554600
H -5.75247500,-2.85200900,3.64254600
C 1.56208000,1.73055900,0.79064100
C 1.58209200,2.44478100,2.00412000
C 2.70337900,1.78863800,-0.03459100
C 2.72788400,3.15310200,2.39420900
H 0.70479100,2.47703400,2.65185000
C 3.84501400,2.49256500,0.35295700
H 2.68194500,1.31138000,-1.01687400
C 3.86363300,3.16992500,1.57920800
H 2.72183100,3.70119600,3.34091600
H 4.71981300,2.51532600,-0.30348700
H 4.75743500,3.71963500,1.88789600
C -0.25962400,2.06942400,-1.24428000
C -0.34416000,1.63005200,-2.57590600
C -0.32855100,3.45157600,-0.97738300
C -0.51711000,2.54707700,-3.62158500
H -0.26449600,0.55963500,-2.79454200
C -0.51180800,4.36560100,-2.01723300
H -0.23408600,3.81567200,0.05022900
C -0.60656900,3.91482200,-3.34211500
H -0.57733600,2.19121800,-4.65402200
H -0.57410200,5.43454700,-1.79464000
H -0.73979900,4.63292600,-4.15644200
C -1.16467900,0.95928100,1.40478100
C -2.51346100,1.25161400,1.20207300
C -0.73267100,0.50956000,2.69890000
C -3.43746300,1.20540600,2.31450400
C -1.59525700,0.46309100,3.76638900
H 0.30869800,0.21023400,2.84368000
C -2.96225100,0.83611200,3.61354400
C -4.82546100,1.50868100,2.16958300
H -1.23844600,0.13668900,4.74839400
C -3.86150100,0.81476200,4.71777500
C -5.67596700,1.48187400,3.25792700
H -5.21919300,1.77075200,1.18593700

C -5.19229300,1.13795800,4.54760900
H -3.47665200,0.53783500,5.70429100
H -6.73326800,1.72792400,3.12428200
H -5.87623400,1.12653100,5.40101100
C -3.17255700,0.50379800,-1.11039800
C -3.05972400,1.53407000,-0.16902700
C -3.76703500,0.77871800,-2.38062500
C -3.48377900,2.86608300,-0.50160200
C -4.18140100,2.04937100,-2.71167900
H -3.90315100,-0.02373700,-3.10634300
C -4.02899200,3.12881700,-1.79918300
C -3.34217900,3.95947400,0.40813600
H -4.63141900,2.23997400,-3.69098900
C -4.40509000,4.45955200,-2.14919100
C -3.71051500,5.23753200,0.04083100
H -2.93137600,3.78024200,1.40441600
C -4.24649200,5.49415700,-1.25161600
H -4.82183500,4.64366900,-3.14430500
H -3.59264200,6.06186400,0.75013900
H -4.53692000,6.51209800,-1.52688500
P -2.48730800,-1.16348300,-0.74092000
P 0.10164100,0.87051200,0.08379400
C 1.74092100,-2.41997800,-2.61722100
H 0.71530100,-2.11490100,-2.88204000
H 2.42705100,-1.87349400,-3.28317200
H 1.83705600,-3.49652900,-2.84208200
C 3.43097500,-4.00614500,1.31268400
H 3.98707900,-4.01555900,2.26421400
H 2.91052200,-4.97148300,1.20145900
H 4.16500600,-3.92653600,0.49518700
C -0.55686700,-3.63072300,-0.86761500
C 0.29687200,-3.96409300,1.58969800
N 4.02066100,-1.27660600,0.26424600
C 3.16731100,-1.48336600,1.42997800
C 2.43182100,-2.82494900,1.29734400
C 1.29949500,-3.05312500,2.32953700
H 3.79275300,-1.46418200,2.33172700
H 2.43870500,-0.65725800,1.49610800
H 1.67095600,-3.49237800,3.26854400
H 0.81030800,-2.09121400,2.56664500
H -1.53586800,-4.03385600,-0.58705500
H -0.30256300,-3.73807400,-1.92391700
H 0.61439600,-5.02196200,1.63900500
H -0.73157100,-3.92724600,1.97572100

Ts4
Opt @ BMK/def2SVP in PhCl
SCF Done: E(BMK) = -3790.605248a.u.
Zero-point correction = 1.004082Hartree/Particle
Sum of electronic and thermal Free Energies = -3786.259261a.u.
Imaginary Frequency is -421.60 cm^{-1}

N 5.28583400,0.30115500,0.55479000
C 5.47858800,1.70275900,0.90660200
H 6.40864300,1.77363900,1.49516500
H 5.62227300,2.30415100,-0.01096600

C 4.36750400,2.29769900,1.75715100
C 4.33779700,-0.02805300,-0.50145200
H 4.69974000,-0.92243700,-1.03589900
H 4.31522000,0.81088200,-1.21780600
C 2.92880500,-0.33130500,0.00676600
C 1.88608400,0.43102300,-0.35178300
C 1.56676900,1.52879800,-1.20798400
C 0.82285800,2.69341600,-0.43465000
C 1.99812400,2.21034700,-2.31949000
H 1.47569800,3.12474300,0.34051200
H 0.26056200,3.44205200,-1.00826600
H 1.33950200,2.90423300,-2.85194000
H 3.03541600,2.13691000,-2.67532400
C 3.85328600,3.50010300,1.45471600
S 6.42379200,-0.80515900,1.03287900
O 6.77941400,-0.46278300,2.39432000
O 5.88341300,-2.10279400,0.67808500
C 7.88228300,-0.52680400,0.01357300
C 7.98661300,-1.17340800,-1.22454500
C 8.89143900,0.33060800,0.46905000
C 9.10542400,-0.92803300,-2.02569100
H 7.21396900,-1.88011200,-1.53913800
C 10.00257700,0.56372800,-0.34705100
H 8.81608500,0.78446400,1.46095700
C 10.12744600,-0.05649700,-1.60410300
H 9.19480700,-1.43457500,-2.99209200
H 10.79548100,1.23096700,0.00592500
C 11.35323600,0.17128500,-2.45920400
H 11.75708600,1.18626500,-2.31877900
H 12.15013800,-0.54353000,-2.18656900
H 11.13022100,0.02840100,-3.52802800
H 3.12759700,3.98986700,2.11264100
H 4.17726900,4.05413200,0.56631800
C 3.97773300,1.50311800,2.97815400
H 3.41745000,0.59842000,2.68540900
H 4.87687600,1.15202700,3.51368000
H 3.35189500,2.09655700,3.66201900
C 2.79419800,-1.54382700,0.88979500
H 3.03864100,-2.45655900,0.31544300
H 3.52095700,-1.50705500,1.71814100
H 1.77707500,-1.63794200,1.29573700
Rh -0.08175600,0.86233800,-0.21396300
C 0.13819600,-2.60350700,-0.97566900
C 0.85185900,-2.19708100,-2.12249000
C 0.36606100,-3.89354000,-0.46311800
C 1.74840900,-3.06464800,-2.75320600
H 0.70722800,-1.18745300,-2.51985700
C 1.27544000, -4.75742200,-1.08784700
H -0.14437000,-4.22887900,0.44184000
C 1.96538200,-4.34857800,-2.23392200
H 2.28642700,-2.73537600,-3.64688100
H 1.44681000,-5.75320400,-0.66931000
H 2.67551400,-5.02491700,-2.71840700
C -1.32226600,-2.03186500,1.46688500
C -2.06589900,-3.20709600,1.68915100
C -0.82276000,-1.32052000,2.57492000

C -2.29826200,-3.66212500,2.99046800
H -2.48555800,-3.75761300,0.84105900
C -1.06028500,-1.77358800,3.87794000
H -0.24462700,-0.40207900,2.41507900
C -1.79743400,-2.94568400,4.08584100
H -2.88385000,-4.57179600,3.15062900
H -0.66865000,-1.21093800,4.72990000
H -1.98412100,-3.30176400,5.10305000
C -2.24597900,3.48388300,0.64873700
C -2.97867200,4.59019400,0.18251200
C -1.33131000,3.67993900,1.70757200
C -2.81343300,5.85260300,0.77145600
H -3.67487600,4.48303900,-0.65190600
C -1.17847100,4.93507100,2.30341000
H -0.73502900,2.83527900,2.07243200
C -1.92171900,6.02769500,1.83429700
H -3.38873300,6.70210300,0.39221500
H -0.47477000,5.06291000,3.13097900
H -1.79920100,7.01300900,2.29322700
C -3.27044400,1.97759000,-1.61699500
C -2.61393000,1.71616900,-2.83409000
C -4.62759500,2.35552600,-1.63723600
C -3.29689700,1.84197100,-4.04969800
H -1.56866000,1.38861600,-2.82114700
C -5.30682700,2.48431900,-2.85228900
H -5.16147500,2.53060900,-0.69743300
C -4.64272700,2.22767400,-4.05966400
H -2.77943900,1.62990600,-4.98960800
H -6.36182500,2.77247600,-2.85677400
H -5.17839900,2.32352600,-5.00848100
C -3.42323000,0.85456400,1.07866500
C -3.91557400,-0.40793900,0.72405900
C -3.62632700,1.34168500,2.40831100
C -4.63628100,-1.19411200,1.69510100
C -4.27687900,0.58368000,3.35449500
H -3.26761000,2.33399100,2.68496200
C -4.79210100,-0.70137900,3.03058100
C -5.20243900,-2.46914400,1.38028100
H -4.41746600,0.97425300,4.36719700
C -5.47550200,-1.49173900,4.00212600
C -5.86980500,-3.20668500,2.33664300
H -5.10564500,-2.86512400,0.36745400
C -6.00502700,-2.71917800,3.66535200
H -5.58092200,-1.09840300,5.01804700
H -6.30023400,-4.17637500,2.06994200
H -6.53569500,-3.31547700,4.41308500
C -2.55822500,-1.50727100,-1.14074900
C -3.76259500,-0.96800800,-0.66702400
C -2.50093400,-2.02806200,-2.47275700
C -4.92919900,-0.96144200,-1.51829700
C -3.59660300,-1.99753700,-3.30357200
H -1.57415800,-2.46889300,-2.84234000
C -4.83580800,-1.46409300,-2.85562000
C -6.19433800,-0.45782200,-1.08026600
H -3.52604000,-2.40237300,-4.31802100
C -5.98113100,-1.43809100,-3.70572300

C -7.28964000,-0.45617400,-1.91973300
H -6.29768000, -0.0703960,-0.06515000
C -7.18572700,-0.94679400,-3.24953900
H -5.88676800,-1.82700200,-4.72444000
H -8.24827200,-0.07326700,-1.55777900
H -8.06240000,-0.93928100,-3.90350500
P -0.98530300,-1.39452900,-0.19576400
P -2.34871200,1.80525400,-0.06574200

Int5

Opt @ BMK/def2SVP in PhCl
SCF Done: E(RwB97XD) = -3790.668798a.u.
Zero-point correction = 1.004973Hartree/Particle
Sum of electronic and thermal Free Energies = -3786.308537a.u.

N -5.04259400,-0.21751900,0.01639000
C -5.04644300,-1.35090100,-0.90359000
H -5.86633700,-2.02498800,-0.60169600
H -5.27381300,-0.99541700,-1.92511600
C -3.76395700,-2.16865700,-0.91091700
C -4.30832200,0.98641500,-0.36352600
H -4.83516800,1.86940700,0.03414400
H -4.30867800,1.04903400,-1.46177000
C -2.87511800,0.99189600,0.16851300
C -1.80113100,0.93526300,-0.65670000
C -1.68187900,0.86031400,-2.12602900
C -0.48856500,-0.05846400,-2.24575700
C -2.29523100,1.54548600,-3.10782800
C -3.17974700,-2.50690700,-2.07019000
S -6.12722600,-0.20098300,1.26198000
O -6.10004200,-1.52225600,1.85916700
O -5.82713600,0.99081000,2.03328800
C -7.75866200,0.01488400,0.53040900
C -8.20482800,1.30676200,0.22081800
C -8.55814800,-1.10577800,0.28051700
C -9.45863400,1.46546800,-0.37375500
H -7.58638100,2.17448200,0.46600200
C -9.81116800,-0.92623200,-0.31563600
H -8.20896600,-2.09970700,0.57297900
C -10.27981100,0.35554200,-0.65380600
H -9.81414000,2.47246500,-0.61508100
H -10.44088600,-1.79982900,-0.51182200
C -11.64973700,0.54715900,-1.26394800
H -12.38808400,0.79766500,-0.48143400
H -11.65042300,1.37146900,-1.99481800
H -11.99640400,-0.36751700,-1.76912400
H -2.31008200,-3.17307000,-2.09260800
H -3.56741200,-2.15386500,-3.03197200
C -3.26403400,-2.63244100,0.43324200
H -2.97545700,-1.76760300,1.05606300
H -4.06361200,-3.15114500,0.99092100
H -2.39492100,-3.30232500,0.33136900
C -2.74950400,1.02706300,1.67293100
H -3.19145300,1.95648900,2.07388000
H -3.31559300,0.20551300,2.14317100
H -1.69654700,0.97963100,1.99288400

Rh -0.06656500,0.02365400,-0.26645900
C 0.81682700,3.32496800,-1.04115500
C 0.36472800,3.36694500,-2.37805100
C 0.70025200,4.49598300,-0.26118100
C -0.13361100,4.55036200,-2.93082700
H 0.38800900,2.47155000,-2.99876900
C 0.18983900,5.67502700,-0.81557100
H 0.99905100,4.49883400,0.78765700
C -0.21785900,5.71080900,-2.15321100
H -0.46840900,4.55843400,-3.97190800
H 0.11257000,6.57008100,-0.19195300
H -0.60981900,6.63595700,-2.58542700
C 1.91825300,2.21092900,1.37206300
C 2.97174200,3.12021800,1.59193000
C 1.23736500,1.67458200,2.47836600
C 3.32925100,3.48438600,2.89277000
H 3.51714200,3.54355700,0.74237500
C 1.60030700,2.03235100,3.78192100
H 0.41077300,0.97086800,2.32944500
C 2.64618800,2.93976900,3.98871000
H 4.15012500,4.18920600,3.05162800
H 1.06231500,1.60658800,4.63318800
H 2.92916800,3.22497500,5.00596900
C 0.69415000,-2.91726400,1.12460400
C 0.56336000,-4.27700500,0.78676300
C -0.05411900,-2.41718700,2.21550900
C -0.27197100,-5.11625300,1.53796500
H 1.11000300,-4.68818600,-0.06551100
C -0.87729400,-3.25769200,2.96913800
H 0.02490500,-1.35982400,2.49775200
C -0.98581700,-4.61385900,2.63138900
H -0.36117500,-6.17131500,1.26349400
H -1.44096900,-2.85349300,3.81462400
H -1.63369600,-5.27364900,3.21537000
C 2.53358300,-2.66782300,-1.12012200
C 2.32579100,-2.37689500,-2.47929300
C 3.46644400,-3.66273400,-0.76112000
C 3.03619100,-3.07186100,-3.46671600
H 1.62048000,-1.59303300,-2.76642100
C 4.16370100,-4.36307500,-1.74874600
H 3.65086700,-3.88586200,0.29521500
C 3.95153700,-4.06553700,-3.10289600
H 2.87299200,-2.83463200,-4.52161200
H 4.88156700,-5.13716800,-1.46265500
H 4.50434900,-4.60908200,-3.87455200
C 2.97960800,-1.11749000,1.26237200
C 3.84304300,-0.12338500,0.79093200
C 3.07930600,-1.56679700,2.61465300
C 4.82764900,0.44545700,1.67666900
C 3.99542800,-1.01133000,3.47863600
H 2.42393800,-2.36310800,2.97203100
C 4.87946900,0.01399800,3.04204500
C 5.75497000,1.44767500,1.25230000
H 4.05818300,-1.36320600,4.51315200
C 5.82172500,0.60631700,3.93466100
C 6.66086900,1.99743800,2.13642200

H 5.74539600,1.78508100,0.21368500
C 6.69389900,1.57946600,3.49488800
H 5.84490600,0.26587000,4.97460900
H 7.36331800,2.76033900,1.78831100
H 7.41808300,2.02356800,4.18378900
C 2.94813000,1.25239000,-1.19573200
C 3.85663500,0.32864900,-0.64974500
C 3.13786400,1.70706400,-2.54016700
C 4.92915900,-0.18483000,-1.47165500
C 4.16373900,1.23376200,-3.32422600
H 2.48262500,2.47178100,-2.95287100
C 5.07562500,0.26646800,-2.82335700
C 5.86852900,-1.14827000,-0.98528900
H 4.29126300,1.61187100,-4.34332600
C 6.13457300,-0.23921400,-3.63357900
C 6.88263500,-1.62245000,-1.79162800
H 5.77958200,-1.52097500,0.03657500
C 7.02304500,-1.16444300,-3.12979300
H 6.22943700,0.12549700,-4.66103200
H 7.58710700,-2.35973800,-1.39629900
H 7.83623000,-1.54601000,-3.75372000
P 1.42547800,1.77112200,-0.31294700
P 1.66248300,-1.72120100,0.15257900
H -3.08460600,2.27161500,-2.89214000
H -2.00226300,1.40958600,-4.15491800
H -0.77396900,-1.12218600,-2.37185500
H 0.29320700,0.22325600,-2.97214600

Ts5

Opt @ BMK/def2SVP in PhCl
SCF Done: E(BMK) = -3790.560833a.u.
Zero-point correction = 1.002975Hartree/Particle
Sum of electronic and thermal Free Energies = -3786.214908a.u.
Imaginary Frequency is -843.32cm^{-1}

N -5.13277800,-0.00271400,0.05591300
C -4.51695600,1.25321000,0.35665500
H -5.10858500,1.65667200,1.19543300
H -4.57951000,1.98131100,0.47256600
C -3.07640000,1.13056200,-0.83765600
C -4.84457800,-0.51912100,1.38673200
H -5.66618200,-1.18191100,1.69930200
H -4.78631300,0.32297400,2.09693800
C -3.53825900,-1.31737200,1.40441000
C -2.46145900,-0.79002900,1.94535600
C -1.42044500,-0.20340000,2.46520000
C -0.32455000,0.75135400,2.10335000
C -0.63890400,-0.11956800,3.70397100
C -2.14304000,2.00179200,-0.42493100
S -6.36593500,-0.64442600,-0.84515900
O -6.08077400,-0.29982100,-2.22340900
O -6.50384700,-2.01997200,-0.40708300
C -7.86737100,0.22684100,-0.37143700
C -8.63285800,-0.25506300,0.69794200
C -8.24700300,1.37711100,-1.07385900
C -9.77719300,0.44902100,1.08300300

H -8.34805600,-1.18318600,1.20079000
C -9.39507700,2.06727800,-0.67312000
H -7.66401900,1.70791500,-1.93764800
C -10.17513500,1.61913700,0.40902400
H -10.38148000,0.07502700,1.91570600
H -9.69842900,2.96542500,-1.22056600
C -11.43666100,2.34999900,0.80880100
H -12.30692200,1.93920800,0.26634700
H -11.63756200,2.24388100,1.88640100
H -11.37226200,3.42281900,0.56898200
H -1.12870400,1.97528700,-0.83957800
H -2.37655100,2.78012300,0.31153300
C -2.80058700,0.05073600,-1.85192600
H -2.96830100,-0.94359800,-1.40203100
H -3.50386500,0.12297900,-2.69976200
H -1.76300900,0.10391100,-2.22205800
C -3.56491300,-2.68353000,0.75678100
H -4.17579900,-3.36871200,1.37130800
H -4.06116100,-2.62826400,-0.22592800
H -2.55558200,-3.10554000,0.64673700
Rh 0.96048600,0.48098600,0.50805300
C 1.50087100,-2.47883200,2.36864700
C 2.38743700,-1.94950500,3.33192700
C 0.54719700,-3.42992800,2.78003600
C 2.32526900,-2.36691600,4.66637400
H 3.13719400,-1.21108100,3.02956600
C 0.47767000,-3.83653400,4.11983700
H -0.14852700,-3.85584000,2.05151800
C 1.36453300,-3.30796000,5.06605200
H 3.02941900,-1.95844400,5.39732900
H -0.26980100,-4.57597300,4.42172900
H 1.31436100,-3.63325300,6.10922500
C 0.78326200,-3.12924300,-0.33446900
C 1.40648400,-4.38818900,-0.45119900
C -0.38812600,-2.87240800,-1.06729000
C 0.86234600,-5.36891300,-1.28574000
H 2.32363200,-4.60210500,0.10750600
C -0.93580500,-3.85747400,-1.90028700
H -0.86325100,-1.88746200,-0.99449100
C -0.31039900,-5.10412600,-2.01076300
H 1.35218600,-6.34323500,-1.37231700
H -1.84700700,-3.64660300,-2.46752200
H -0.73422700,-5.87360900,-2.66287200
C 2.11984700,4.04033100,0.48586500
C 3.12683800,5.01711300,0.61523200
C 0.77397000,4.46135100,0.41200600
C 2.79526400,6.37779000,0.63569900
H 4.17439700,4.72437300,0.71300400
C 0.44724000,5.82069700,0.41974800
H -0.02188800,3.71224200,0.34451800
C 1.45960200,6.78312800,0.53083700
H 3.58875600,7.12383300,0.73632600
H -0.59984300,6.12893900,0.35089300
H 1.20616900,7.84710300,0.54696000
C 4.13860000,2.01487100,1.03912300
C 4.40384100,1.35747000,2.25408700

C 5.21879300,2.44900200,0.24533800
C 5.72226000,1.13685200,2.67005800
H 3.56884800,1.01488600,2.87320600
C 6.53571200,2.22661600,0.66016200
H 5.03174300,2.94221400,-0.71328300
C 6.78850000,1.56989600,1.87194400
H 5.91790600,0.62673900,3.61750300
H 7.36620500,2.55641500,0.02958400
H 7.81917600,1.39466200,2.19367600
C 2.13974800,1.54901800,-1.13317000
C 2.37925300,0.15284700,-1.38175100
C 1.37201700,2.32185700,-2.09284800
C 1.84727600,-0.42706400,-2.63348500
C 0.84258200,1.75266400,-3.21177500
H 1.22416500,3.38731500,-1.90467300
C 1.04280800,0.36059500,-3.50901900
C 2.14218200,-1.75935100,-3.02479900
H 0.27286200,2.36465300,-3.91851100
C 0.50673400,-0.21137800,-4.69091400
C 1.61842800,-2.29776200,-4.19208300
H 2.78823900,-2.37723600,-2.39971100
C 0.78121400,-1.52717400,-5.02757400
H -0.11480100,0.41125000,-5.34206300
H 1.86010200,-3.32919700,-4.46336100
H 0.36833500,-1.96029800,-5.94286700
C 3.22498100,-1.64401800,0.19347900
C 3.50091200,-0.65524500,-0.74981400
C 4.27444400,-2.45719400,0.71496800
C 4.83791200,-0.49342300,-1.25023800
C 5.57562600,-2.26138800,0.30682600
H 4.04813000,-3.22488000,1.46024500
C 5.89072700,-1.28429000,-0.68165800
C 5.16375500,0.42046000,-2.29911200
H 6.38307000,-2.87033100,0.72541600
C 7.22934700,-1.09755100,-1.13802300
C 6.46554500, 0.56113500,-2.73551700
H 4.37056500,1.00848800,-2.76775800
C 7.51332200,-0.19466300,-2.14147900
H 8.02628200,-1.69874500,-0.68915200
H 6.69447000,1.25623600,-3.54858800
H 8.54105600,-0.06937400,-2.49432100
P 1.47015700,-1.78801500,0.68323900
P 2.42297600,2.24934700,0.51888300
H 0.45405800,0.48880300,3.02230900
H -0.62417700,1.78845800,2.33268400
H -0.00584100,-0.97248500,3.98841600
H -0.93891500,0.57824900,4.49581100

Int6
Opt @ BMK/def2SVP in PhCl
SCF Done: E(BMK) = -3790.672997a.u.
Zero-point correction = 1.005723Hartree/Particle
Sum of electronic and thermal Free Energies = -3786.315327a.u.

N -5.24851000,0.56970100,-0.32592700
C -5.65101200,1.97019000,-0.25494300

H -6.63262800,2.06030600,-0.75122400
H -5.79802800,2.26030300,0.80254900
C -4.71238400,2.95220100,-0.93998600
C -4.18446600,0.08680200,0.54958900
H -4.41276800,-0.95543900,0.83311000
H -4.19465000,0.69440100,1.46733300
C -2.79799700,0.09063100,-0.09265900
C -1.73211700,0.66242800,0.49706500
C -1.51747200,1.43891000,1.73190300
C -0.20157000,1.19303600,2.03592900
C -2.45256500,2.39618400,2.43131500
C -4.38962200,4.10705400,-0.33788500
S -6.25125100,-0.49260800,-1.10201300
O -6.60563400,0.10934300,-2.37193500
O -5.59992200,-1.78647300,-1.02554300
C -7.76307500,-0.58173800,-0.12464300
C -7.81120300,-1.43682400,0.98472100
C -8.87066400,0.19085400,-0.48923800
C -8.97747700,-1.48821800,1.75105200
H -6.95471600,-2.07353900,1.22222200
C -10.03078700,0.12615700,0.29125300
H -8.82879000,0.81032900,-1.38928900
C -10.10172400,-0.70435400,1.42315900
H -9.02483700,-2.16203400,2.61276700
H -10.90227200,0.72324600,0.00475000
C -11.34875300,-0.75558700,2.27652400
H -11.56371700,-1.78457400,2.60686000
H -11.22368100,-0.13635200,3.18264600
H -12.22554900,-0.37595800,1.72997300
H -3.78930300,4.86857200,-0.84635900
H -4.74495000,4.34385200,0.67135200
C -4.27520900,2.59598400,-2.33924700
H -3.52225300,1.79017100,-2.31314100
H -5.12641000,2.21086200,-2.92652400
H -3.83735000,3.46546800,-2.85295500
C -2.68825600,-0.72326200,-1.36016900
H -2.94712900,-1.77736600,-1.15321000
H -3.41160400,-0.38377300,-2.12074900
H -1.67490000,-0.67866100,-1.78754100
Rh 0.26300600,0.86763000,0.20755000
C -0.45599000,-2.51778700,0.55907500
C -1.21502300,-2.24850400,1.71873000
C -0.78191700,-3.65289100,-0.20836400
C -2.24175400,-3.10910000,2.11553300
H -1.00640900,-1.35995300,2.32062800
C -1.82110900,-4.50620400,0.18544100
H -0.24544300,-3.87517700,-1.13181600
C -2.55004800,-4.24099400,1.34790800
H -2.81012200,-2.88869900,3.02368100
H -2.06315500,-5.37712900,-0.42984500
H -3.36293400,-4.90699200,1.65099900
C 1.35958600,-1.88041000,-1.59902500
C 2.01406700,-3.11037300,-1.80839800
C 1.05594600,-1.07073900,-2.70849900
C 2.35230100,-3.51911700,-3.10146900
H 2.26981300,-3.74733000,-0.95582000

C 1.39820500,-1.47798800,-4.00331200
H 0.54306300,-0.11177800,-2.56797200
C 2.04499600,-2.70396800,-4.19956700
H 2.86452200,-4.47370600,-3.25123800
H 1.15591500,-0.83909100,-4.85682800
H 2.31032400,-3.02615600,-5.21056200
C 2.35739700,3.19445600,-1.13230500
C 3.16545100,4.34515000,-1.06607000
C 1.31735200,3.15201600,-2.08402300
C 2.94220300,5.41832000,-1.93796700
H 3.97127600,4.40893500,-0.32951900
C 1.09754500,4.22132900,-2.95870000
H 0.66549600,2.27127400,-2.15791700
C 1.91242500,5.35857100,-2.88530000
H 3.57669000,6.30700200,-1.87384600
H 0.28715400,4.16937000,-3.69113400
H 1.74040000,6.20004500,-3.56262700
C 3.50204700,2.42892800,1.37396600
C 2.79363500,3.12832900,2.37173000
C 4.90367200,2.35253000,1.45650400
C 3.47494800,3.73142000,3.43381700
H 1.70500000,3.21883300,2.30672900
C 5.58279100,2.95138300,2.52500300
H 5.46870800,1.82968200,0.68105300
C 4.87113400,3.63874400,3.51496400
H 2.91516400,4.27604400,4.19952700
H 6.67286800,2.88220600,2.58009800
H 5.40366300,4.10642500,4.34813000
C 3.70330300,0.63931200,-0.94213000
C 3.99537900,-0.64398300,-0.46208000
C 4.18962500,1.04246000,-2.22595800
C 4.78381000,-1.54447100,-1.26883200
C 4.92292200,0.18522900,-3.01263400
H 3.97469900,2.04599700,-2.59622100
C 5.22886000,-1.12906600,-2.56556900
C 5.12403000,-2.86494700,-0.83595900
H 5.28080300,0.51146100,-3.99406900
C 5.97072200,-2.03212600,-3.38401600
C 5.84621900,-3.71620700,-1.64715200
H 4.80606000,-3.20875600,0.15008200
C 6.27349800,-3.30073300,-2.93812800
H 6.29983900,-1.69452800,-4.37170100
H 6.09590600,-4.72026300,-1.29196100
H 6.84718200,-3.98583600,-3.56884400
C 2.27406300,-1.58910700,1.19272700
C 3.57074500,-1.13443000,0.90123200
C 1.99217300,-2.13351900,2.48615300
C 4.59630400,-1.21842600,1.91673700
C 2.95788500,-2.20342300,3.46202400
H 0.99674700,-2.51930800,2.70570500
C 4.27881900,-1.74351200,3.21157000
C 5.94770600,-0.81263500,1.67678400
H 2.71728700,-2.62980700,4.44078200
C 5.28967600,-1.82425900,4.21524400
C 6.90721100,-0.90834000,2.66316900
H 6.22826800,-0.43206800,0.69337900

C 6.57816600,-1.41468400,3.95040700
H 5.02203700,-2.22618600,5.19731500
H 7.93410100,-0.59692300,2.45129100
H 7.34990900,-1.48451200,4.72213400
P 0.85355700,-1.35441000,0.05629900
P 2.57529400,1.75560100,-0.03229200
H -3.17629400,1.83563000,3.04871200
H -1.90337600,3.08573600,3.09027700
H -3.02092800,2.97675100,1.68608000
H 0.40957500,1.38856900,2.92438800

Ts6

Opt @ BMK/def2SVP in PhCl
SCF Done: E(BMK) = -3790.641215a.u.
Zero-point correction = 1.009726Hartree/Particle
Sum of electronic and thermal Free Energies = -3786.293402a.u.
Imaginary Frequency is -258.82cm^{-1}

N -4.63667400,0.69335700,0.10785700
C -3.79112000,1.78965900,0.54868300
H -4.17602500,2.71093800,0.07890200
H -3.83685800,1.92235500,1.64049700
C -2.33926200,1.65045200,0.09828100
C -4.10530700,-0.67512200,0.20879700
H -3.59742700,-0.96129000,-0.73066500
H -4.95307100,-1.36868100,0.33564700
C -3.16307300,-0.78638100,1.38738800
C -2.03268000,-0.04642800,1.45151900
C -1.18769200,0.18809300,2.65945700
C 0.02392300,0.57709200,2.22644000
C -1.64552800,0.11955800,4.10470500
C -1.27993400,2.50931300,0.57583500
S -5.70296500,0.94076700,-1.15533900
O -5.82311100,2.37595100,-1.31413000
O -5.28617900,0.10351900,-2.27010700
C -7.25550000,0.28198100,-0.54433400
C -7.72346800,-0.94073600,-1.03197600
C -7.98851100,1.02766000,0.38935300
C -8.94428500,-1.43451200,-0.55469900
H -7.14482400,-1.48285100,-1.78448300
C -9.20186500,0.51857200,0.85142800
H -7.61718100,1.99672600,0.73403200
C -9.69895500,-0.71830600,0.38930400
H -9.31957800,-2.39068600,-0.93243100
H -9.78358300,1.09433600,1.57860500
C -11.03011700,-1.23661500,0.88321500
H -11.17943700,-2.29165700,0.60815500
H -11.10922700,-1.14736900,1.97907000
H -11.85798600,-0.65212300,0.44467200
C -2.17904100,1.31977700,-1.39295800
H -2.14258900,2.26816500,-1.95819000
H -1.21453000,0.80316300,-1.64451700
H -2.97962400,0.69314700,-1.80790600
C -3.64345500,-1.71089800,2.48445800
H -3.91939900,-2.68295400,2.03807800
H -2.88675600,-1.90341600,3.25222000

H -4.55567000,-1.30990900,2.96527500
Rh -0.27592000,0.66382400,0.28480000
C -0.16011100,-3.04778000,0.53108200
C -1.45869300,-3.16978700,-0.00020900
C 0.38799900,-4.14232800,1.22840200
C -2.19741800,-4.34503100,0.17171900
H -1.88577800,-2.34400800,-0.57545800
C -0.35777800,-5.31369500,1.41241300
H 1.40435800,-4.09391400,1.62729000
C -1.65154400,-5.41773900,0.88834000
H -3.19822000,-4.42645700,-0.26266300
H 0.08185300,-6.15210900,1.96038700
H -2.22836100,-6.33657300,1.02790300
C 1.34841700,-2.08566700,-1.59061400
C 2.03907100,-3.30378100,-1.74631800
C 1.02929900,-1.33937800,-2.73737300
C 2.41719800,-3.74894400,-3.01534500
H 2.28334400,-3.91191800,-0.87024600
C 1.40088200,-1.78714500,-4.01138100
H 0.48736400,-0.39427100,-2.63922000
C 2.09838300,-2.99174100,-4.15129600
H 2.95849600,-4.69348600,-3.11896300
H 1.14129600,-1.19482300,-4.89323200
H 2.38760300,-3.34583900,-5.14491400
C 1.42889100,3.13002600,-1.44322000
C 1.77790300,4.46618700,-1.17488300
C 0.70561800,2.84912500,-2.62274600
C 1.41019300,5.49036200,-2.06033700
H 2.33998900,4.71832400,-0.27279800
C 0.34753000,3.86850500,-3.50886000
H 0.44060300,1.81671400,-2.86948000
C 0.69550300,5.19737700,-3.22542200
H 1.69000000,6.52294000,-1.83275100
H -0.20374000,3.62639400,-4.42211900
H 0.41080700,5.99828400,-3.91371700
C 2.65845300,2.61833200,1.08091900
C 1.89495800,3.23499000,2.09287300
C 4.05553500,2.78573500,1.08618400
C 2.51779500,3.99204000,3.09073500
H 0.80770600,3.13160900,2.09406600
C 4.67696800,3.53703100,2.09090300
H 4.66509700,2.33492900,0.29996300
C 3.91106800,4.14014400,3.09507700
H 1.91181600,4.46706400,3.86751300
H 5.76445600,3.65214500,2.08426400
H 4.39794900,4.72803300,3.87858300
C 3.11688400,0.79625400,-1.15866200
C 3.67054900,-0.37385300,-0.61914400
C 3.51135000,1.21035900,-2.47129600
C 4.57275000,-1.17268200,-1.41048500
C 4.37141300,0.45648200,-3.23579300
H 3.12928700,2.14543100,-2.88042200
C 4.90460100,-0.76428600,-2.74158300
C 5.13991900,-2.39029100,-0.91954900
H 4.65400700,0.79647600,-4.23697300
C 5.76731700,-1.57534400,-3.53740500

C 5.97260300,-3.15488900,-1.71028400
H 4.90610600,-2.72136300,0.09435100
C 6.28996200,-2.74788800,-3.03534300
H 6.00891400,-1.24581000,-4.55267900
H 6.39500100,-4.08291400,-1.31427600
H 6.95329200,-3.36366900,-3.64942700
C 2.20665500,-1.43249300,1.17474500
C 3.40408300,-0.82149800,0.79374600
C 2.00298900,-1.78365400,2.55057700
C 4.45559000,-0.62936100,1.76880100
C 2.98519300,-1.59065300,3.49109200
H 1.04262700,-2.19486500,2.86386200
C 4.23940100,-1.02309200,3.12859700
C 5.72738700,-0.07005800,1.43037700
H 2.80950900,-1.87086500,4.53438100
C 5.27326100,-0.84167300,4.09389700
C 6.71376300,0.08689100,2.38334500
H 5.92955700,0.22611000,0.39955600
C 6.48773700,-0.29882900,3.73206800
H 5.08500700,-1.14783400,5.12766000
H 7.68159500,0.50794100,2.09609500
H 7.27836600,-0.16844800,4.47635100
P 0.76601500,-1.54935300,0.05474600
P 1.81796300,1.76531000,-0.28654800
H -1.58756700,-0.90139000,4.51754200
H -1.00857500,0.76564300,4.72920300
H -2.68915000,0.45944300,4.20728900
H 0.90930900,0.83827500,2.81476700
H -1.37998800,2.87434500,1.60594700
H -0.88692300,3.25300900,-0.12870800

Int7
Opt @ BMK/def2SVP in PhCl
SCF Done: E(BMK) = -3790.706982a.u.
Zero-point correction = 1.011571Hartree/Particle
Sum of electronic and thermal Free Energies = -3786.356477a.u.

N 5.17539900,-0.56549800,0.20866700
C 4.05143300,-1.24464900,-0.40405500
H 4.09701100,-1.10791900,-1.49301000
H 4.13451000,-2.32506600,-0.18587700
C 2.70935900,-0.72650500,0.14563500
C 5.23830300,-0.70524600,1.64771500
H 5.79698700,0.14822000,2.06786100
H 5.81208800,-1.61716900,1.91615700
C 3.89144300,-0.77140600,2.36059100
C 2.70325700,-0.78417200,1.69279200
C 1.34798500,-0.80265100,2.32956400
C 0.21282300,-0.57437200,1.62374500
C 1.16152000,-1.11682400,3.80986500
C 1.55426000,-1.52737000,-0.47657200
S 5.84604600,0.77123400,-0.50104500
O 5.34796300,0.78499400,-1.86412900
O 5.65294800,1.92523900,0.36354600
C 7.60850700,0.42283200,-0.52567900
C 8.46498400,1.15217100,0.30157300

C 8.09846200,-0.55583100,-1.40221300
C 9.83863700,0.87879400,0.26246100
H 8.05920800,1.92866300,0.95489300
C 9.46863900,-0.81262000,-1.42699800
H 7.41426400,-1.09875300,-2.06017200
C 10.36050400,-0.10228900,-0.59573700
H 10.51572100,1.44650900,0.90823300
H 9.86021500,-1.57438400,-2.10909300
C 11.84393800,-0.38751700,-0.65303800
H 12.38887600,0.15097600,0.13687800
H 12.04361600,-1.46599700,-0.53833100
H 12.26148100,-0.07750000,-1.62679600
C 2.48758000,0.74741300,-0.31029100
H 3.23036400,1.43545700,0.12911400
H 2.53202400,0.85561100,-1.40732200
H 1.53623300,1.21842300,0.07922800
C 4.11896500,-0.80904300,3.86561500
H 5.19941200,-0.80107800,4.07963600
H 3.68735700,0.07018900,4.36850300
H 3.70645600,-1.70948400,4.34180300
Rh 0.10092400,-0.11029800,-0.29208200
C -0.57167600,3.27572400,0.85269300
C 0.44275700,3.02439200,1.80485900
C -0.80198500,4.60724300,0.45425200
C 1.18431300,4.07450700,2.35467400
H 0.65150100,1.99927700,2.12803900
C -0.05113400,5.65545800,1.00292400
H -1.56177000,4.83869700,-0.29477500
C 0.94015800,5.39474500,1.95412100
H 1.96326300,3.85827300,3.09115200
H -0.24402100,6.68163000,0.67722800
H 1.52677400,6.21516500,2.37727200
C -2.48059000,2.60231100,-1.18743600
C -3.62441000,3.36206100,-0.87318000
C -2.11141600,2.45252800,-2.53599600
C -4.38532700,3.94895600,-1.88838800
H -3.92625600,3.48659800,0.17162800
C -2.87290100,3.04029300,-3.55388000
H -1.21340500,1.88117200,-2.79506600
C -4.01237000,3.78614000,-3.22996900
H -5.27465000,4.53171600,-1.63207100
H -2.57386900,2.91736000,-4.59856200
H -4.60943000,4.24535200,-4.02313600
C -0.94499200,-2.26370600,-2.38922800
C -0.70081900,-3.62967000,-2.60163900
C -0.54004800,-1.33349100,-3.37250200
C -0.06988300,-4.05712500,-3.77991600
H -0.99604700,-4.36682400,-1.85033500
C 0.08755200,-1.76166600,-4.54431500
H -0.73923300,-0.26394800,-3.23093300
C 0.32520000,-3.12954100,-4.74887300
H 0.11295100,-5.12415900,-3.93586400
H 0.39132200,-1.03084100,-5.29914000
H 0.81777200,-3.46824000,-5.66479900
C -2.02487700,-3.05025800,0.17777200
C -1.15576300,-3.54052500,1.16998300

C -3.21564800,-3.75302700,-0.10391700
C -1.47667900,-4.71065000,1.87007600
H -0.23163400,-3.00731300,1.40133600
C -3.52695000,-4.92314300,0.59440900
H -3.90037800,-3.38912000,-0.87594400
C -2.65892900,-5.40255700,1.58477000
H -0.79648300,-5.08096800,2.64210200
H -4.45045600,-5.46205700,0.36433500
H -2.90556100,-6.31633300,2.13327700
C -3.19956100,-0.84048700,-1.27681300
C -3.92751400,-0.07232200,-0.36228700
C -3.70288500,-1.03019400,-2.60427600
C -5.17975700,0.52170600,-0.76957500
C -4.87703800,-0.44282600,-3.01201100
H -3.14928400,-1.65157600,-3.30912600
C -5.64057800,0.35376000,-2.11540900
C -5.98377000,1.30211500,0.11995900
H -5.23972700,-0.59341100,-4.03349600
C -6.85547700,0.97334500,-2.53296000
C -7.15899500,1.88450900,-0.30915400
H -5.66579800,1.44375800,1.15437300
C -7.60129300,1.72525300,-1.65054300
H -7.18721900,0.83355000,-3.56644700
H -7.75820100,2.47312600,0.39154700
H -8.53516900,2.19275200,-1.97553600
C -2.58434700,1.19883000,1.38321600
C -3.49740300,0.18463300,1.06026300
C -2.37799000,1.54792000,2.75320500
C -4.17245000,-0.53391400,2.11100700
C -3.03062100,0.88136100,3.76646900
H -1.69625200,2.36206000,3.00505000
C -3.92268500,-0.18844300,3.47997400
C -5.10752000,-1.58182700,1.84093700
H -2.86722300,1.17405800,4.80841800
C -4.59109700,-0.89928200,4.52145500
C -5.73765100,-2.25226500,2.86856700
H -5.32396800,-1.85248800,0.80562200
C -5.47853500,-1.91108700,4.22497500
H -4.38906800,-0.62070000,5.56040200
H -6.44789200,-3.05171500,2.63940100
H -5.98973800,-2.44924200,5.02816100
P -1.45032700,1.86039900,0.10603500
P -1.60257600,-1.60481200,-0.82707000
H 1.59286600,-0.34581800,4.46504000
H 0.08870400,-1.18704400,4.04415200
H 1.62745800,-2.07937900,4.07474800
H -0.75508600,-0.64471000,2.13796000
H 1.40804700,-2.51677400,-0.01556700
H 1.72941300,-1.66508000,-1.56178800

Ts7

Opt @ BMK/def2SVP in PhCl
SCF Done: E(BMK) = -3790.671739a.u.
Zero-point correction = 1.011044Hartree/Particle
Sum of electronic and thermal Free Energies = -3786.325157a.u.
Imaginary Frequency is -413.22cm^{-1}

N -5.27641300,-0.57330900,-0.29385400
C -4.17737100,-1.28847900,0.33094400
H -4.17094500,-1.06861200,1.40718200
H -4.33772200,-2.37346600,0.19566800
C -2.84796600,-0.88548100,-0.32919700
C -5.42609400,-0.76912700,-1.72599500
H -5.91800400,0.11976600,-2.15557500
H -6.09924900,-1.62931800,-1.92721700
C -4.12748700,-1.00494800,-2.48710000
C -2.93348200,-1.08923600,-1.84688600
C -1.60374700,-1.44362700,-2.38237300
C -0.58056600,-1.48712500,-1.48419000
C -1.35550000,-1.89671200,-3.80659000
C -1.66207300,-1.70720700,0.18924600
S -5.86401100,0.81960000,0.38801900
O -5.27177600,0.89103900,1.71112900
O -5.69525200,1.91627000,-0.55214900
C -7.62918800,0.53351600,0.55451500
C -8.51728800,1.20050500,-0.29234400
C -8.08709300,-0.33853000,1.55245000
C -9.89162300,0.97033400,-0.14752000
H -8.13499600,1.89812400,-1.04188900
C -9.45886600,-0.55349900,1.68108500
H -7.37711600,-0.82956800,2.22368100
C -10.38280500,0.09437300,0.83416100
H -10.59344300,1.48954400,-0.80754500
H -9.82565100,-1.23156900,2.45859700
C -11.86628100,-0.13993800,1.00570300
H -12.44282800,0.30575600,0.18116100
H -12.09448100,-1.21794700,1.04586500
H -12.22248900,0.30729000,1.95015900
C -2.51591400,0.60050900,-0.04810300
H -3.25853700,1.28166000,-0.49700500
H -2.45139900,0.83247000,1.02760800
H -1.58699200,0.92700800,-0.60540600
C -4.37887600,-1.18816400,-3.97061500
H -4.54237100,-2.25206200,-4.22180300
H -5.29219400,-0.64687800,-4.26821800
H -3.55764300,-0.81358800,-4.59286400
Rh -0.09733400,-0.22960200,0.05625700
C 0.53000300,3.10430100,-1.08075100
C -0.70434600,3.56265600,-0.57860800
C 1.15207600,3.83583200,-2.11331500
C -1.31417100,4.70598100,-1.10846700
H -1.19166100,3.03821300,0.24913900
C 0.53995900,4.97689400,-2.64345100
H 2.11781400,3.51567900,-2.51401100
C -0.69557300,5.41181100,-2.14669900
H -2.27400200,5.04233100,-0.70648000
H 1.03355600,5.52993400,-3.44784900
H -1.17253300,6.30278000,-2.56505500
C 2.26715100,2.56394400,1.05029200
C 3.18017500,3.58225500,0.71198400
C 2.01530800,2.29550200,2.40616500
C 3.83745700,4.30297400,1.71185100

H 3.37997700,3.81637700,-0.33792100
C 2.66773300,3.02458800,3.40856800
H 1.31080900,1.50460600,2.68190800
C 3.58063500,4.02662400,3.06255500
H 4.54769600,5.08775400,1.43696500
H 2.45966700,2.80762600,4.46005500
H 4.08890000,4.59877100,3.84405800
C 1.11328900,-2.14860700,2.53878800
C 1.48958200,-3.41844500,3.01824900
C 0.29163700,-1.33687700,3.34833400
C 1.05404400,-3.86159900,4.27364800
H 2.12795100,-4.06759300,2.41355300
C -0.13832600,-1.77891100,4.60428900
H -0.02208900,-0.34948800,2.99392700
C 0.24138800,-3.04497200,5.06894400
H 1.35315500,-4.85172400,4.62949600
H -0.77525100,-1.13494900,5.21744500
H -0.09805400,-3.39467800,6.04823400
C 2.19356300,-3.03607500,0.05683100
C 1.20409200,-3.97056400,-0.31742900
C 3.54200400,-3.34968500,-0.18381600
C 1.55720000,-5.17505600,-0.93148300
H 0.15111600,-3.76845800,-0.10947000
C 3.89414200,-4.55482000,-0.80642300
H 4.32730700,-2.66000400,0.12636500
C 2.90496700,-5.46734300,-1.18503000
H 0.77739000,-5.88953400,-1.21001600
H 4.94911300,-4.77779700,-0.98877900
H 3.18079500,-6.40879500,-1.66879400
C 3.26159500,-0.63855800,1.32841900
C 3.97872700,0.12894700,0.39728900
C 3.72232500,-0.69905900,2.68238100
C 5.13414200,0.87785500,0.82218300
C 4.82879800,0.00498400,3.09712200
H 3.18553700,-1.30888800,3.40956000
C 5.55264200,0.82492400,2.19035900
C 5.87471300,1.70857200,-0.07625400
H 5.15641800,-0.05627100,4.13953500
C 6.67709500,1.59179200,2.61747700
C 6.95831600,2.44013400,0.36420200
H 5.57714100,1.76707700,-1.12510100
C 7.36674400,2.38443200,1.72523500
H 6.98173500,1.53710700,3.66717200
H 7.51044000,3.06878800,-0.34035900
H 8.22936000,2.96847900,2.05851500
C 2.45608400,0.93374200,-1.44866700
C 3.57845200,0.20521000,-1.05113200
C 2.03019700,0.89299100,-2.81666000
C 4.37189300,-0.48679600,-2.04142200
C 2.75242600,0.20769500,-3.76299600
H 1.11230900,1.40789200,-3.10902700
C 3.94909400,-0.48029500,-3.41044300
C 5.58626100,-1.17023800,-1.72050700
H 2.41684400,0.19036500,-4.80453300
C 4.72634500,-1.15750900,-4.39566200
C 6.32430600,-1.80932200,-2.69673800

H 5.94920300,-1.17225200,-0.69085600
C 5.89081200,-1.80982300,-4.04996600
H 4.38351800,-1.14278700,-5.43495600
H 7.25642800,-2.31416300,-2.42697600
H 6.48513000,-2.32076100,-4.81267400
P 1.31871600,1.69273400,-0.23644200
P 1.69232300,-1.51371300,0.92415800
H -1.43896000,-1.06072500,-4.52088600
H -0.34075200,-2.31197500,-3.90325000
H -2.07665600,-2.67100200,-4.11519900
H 0.34413900,-2.01187700,-1.75426000
H -1.74595100,-2.78570700,0.00118100
H -1.53814400,-1.57862900,1.28787000

Pro2

Opt @ BMK/def2SVP in PhCl
SCF Done: E(BMK) = -3790.745303a.u.
Zero-point correction = 1.013822Hartree/Particle
Sum of electronic and thermal Free Energies = -3786.405101a.u.

N -5.04567500,0.32132400,0.14416600
C -4.59787100,0.93316500,-1.09642100
H -4.98057500,0.35091400,-1.94665100
H -4.99060200,1.96446900,-1.17011800
C -3.06003800,0.95509600,-1.08690600
C -4.68708700,0.98921300,1.37844600
H -4.69858300,0.24520100,2.19183200
H -5.43178700,1.77121500,1.64861300
C -3.32210000,1.65612800,1.33605000
C -2.58718300,1.65962000,0.20031900
C -1.33222200,2.40408200,-0.12456700
C -1.15620200,2.35677000,-1.52027100
C -0.74228100,3.49610400,0.73903600
C -2.39467800,1.79041200,-2.20573300
S -5.86224400,-1.11010700,0.15758900
O -5.50809300,-1.79971200,-1.06788300
O -5.62611200,-1.70410900,1.46185200
C -7.61204300,-0.70543100,0.06321300
C -8.32097300,-0.44627300,1.24227200
C -8.23964700,-0.65458800,-1.18726100
C -9.67381400,-0.10424700,1.15720800
H -7.82444400,-0.53947100,2.21187400
C -9.59331600,-0.31174900,-1.25090900
H -7.67906300,-0.90778900,-2.09098500
C -10.32931500,-0.02469800,-0.08581000
H -10.23607100,0.08982800,2.07614600
H -10.09249800,-0.28080900,-2.22474100
C -11.78639700,0.36908400,-0.17140200
H -12.32020400,0.13910100,0.76362800
H -11.88304100,1.45439700,-0.35350100
H -12.29316700,-0.15177300,-0.99911800
C -2.53560100,-0.48907100,-1.16770100
H-2.82805200,-1.09572200,-0.30013300
H -2.89940100,-0.98380500,-2.08393500
H -1.41907900,-0.55507700,-1.26675700
C -2.96550000,2.35355000,2.63340300

H -3.19441700,3.43328800,2.59073000
H -3.54991300,1.93334900,3.46821200
H -1.90161000,2.24691100,2.88556800
Rh 0.00331600,0.69771900,-0.61345900
C -0.48348600,-2.24293300,0.89444400
C -1.21238400,-1.53013100,1.87388400
C -0.99632700,-3.46650300,0.42923500
C -2.41720000,-2.03018000,2.37062600
H -0.85029100,-0.55661700,2.22248300
C -2.21286800,-3.96006200,0.92436200
H -0.46523400,-4.02918100,-0.34223700
C -2.92936200,-3.24364000,1.88687700
H -2.97681400,-1.46512300,3.12139200
H -2.60762900,-4.90360000,0.53719300
H -3.89860400,-3.60245000,2.24100300
C 1.53362200,-2.53522300,-1.17420500
C 2.12874900,-3.76970300,-0.84915700
C 1.41861700,-2.15971200,-2.52523300
C 2.59487000,-4.61381000,-1.86127900
H 2.23647200,-4.06710200,0.19942200
C 1.88971100,-3.00510300,-3.53689400
H 0.97589500,-1.19057300,-2.78155200
C 2.47868500,-4.23115400,-3.20520000
H 3.05775100,-5.57001800,-1.60149700
H 1.80070000,-2.70329300,-4.58420800
H 2.84946700,-4.89136000,-3.99454900
C 2.20675400,3.18887600,-1.54540000
C 2.62889200,4.43984100,-1.05708000
C 1.75603200,3.11323000,-2.88292100
C 2.63170100,5.56905400,-1.88945900
H 2.94787900,4.55140300,-0.01915100
C 1.77380600,4.23480700,-3.71574100
H 1.37085700,2.16504300,-3.27224400
C 2.21658200,5.46963100,-3.22026800
H 2.96231500,6.53095200,-1.48725000
H 1.43029000,4.14780700,-4.75059100
H 2.22582200,6.35088100,-3.86806600
C 2.73725100,2.16547800,1.12028900
C 1.95868100,1.95981900,2.27311500
C 4.03416700,2.69938500,1.25968400
C 2.45644800,2.29453100,3.53738900
H 0.97000100,1.50239100,2.17489800
C 4.52615300,3.04557500,2.52174400
H 4.66916800,2.83401500,0.37784800
C 3.73813200,2.84432300,3.66262800
H 1.84483900,2.11903500,4.42684900
H 5.53387500,3.45967300,2.61649800
H 4.12745900,3.10863700,4.64996300
C 3.39835400,0.54086200,-1.17396300
C 3.87184200,-0.55380200,-0.43487400
C 3.84996600,0.71094700,-2.52163000
C 4.76658400,-1.50732100,-1.05100600
C 4.70346800,-0.18762200,-3.11818100
H 3.53888600,1.58259600,-3.09475400
C 5.17483100,-1.32578500,-2.41080800
C 5.26283800,-2.65354900,-0.35325300

H 5.03641700,-0.02303700,-4.14775600
C 6.05128600,-2.26937800,-3.02396300
C 6.11373700,-3.54857300,-0.96865000
H 4.96112200,-2.82659600,0.68104700
C 6.51570800,-3.35863100,-2.31855700
H 6.35215700,-2.10716100,-4.06367500
H 6.48184700,-4.41503000,-0.41170600
H 7.19283600,-4.07542600,-2.79175500
C 2.25629500,-1.31615000,1.36427800
C 3.50810400,-0.80768800,1.00521000
C 1.98278000,-1.63934800,2.72874200
C 4.51024300,-0.59291900,2.01788600
C 2.92843900,-1.43206900,3.70749100
H 1.01709100,-2.07011200,2.99928200
C 4.20575200,-0.89457800,3.38530700
C 5.81208300,-0.08624000,1.71385800
H 2.70758900,-1.69227600,4.74748800
C 5.19029800,-0.67152600,4.39343300
C 6.74777600,0.11037800,2.70861600
H 6.06957900,0.14873400,0.67909500
C 6.43621200,-0.18076000,4.06486000
H 4.94030200,-0.90768800,5.43251800
H 7.73949500,0.49402600,2.45205800
H 7.18860800,-0.02104600,4.84251500
P 0.93266900,-1.41172400,0.11131500
P 2.10326500,1.68139400,-0.51258400
H -0.40373400,3.15027700,1.72322500
H 0.10750100,3.97439000,0.22769800
H -1.51152400,4.27167200,0.90360400
H -0.52122300,3.07833900,-2.03885300
H -3.03756200,2.63943700,-2.50913900
H -2.18536300,1.19588100,-3.11062300

Ts8

Opt @ BMK/def2SVP in PhCl
SCF Done: E(BMK) = -3790.598327a.u.
Zero-point correction = 1.006677Hartree/Particle
Sum of electronic and thermal Free Energies = -3786.263108a.u.
Imaginary Frequency is -1790.74cm^{-1}

N -4.90904500,0.08011900,0.27518200
C -4.66135700,0.82962400,-0.94699400
H -5.09412200,0.28483300,-1.79908900
H -5.13931700,1.82670500,-0.88584600
C -3.13790100,0.98185600,-1.11477500
C -4.53523100,0.72666900,1.51527700
H -4.52115200,-0.02931600,2.31637000
H -5.27858100,1.49989100,1.81690000
C -3.18464100,1.40656500,1.40924700
C -2.55887700,1.51746800,0.21531300
C -1.42014600,2.38697600,-0.15521600
C -1.36442700,2.52536500,-1.59344400
C -0.79270100,3.58216800,0.36327400
C -2.69671000,2.04775700,-2.17192800
S -5.83553700,-1.28322400,0.26321100
O -5.51654600,-1.98271800,-0.96550700

O -5.65493600,-1.90002900,1.56516300
C -7.54792000,-0.74470500,0.15239400
C -8.23162200,-0.39348300,1.32428600
C -8.16880600,-0.67129900,-1.09941300
C -9.54870300,0.06146700,1.22783900
H -7.74312600,-0.49934200,2.29687400
C -9.48899100,-0.21419700,-1.17444200
H -7.63109600,-0.99076200,-1.99605000
C -10.19759700,0.16036700,-0.01875300
H -10.08979200,0.33436400,2.13962400
H -9.98150200,-0.15820300,-2.15038600
C -11.63562400,0.61892800,-0.10189000
H -12.31985100,-0.21947100,0.11958500
H -11.84376500,1.41662800,0.62889500
H -11.88111300,0.99365500,-1.10743300
C -2.54383900,-0.38846100,-1.49220300
H -2.80132900,-1.15990000,-0.75440800
H -2.91741100,-0.71069900,-2.47901100
H -1.42782800,-0.39083900,-1.63067000
C -2.64815600,1.99236000,2.68640100
H -3.46823100,2.34587900,3.33475800
H -2.08678900,1.23447400,3.26415800
H -1.96511900,2.83478900,2.48610200
Rh 0.03511500,0.64546000,-0.72360200
C -0.50020900,-2.35321700,0.50078400
C -1.34743400,-1.77439100,1.47321100
C -0.90617600,-3.54730000,-0.12281500
C -2.55885100,-2.37853500,1.81338300
H -1.07065100,-0.82357500,1.94099400
C -2.13141600,-4.14230800,0.21196300
H -0.28115100,-4.00756800,-0.89140100
C -2.96264700,-3.55879200,1.17217800
H -3.21760500,-1.92333500,2.55683700
H -2.44019300,-5.06014200,-0.29623300
H -3.93634500,-3.99403800,1.41134200
C 1.73090800,-2.42981700,-1.33561900
C 2.34186800,-3.65180800,-0.99281000
C 1.73386100,-2.01062300,-2.67779600
C 2.94215700,-4.43967400,-1.97924200
H 2.35470000,-3.98512600,0.05041200
C 2.34014400,-2.79902100,-3.66345100
H 1.27163500,-1.05352800,-2.94219600
C 2.94507200,-4.01249500,-3.31475700
H 3.41622800,-5.38623100,-1.70477300
H 2.34220500,-2.46316500,-4.70422900
H 3.41990000,-4.62855700,-4.08381400
C 2.18113900,3.14735800,-1.55411700
C 2.49585800,4.42541800,-1.05665700
C 1.82423600,3.02675700,-2.91622500
C 2.47495200,5.54398100,-1.90086400
H 2.74363700,4.55993400,-0.00184800
C 1.80794300,4.14257400,-3.75827100
H 1.55566400,2.04620300,-3.32437500
C 2.13605400,5.40740800,-3.25093600
H 2.72146700,6.52886400,-1.49395800
H 1.53576900,4.02472100,-4.81113800

H 2.12156300,6.28282000,-3.90654800
C 2.57241400,2.19121000,1.16825800
C 1.68993000,1.99378300,2.24611300
C 3.83896300,2.75617500,1.41471900
C 2.06431800,2.35744500,3.54449700
H 0.71569700,1.52855000,2.06258100
C 4.20661700,3.13072800,2.71085500
H 4.54856300,2.89106100,0.59150300
C 3.32163700,2.92847500,3.77803400
H 1.37555800,2.18873900,4.37697300
H 5.19287900,3.56763100,2.89076100
H 3.61463400,3.21268400,4.79275000
C 3.50646100,0.59553200,-1.04723200
C 3.93029700,-0.49826700,-0.28111200
C 4.09491700,0.81506500,-2.33271700
C 4.91953100,-1.40776800,-0.80922100
C 5.03563200,-0.04571100,-2.84902200
H 3.81377900,1.69063700,-2.91680300
C 5.46305900,-1.18501400,-2.11474700
C 5.37819900,-2.54883800,-0.07910000
H 5.47320900,0.14923200,-3.83315100
C 6.42760500,-2.09109700,-2.64684000
C 6.31666100,-3.40646400,-0.61558100
H 4.97644500,-2.74677300,0.91623300
C 6.84924400,-3.17977500,-1.91382600
H 6.83108100,-1.90060100,-3.64621400
H 6.65372700,-4.27078000,-0.03607800
H 7.59374400,-3.86750400,-2.32494300
C 2.12996000,-1.30924600,1.31478400
C 3.40755500,-0.78042500,1.10270700
C 1.71659400,-1.64922500,2.64014000
C 4.28337700,-0.54792300,2.22405900
C 2.54096600,-1.42838700,3.72008700
H 0.74173300,-2.11031700,2.80367500
C 3.83347200,-0.86009300,3.54772000
C 5.59857400,-0.00853200,2.07050500
H 2.21028100,-1.70315200,4.72670000
C 4.69149600,-0.61841700,4.66148600
C 6.41052900,0.20635100,3.16520000
H 5.96501300,0.23870900,1.07213600
C 5.95476500,-0.09764200,4.47687700
H 4.33057900,-0.86401800,5.66517700
H 7.41493300,0.61557100,3.02308200
H 6.61017100,0.07669300,5.33499700
P 0.93914800,-1.39916400,-0.07205600
P 2.10254200,1.65449500,-0.50156300
H -0.23314100,3.62392600,1.30758800
H -0.72037200,3.67413500,-1.17603000
H -1.29845400,4.52248400,0.11043900
H -3.40977800,2.89172800,-2.18521800
H -2.63530400,1.63320700,-3.19021800
H -0.41533500,2.38799200,-2.15138500

Int8

Opt @ BMK/def2SVP in PhCl

SCF Done: E(BMK) = -3867.148006a.u.

Zero-point correction = 1.040670Hartree/Particle
Sum of electronic and thermal Free Energies = -3862.686511a.u.

C 4.51271800,-2.51357200,-0.32837200
H 4.93556000,-3.45949800,0.07282400
H 5.35062300,-1.95252100,-0.77698100
C 3.47618600,-2.76193700,-1.41331400
C 2.16091700,-2.75137000,-1.10920900
C 1.10586100,-2.54683600,-2.15618900
S 4.97188500,-1.07789800,1.86688500
O 4.14079900,-0.39135200,2.83948200
O 5.91406300,-2.09621600,2.29061800
C 5.88071700,0.15858400,0.93279500
C 7.22986400,-0.05992300,0.63577600
C 5.25103600,1.36660600,0.61424800
C 7.95088100,0.94988300,-0.01008200
H 7.70325800,-1.00200600,0.92528200
C 5.98813000,2.36647800,-0.02561700
H 4.20066300,1.51620600,0.88194000
C 7.34737500,2.17643000,-0.34424800
H 9.00754800,0.78687800,-0.24545000
H 5.50347100,3.31641100,-0.27304600
C 8.15514900,3.27741500,-0.99326800
H 7.50720300,4.01377500,-1.49302400
H 8.74857800,3.81599500,-0.23317200
H 8.86179300,2.87141500,-1.73470300
C -3.60867100,-1.99537400,-1.44855300
C -4.45845500,-1.19579000,-2.24261700
C -3.46410000,-3.35531800,-1.79982900
C -5.14675500,-1.74291400,-3.33783500
H -4.59503900,-0.13767300,-2.00828500
C -4.15747900,-3.90171700,-2.88513300
H -2.80214600,-3.99969800,-1.21438200
C -5.00271800,-3.09730400,-3.66084600
H -5.80760100,-1.10397400,-3.93116200
H -4.03358400,-4.96148100,-3.12578800
H -5.54600400,-3.52350100,-4.50894700
C -2.79025600,-2.50391200,1.26255100
C -3.93169700,-3.33049700,1.33340700
C -1.91766500,-2.47170300,2.36840300
C -4.17230600,-4.12033500,2.46341500
H -4.64635000,-3.36488100,0.50837900
C -2.16395800,-3.25862200,3.49918400
H -1.04173400,-1.82172900,2.36357600
C -3.28764000,-4.09113500,3.54791200
H -5.06075300,-4.75761600,2.49489000
H -1.47145900,-3.21534700,4.34452000
H -3.47735800,-4.70973000,4.42979900
C 1.11742600,2.11318200,-1.96684600
C 0.95842900,3.18114000,-2.87401700
C 2.41178500,1.56310300,-1.81237400
C 2.05161100,3.68870700,3.59019800
H -0.02283700,3.62813800,-3.04040700
C 3.50108700,2.07842300,-2.52128800
H 2.58452800,0.71688400,-1.14022300
C 3.32567800,3.14317300,-3.41573500

H 1.89696200,4.51631000,-4.28852300
H 4.49095500,1.63714300,-2.37607300
H 4.17738300,3.53837200,-3.97680600
C -1.69361600,2.46749700,-1.55764800
C -2.76142500,2.05465900,-2.37089100
C -1.69529500,3.78430400,-1.05514900
C -3.81554700,2.92759300,-2.66787400
H -2.77325200,1.03624200,-2.75837500
C -2.74276800,4.65968600,-1.35637400
H -0.87573600,4.12426700,-0.41403200
C -3.80870100,4.23173100,-2.15872700
H -4.64315000,2.58999300,-3.29866100
H -2.73262900,5.67601100,-0.95272300
H -4.63169100,4.91479600,-2.38739500
C -0.12919000,1.57085800,0.67312700
C -1.19435100,1.23235100,1.51740600
C 1.16798600,1.79637400,1.22555900
C -0.93995700,1.01823500,2.92378300
C 1.42782000,1.59248100,2.56142000
H 1.97933600,2.12025400,0.57206000
C 0.39187900,1.16513200,3.43657800
C -1.97105000,0.63404000,3.83986500
H 2.44071400,1.72226700,2.95573800
C 0.65547600,0.90701900,4.81542500
C -1.68695100,0.39587200,5.16886600
H -2.99492200,0.51105100,3.48094600
C -0.36109700,0.53081000,5.66640500
H 1.68212900,1.01399700,5.17848900
H -2.49151000,0.09772600,5.84724300
H -0.15332600,0.33733900,6.72259800
C -3.33610100,0.15864500,0.52253700
C -2.64962000,1.23058400,1.10412300
C -4.76954800,0.19324100,0.46912100
C -3.38963700,2.41402800,1.49757100
C -5.48134900,1.30697200,0.83752600
H -5.31567600,-0.69058900,0.13508800
C -4.80832300,2.46579100,1.31709600
C -2.74908400,3.56264400,2.05936000
H -6.57417100,1.30834600,0.77780400
C -5.52822800,3.64744800,1.65933600
C -3.46974800,4.69403000,2.38613900
H -1.67080700,3.55281500,2.22731200
C -4.87408000,4.74432700,2.17984500
H -6.61242000,3.66280300,1.51056600
H -2.95390000,5.55930500,2.81238200
H -5.43301300,5.64613000,2.44562200
P -2.50757100,-1.34533000,-0.13008100
P -0.32232500,1.34583700,-1.12911400
C 4.03787300,-2.93667400,-2.80380700
H 4.54737400,-2.01749500,-3.14587900
H 4.79972800,-3.73730300,-2.79846900
H 3.26866700,-3.20835200,-3.54043100
C 0.79476300,-3.77147700,0.83317700
H 0.73077200,-3.71025600,1.93313500
H -0.23718300,-3.78009000,0.46348800
H 1.27799800,-4.73049000,0.58076400

C 1.17606100,-1.34738600,-2.92658800
C -0.16505400,-3.18729800,-2.11362000
H -0.78426200,-3.16936900,-3.01685200
N 3.91113900,-1.71057600,0.73421900
C 2.78031400,-2.42012300,1.32196200
C 1.62318000,-2.57371600,0.30554600
C 0.81224900,-1.26448300,0.21697200
H 1.55824000,-0.46080700,0.14676200
H 0.25834900,-1.08774600,1.14934000
H 3.10824500,-3.42262600,1.66707100
H 2.41557400,-1.86036300,2.19608000
H 2.08541900,-0.74642100,-2.86957300
H 0.63951100,-1.30036800,-3.87966800
H -0.30040700,-4.05740800,-1.47115600
O -1.65768700,-0.94238100,-3.47532100
H -1.47615200,-0.24751100,-4.11824600
H -2.61067400,-1.11649800,-3.49924100
Rh -0.41484700,-1.10501100,-1.44815900

Ts9

Opt @ BMK/def2SVP in PhCl
SCF Done: E(BMK) = -3867.119908a.u.
Zero-point correction = 1.033062Hartree/Particle
Sum of electronic and thermal Free Energies = -3862.657198a.u.
Imaginary Frequency is -1157.76cm^{-1}

C 5.26606300,-1.96343900,-0.09122000
H 5.56304000,-2.87173600,0.47925700
H 6.19113200,-1.54222700,-0.52248700
C 4.34592000,-2.31437600,-1.24025100
C 3.00407100,-2.09906000,-1.14859100
C 2.13280800,-2.09074600,-2.35176800
S 5.64631100,-0.35872400,1.97636400
O 4.81922700,0.54666400,2.74686900
O 6.34230700,-1.45584100,2.62480200
C 6.85203600,0.58477000,1.04067800
C 8.15753400,0.09677200,0.92758500
C 6.48234600,1.82047900,0.49655900
C 9.10385400,0.85687400,0.23153600
H 8.42096400,-0.85799400,1.39088000
C 7.44111000,2.56364600,-0.19496700
H 5.46252100,2.19072100,0.62855500
C 8.76335100,2.09662500,-0.33827000
H 10.12807500,0.48232900,0.13738900
H 7.16523700,3.53201900,-0.62458000
C 9.80155700,2.93057800,-1.05374300
H 10.59257200,2.30044900,-1.48916900
H 9.34960300,3.53113200,-1.85881300
H 10.28412700,3.63010700,-0.34792900
C -2.11122300,-3.33059000,-0.98659600
C -2.19610100,-3.40783800,-2.39462300
C -2.07258700,-4.53525200,-0.25898400
C -2.28846600,-4.63842500,-3.04794300
H -2.18097500,-2.49325400,-2.99538200
C -2.14898600,-5.77213500,-0.91635700
H -1.97656300,-4.51934000,0.82846100

C -2.26770200,-5.82894600,-2.30747800
H -2.36584300,-4.66948500,-4.13853300
H -2.11673400,-6.69419300,-0.32899100
H -2.33416600,-6.79454400,-2.81680900
C -2.07465500,-2.06886000,1.58784200
C -3.27943900,-2.65266300,2.03546600
C -1.07149400,-1.78106700,2.52610400
C -3.46139600,-2.95495700,3.38639800
H -4.08074800,-2.87059300,1.32201400
C -1.25695000,-2.08103400,3.88200300
H -0.14775500,-1.30074900,2.20858900
C -2.44899900,-2.66967900,4.31423800
H -4.39963700,-3.40818300,3.71866400
H -0.46673300,-1.84496100,4.60034700
H -2.59392400,-2.90387200,5.37272900
C 0.26415300,3.04104500,-0.31270000
C -0.13283700,4.18769100,0.41241600
C 1.52475600,3.04242300,-0.93878500
C 0.70317100,5.30370600,0.49650000
H -1.09262800,4.20750900,0.93561200
C 2.35825300,4.16720300,-0.85484900
H 1.85245200,2.16567200,-1.50179100
C 1.95175800,5.29802300,-0.14141100
H 0.37990100,6.18031400,1.06519500
H 3.33159000,4.15234000,-1.35301300
H 2.60473000,6.17312700,-0.07524000
C -2.08616900,2.24450600,-1.73988900
C -2.34799500,1.45655600,-2.87830400
C -2.73506500,3.48796700,-1.62114600
C -3.24118700,1.88759100,-3.86518600
H -1.86742600,0.47722500,-2.99752400
C -3.63132000,3.91934100,-2.60381200
H -2.54661100,4.12809100,-0.75619800
C -3.88756600,3.12126400,-3.72632500
H -3.43235300,1.25891400,-4.73926700
H -4.13148800,4.88537000,-2.49219900
H -4.58661300,3.46382000,-4.49456100
C -1.66189900,1.45734900,1.11207700
C -2.93265500,0.90528400,1.28087000
C -0.86789500,1.77206000,2.26418300
C -3.48440800,0.76429800,2.60873900
C -1.37082800,1.62056900,3.53459000
H 0.14776700,2.15287300,2.13542500
C -2.69704000,1.14561300,3.74266500
C -4.79981000,0.25836600,2.84429600
H -0.75415800,1.87789800,4.40154500
C -3.24263100,1.03022400,5.05431300
C -5.30746700,0.16830400,4.12505600
H -5.41557000,-0.05516600,1.99936400
C -4.52499800,0.55884600,5.24421200
H -2.62602600,1.32952700,5.90762700
H -6.32188800,-0.21034300,4.27978900
H -4.94110600,0.48340100,6.25290200
C -3.45010700,-0.76423200,-0.55806600
C -3.75265700,0.43109900,0.11323200
C -4.33735500,-1.21841700,-1.58378900

C -4.89986200,1.21164000,-0.27529900
C -5.43580300,-0.48192900,-1.96672900
H -4.16942200,-2.18075700,-2.06419100
C -5.73539800,0.76022000,-1.34608700
C -5.23430300,2.44635600,0.36422200
H -6.09859600,-0.85961800,-2.75167000
C -6.85612400,1.54480600,-1.75038400
C -6.32704800,3.18322700,-0.04404000
H -4.61532400,2.81132400,1.18650900
C -7.14772200,2.73257000,-1.11438300
H -7.48379500,1.18159300,-2.57009100
H -6.56613400,4.12349400,0.46116400
H -8.01168700,3.32713800,-1.42480500
P -1.89264000,-1.69865100,-0.18442100
P -0.87196600,1.62818900,-0.52563100
C 5.08490900,-2.92926000,-2.40639400
H 5.72914900,-2.18758600,-2.91193200
H 5.76007600,-3.71144600,-2.01375200
H 4.42064100,-3.38869400,-3.14889600
C 1.56253800,-3.02794200,0.75360100
H 1.41388500,-2.91961700,1.84052500
H 0.57127100,-3.17900000,0.31123600
H 2.15516300,-3.94371100,0.59336800
C 2.59515800,-1.49360900,-3.55316200
C 0.72207500,-2.36230900,-2.22251300
H 0.21167100,-2.43337300,-3.19527800
N 4.63853500,-0.97368100,0.77029800
C 3.40245600,-1.52223500,1.31011600
C 2.33037600,-1.79059500,0.20582200
C 1.44088000,-0.52832600,0.08232900
H 2.08360300,0.31932400,-0.21195500
H 0.99014100,-0.28173300,1.05768400
H 3.62666600,-2.47380600,1.83433200
H 2.99368000,-0.82170800,2.05297600
H 0.46361800,-3.20133800,-1.57552500
O 1.15643100,0.46276800,-2.67814900
H 1.95624500,-0.36909300,-3.23643400
H 0.78636700,1.18512600,-3.19922000
Rh -0.04092800,-0.51342600,-1.32889200
H 3.65276000,-1.24013400,-3.65653200
H 2.08175600,-1.77466600,-4.48218500

Int9
Opt @ BMK/def2SVP in PhCl
SCF Done: E(BMK) = -3867.135667a.u.
Zero-point correction = 1.040999Hartree/Particle
Sum of electronic and thermal Free Energies = -3862.665348a.u.

C -4.89578600,2.12737600,0.16616400
H -5.40994200,2.90539700,0.77390600
H -5.67923600,1.60766300,-0.41481100
C -3.95999200,2.78172600,-0.83110300
C -2.60206000,2.72911200,-0.68853400
C -1.66174100,3.05553300,-1.79311000
S -5.17623500,0.35573200,2.11559200
O -4.27670400,-0.49958500,2.86274200

O -6.02546700,1.31329500,2.80014000
C -6.22084600,-0.66095000,1.06966400
C -7.56162000,-0.30326000,0.89045700
C -5.69970500,-1.83895500,0.52316000
C -8.38593300,-1.13457500,0.12531000
H -7.94910200,0.60488800,1.36002200
C -6.54079100,-2.65799100,-0.23429000
H -4.65579000,-2.11214400,0.70146300
C -7.89290200,-2.32262300,-0.44483000
H -9.43582900,-0.86114000,-0.02035800
H -6.14535100,-3.58724800,-0.65743100
C -8.80606700,-3.23791200,-1.22789400
H -9.56985400,-2.66648000,-1.77841500
H -8.24242200,-3.85180700,-1.94745900
H -9.33565000,-3.92592100,-0.54489800
C 2.77050400,2.86166200,-1.15654700
C 2.87593100,2.56363400,-2.53453400
C 2.90817700,4.19797300,-0.74390400
C 3.13658400,3.57082500,-3.46709000
H 2.76175300,1.52996100,-2.88327200
C 3.16111700,5.20905100,-1.68355300
H 2.80962100,4.46583500,0.31133600
C 3.27964300,4.90011600,-3.04230900
H 3.22515100,3.31887400,-4.52773100
H 3.26562600,6.24388000,-1.34503200
H 3.48087500,5.69088600,-3.77066200
C 2.56119100,2.09712600,1.64831000
C 3.83097600,2.62907900,1.96281400
C 1.62828300,1.91626400,2.68388100
C 4.13680700,3.00764400,3.27265000
H 4.58651200,2.74684400,1.17941700
C 1.93807000,2.29054100,3.99799500
H 0.65844100,1.46462300,2.47413600
C 3.18754200,2.84432800,4.29231300
H 5.12102400,3.42764400,3.49937700
H 1.20054000,2.14048800,4.79127100
H 3.42867400,3.14109100,5.31720100
C -0.91641000,-2.44985000,-1.27250900
C -0.71782900,-3.84767100,-1.24883300
C -2.20513800,-1.95747800,-1.55514500
C -1.77298200,-4.72481400,-1.50832300
H 0.26954000,-4.26170800,-1.03044800
C -3.25889200,-2.84148800,-1.83208500
H -2.39784700,-0.88335200,-1.58180300
C -3.04698100,-4.22275300,-1.81037900
H -1.59734900,-5.80402400,-1.48510000
H -4.24738800,-2.43651000,-2.06674500
H -3.86696900,-4.91177900,-2.03356400
C 1.75885300,-1.99124600,-2.12498500
C 1.88150800,-1.35419100,-3.37666100
C 2.53499100,-3.13420300,-1.85938900
C 2.77304400,-1.84885100,-4.33669600
H 1.25945200,-0.48341500,-3.61201700
C 3.42933500,-3.62118600,-2.81792300
H 2.46179100,-3.63807200,-0.89221900
C 3.55063500,-2.97903600,-4.05736700

H 2.85513800,-1.35033200,-5.30673000
H 4.03585700,-4.50281700,-2.59117300
H 4.24939600,-3.36250300,-4.80649600
C 0.97770900,-1.65975000,0.76981800
C 2.22183100,-1.31015000,1.30957900
C -0.08939900,-2.04470900,1.64606200
C 2.40543700,-1.30714800,2.74072500
C 0.07960600,-2.06455900,3.01071600
H -1.06029500,-2.31895900,1.22924000
C 1.32023500,-1.68543600,3.59593400
C 3.63720700,-0.90896800,3.34812400
H -0.75164400,-2.35967500,3.65848700
C 1.49706400,-1.66862700,5.01148700
C 3.77718400,-0.89622000,4.72032400
H 4.47208000,-0.59477600,2.71827700
C 2.70011200,-1.28399600,5.56418500
H 0.65872800,-1.96674700,5.64878500
H 4.72610600,-0.58262300,5.16451400
H 2.82945400,-1.27497400,6.65023800
C 3.53088100,0.23739400,-0.22505800
C 3.41003700,-0.98029400,0.44983000
C 4.71630900,0.50208800,-0.98249000
C 4.46074600,-1.96532000,0.35964200
C 5.71930900,-0.43293900,-1.08928400
H 4.84822100,1.46292100,-1.47916600
C 5.61806000,-1.69148600,-0.43587500
C 4.38246400,-3.23511400,1.01394200
H 6.61479700,-0.20838800,-1.67709900
C 6.64505700,-2.67382900,-0.55808900
C 5.38877600,-4.16969900,0.87530100
H 3.51300900,-3.47357900,1.62992900
C 6.53519300,-3.89004300,0.08152300
H7.52266600,-2.44220500,-1.16963100
H 5.30634500,-5.13381300,1.38537900
H 7.32670600,-4.63866200,-0.01472000
P 2.22055000,1.52252000,-0.04862700
P 0.51505500,-1.35020000,-0.96790800
C -4.75307900,3.47758900,-1.91733500
H -5.02237700,2.78275800,-2.73198900
H -5.70121000,3.83881200,-1.48359500
H -4.23980900,4.34357500,-2.34960000
C -1.15884000,3.30177700,1.37615500
H -1.18756700,3.09720300,2.45979200
H -0.09675600,3.33898600,1.10934800
H -1.59859400,4.30046700,1.21846200
C -2.12731700,3.07551000,-3.22912300
C -0.31176600,3.27685100,-1.59182300
H 0.29374600,3.53116500,-2.46718000
N -4.19992000,1.17145800,1.00712000
C -3.07871600,1.84237400,1.64770600
C -1.97332800,2.20820400,0.62260000
C -1.16486800,0.91462400,0.34573500
H -1.89852200,0.12111000,0.13057600
H -0.62166300,0.61356500,1.25391200
H -3.43302300,2.76514700,2.15245500
H -2.64880500,1.18129400,2.41421000

H 0.08038800,3.59963800,-0.63438400
O -0.96372100,0.58322100,-2.86139500
H -1.27782500,2.87588000,-3.89400800
H -1.13163800,-0.34029900,-3.06927300
Rh 0.10424900,0.95669400,-1.26217100
H -2.90021100,2.32482000,-3.42946300
H -2.51841100,4.07869100,-3.47095900

Ts10

Opt @ BMK/def2SVP in PhCl
SCF Done: E(BMK) = -3867.111628a.u.
Zero-point correction = 1.034097Hartree/Particle
Sum of electronic and thermal Free Energies = -3862.643819a.u.
Imaginary Frequency is -1156.04cm^{-1}

C 4.99774500,-1.45633700,-0.35919200
H 5.97035300,-1.95190200,-0.14407300
H 5.20475100,-0.68516900,-1.12232500
C 4.04800600,-2.46268900,-0.98313700
C 2.90864900,-2.87292400,-0.34884100
C 1.82162900,-3.62928000,-1.02900600
S 5.38951300,0.38421300,1.50353900
O 4.81224800,0.64644200,2.80510300
O 6.79433400,0.06136500,1.35062300
C 5.04667200,1.79131800,0.43764600
C 6.05111200,2.28047400,-0.40069600
C 3.79519900,2.41525200,0.51432100
C 5.78292700,3.40404200,-1.19379200
H 7.02847800,1.78985700,-0.41397500
C 3.54091700,3.52820500,-0.28873100
H 3.04214200,2.02805400,1.20869000
C 4.53140000,4.04339500,-1.15207300
H 6.56665000,3.79596200,-1.84984600
H 2.55959300,4.01374000,-0.24101200
C 4.26648700,5.28164900,-1.97757900
H 4.91595100,5.31996400,-2.86557300
H 3.21763400,5.32735600,-2.31021500
H 4.46575600,6.18921900,-1.38020700
Rh -0.33255800,-1.86958000,0.48516100
C -2.03935600,-0.99407900,3.50447100
C -1.02325700,-1.60262700,4.26717000
C -3.31275700,-0.82660500,4.08471800
C -1.27236100,-2.03894800,5.57342900
H -0.02131100,-1.72238000,3.84459200
C -3.56225700,-1.26978500,5.38836700
H -4.12149000,-0.35451700,3.51950600
C -2.54442600,-1.87745200,6.13464100
H -0.47051700,-2.50464400,6.15331400
H -4.55700000,-1.13698700,5.82337700
H -2.74242100,-2.22201800,7.15363700
C -1.15782200,1.38066600,2.23993600
C -2.05111100,2.21081900,2.94526400
C 0.11158800,1.87943700,1.89754100
C -1.68678600,3.51643100,3.28447300
H -3.03884100,1.83956600,3.23288000
C 0.47928500,3.18424100,2.24799000

H 0.81621000,1.24355700,1.35503100
C -0.41975700,4.00511200,2.93819900
H -2.39159300,4.15209300,3.82752100
H 1.47518800,3.55884800,1.99182200
H -0.13044200,5.02260800,3.21595300
C 1.04455100,-0.24419700,-2.43022600
C 1.15788300,-0.78146000,-3.72775200
C 2.15199700,0.43815900,-1.88651200
C 2.32611600,-0.58725500,-4.47769900
H 0.33016800,-1.34125600,-4.16802000
C 3.30777600,0.65233500,-2.64439900
H 2.10599400,0.85162400,-0.87610800
C 3.39443400,0.14545700,-3.94771600
H 2.38972900,-0.99869000,-5.48945000
H 4.13102600,1.23023100,-2.21332400
H 4.29332700,0.31868300,-4.54664100
C -1.70458200,-1.14514800,-2.53941300
C -2.35249600,-2.34683300,-2.20162200
C -2.06904000,-0.48695400,-3.73188600
C -3.33410800,-2.88799400,-3.04130900
H -2.11767500,-2.86719900,-1.26751000
C -3.04073100,-1.03285000,-4.57442600
H -1.59308200,0.46213600,-3.99979900
C -3.67531700,-2.23513800,-4.23039100
H -3.83344200,-3.81941400,-2.76040800
H -3.31177400,-0.51397400,-5.49827500
H -4.44039400,-2.65811500,-4.88767700
C -1.09690900,1.31232200,-1.19399700
C -2.34070600,1.54593000,-0.58572600
C -0.29045200,2.43142000,-1.57133300
C -2.75932000,2.89324800,-0.29314600
C -0.68164600,3.72336300,-1.30042900
H 0.65066700,2.27176500,-2.09683300
C -1.90725600,3.99173600,-0.63392400
C -4.00230600,3.18109300,0.35267700
H -0.04975000,4.56242500,-1.60798700
C -2.30695500,5.32372500,-0.31627000
C -4.36430000,4.47933700,0.64789800
H -4.67262100,2.36117200,0.61869400
C -3.50911200,5.56515100,0.31351300
H -1.64268500,6.15064600,-0.58598400
H -5.31978300,4.67666500,1.14243300
H -3.81132000,6.58886500,0.55207100
C -3.09859600,-0.41544400,0.84301300
C -3.29545400,0.42908800,-0.25440000
C -3.97405700,-1.53390200,1.04770100
C -4.44937900,0.24053400,-1.10140000
C -5.06404400,-1.73584900,0.23435200
H -3.77257500,-2.23510300,1.86112300
C -5.34932100,-0.84320800,-0.83779800
C -4.74248100,1.10473500,-2.20142700
H -5.73027200,-2.58588600,0.41187800
C -6.50332800,-1.01912600,-1.65579500
C -5.87298900,0.91617000,-2.97091000
H -4.06598200,1.92981700,-2.43254400
C -6.76629400,-0.15427900,-2.69748900

H -7.18080700,-1.85018000,-1.43619000
H -6.08317500,1.59749800,-3.80032200
H -7.65960900,-0.28900500,-3.31400800
P -1.59136200,-0.34371500,1.86686000
P -0.46751100,-0.40711800,-1.42884500
C 4.54298100,-2.86550300,-2.36133700
H 3.84508700,-2.55556900,-3.15414800
H 5.50843200,-2.37879100,-2.56924500
H 4.69951300,-3.95003300,-2.44784000
C 2.44660500,-3.74534300,1.98998800
H 2.23210100,-3.45083800,3.03367100
H 1.65140600,-4.41300300,1.64310000
H 3.37729700,-4.33570300,2.00344200
N 4.45102500,-0.82414500,0.82378100
C 3.90676600,-1.81603300,1.73235800
C 2.64578300,-2.47872000,1.11765600
C 1.51028600,-1.43437200,1.24174800
H 1.86032600,-0.51322400,0.75225300
H 1.35941000,-1.20776200,2.31249300
H 4.66626000,-2.59615000,1.94167300
H 3.64935300,-1.33314600,2.68537100
C 0.50679400,-3.52599800,-0.62619800
C 2.12039500,-4.53721600,-2.20578100
H 0.12945700,-3.78220400,0.61230000
H -0.19219900,-4.07533200,-1.27618200
H 3.02557300,-5.13874200,-2.03672500
H 1.27428500,-5.21811500,-2.37537200
H 2.27136600,-3.95691400,-3.13086500
O -0.61534800,-3.52300800,1.67167800
H -0.17126300,-3.57137300,2.52518900

Int10
Opt @ BMK/def2SVP in PhCl
SCF Done: E(BMK) = -3867.140224a.u.
Zero-point correction = 1.037247Hartree/Particle
Sum of electronic and thermal Free Energies = -3862.676348a.u.

C -4.73051400,1.95840600,-0.29070600
H -5.41065600,2.73790900,0.11895100
H -5.35046700,1.27186000,-0.89160100
C -3.71476300,2.62626900,-1.20460700
C -2.43188300,2.83384000,-0.82076200
C -1.36873100,3.48265400,-1.66532500
S -5.19938800,0.59070900,1.93141400
O -4.39893300,-0.15570200,2.88145200
O -6.05594900,1.67581000,2.37276500
C -6.22840000,-0.56963900,1.02415900
C -7.40835800,-0.11739400,0.41764300
C -5.90993700,-1.93017600,1.06565500
C -8.24977900,-1.04485500,-0.20014600
H -7.67090200,0.94336600,0.45774000
C -6.77185500,-2.84658000,0.45150600
H -5.01436300,-2.26151200,1.59842000
C -7.94790700,-2.42144000,-0.19254100
H -9.17170300,-0.69789900,-0.67794300
H -6.53522300,-3.91477600,0.49051500

C -8.89653600,-3.41462100,-0.82373300
H -8.42462700,-4.40084400,-0.95005800
H -9.79048400,-3.54796500,-0.18930400
H -9.24317000,-3.06332000,-1.80924300
Rh 0.20336200,0.93410700,-1.26807100
C 2.96312600,2.55171400,-1.62927500
C 3.03374300,2.00596500,-2.92985800
C 3.17368000,3.93085500,-1.46261600
C 3.32681800,2.81484800,-4.03126700
H 2.87522200,0.93233200,-3.08155800
C 3.46871200,4.74057400,-2.56911900
H 3.09515900,4.38833300,-0.47336400
C 3.54672000,4.18804800,-3.85190900
H 3.38374100,2.37381100,-5.03045300
H 3.63410700,5.81179900,-2.42251100
H 3.77603800,4.82402900,-4.71165300
C 2.72269400,2.33984900,1.25856400
C 4.04499300,2.79848400,1.45625200
C 1.80055700,2.46239600,2.30848800
C 4.41308200,3.39997400,2.66165300
H 4.79218100,2.68606700,0.66441900
C 2.17213700,3.06409700,3.51873300
H 0.79123500,2.07475100,2.19594500
C 3.47368200,3.54051400,3.69459300
H 5.43749800,3.75886100,2.79655000
H 1.43922400,3.15059600,4.32563900
H 3.76343700,4.01357700,4.63738600
C -0.96253200,-2.55033400,-1.30574200
C -0.87603600,-3.59590600,-2.25129100
C -2.25675100,-2.17049900,-0.86799500
C -2.02381900,-4.23771400,-2.73375900
H 0.09544600,-3.91666100,-2.62921300
C -3.40146100,-2.81087400,-1.35990900
H -2.40078000,-1.37715000,-0.12946600
C -3.29140400,-3.84694900,-2.29527900
H -1.91710400,-5.04543400,-3.46361600
H -4.38456400,-2.49375000,-1.00166100
H -4.18794500,-4.34210800,-2.67866700
C 1.85479400,-2.53783500,-1.67121400
C 2.40579900,-2.06081300,-2.87328800
C 2.27597200,-3.78581800,-1.17233100
C 3.37153100,-2.80786200,-3.56009200
H 2.06572500,-1.10522900,-3.28753900
C 3.24173900,-4.53039700,-1.85560500
H 1.85222800,-4.17247900,-0.24008600
C 3.79247200,-4.04126200,-3.04889200
H 3.79188800,-2.42716000,-4.49515800
H 3.56733700,-5.49442500,-1.45454300
H 4.54737700,-4.62588400,-3.58258000
C 0.81948200,-1.67241300,0.94068900
C 2.02210600,-1.17634300,1.46604600
C -0.25913000,-1.98972300,1.82066200
C 2.14045900,-0.94234100,2.88198100
C -0.15747700,-1.77413700,3.17739600
H -1.17921600,-2.41800900,1.42021500
C 1.02812700,-1.22612900,3.74119900

C 3.33870200,-0.43013500,3.47164500
H -0.99701400,-2.02170200,3.83425700
C 1.13862900,-0.97775300,5.14251700
C 3.41565200,-0.20200800,4.82945700
H 4.19688300,-0.20228700,2.83494400
C 2.30612600,-0.47659400,5.67703200
H 0.27997700,-1.19901800,5.78407200
H 4.33920400,0.19506100,5.26015900
H 2.38505300,-0.29336900,6.75241700
C 3.50524600,0.07784600,-0.21609300
C 3.25847400,-1.01854400,0.61604800
C 4.72994900,0.13924700,-0.95532800
C 4.23237600,-2.08140800,0.70699300
C 5.65158600,-0.87932000,-0.89855700
H 4.95026600,1.00761700,-1.57654900
C 5.42628100,-2.01975100,-0.08034300
C 4.04010600,-3.22587800,1.54274000
H 6.57662100,-0.81412300,-1.47985000
C 6.36900200,-3.08830100,-0.02622700
C 4.97077700,-4.24455500,1.57905600
H 3.14303100,-3.29779100,2.16066400
C 6.14774600,-4.18131000,0.78461200
H 7.27467000,-3.02046800,-0.63695400
H 4.80288500,-5.10815100,2.22910000
H 6.87714900,-4.99533200,0.82547800
P 2.32683900,1.48539800,-0.29826400
P 0.53924500,-1.58830900,-0.86052600
C -4.29633700,3.05499400,-2.53857800
H -4.99086700,2.28911100,-2.92441300
H -4.88070800,3.98805200,-2.43518800
H -3.52281400,3.22535000,-3.30101500
C -1.18393700,3.54353400,1.24456900
H -1.01692000,3.30605400,2.30857300
H -0.21525500,3.76513100,0.77629800
H -1.80393900,4.45499300,1.20605600
C -1.59554700,4.90092900,-2.15933800
C -0.18421500,2.84857000,-1.79933500
H 0.59330600,3.36904100,-2.38127000
N -4.09962100,1.19669500,0.78776400
C -3.08970600,2.01364300,1.47267400
C -1.91089300,2.37401500,0.54369500
C -1.02286900,1.11613400,0.36901100
H -1.71350800,0.27195100,0.22862700
H -0.44347200,0.90897700,1.28288400
H -3.55289200,2.95087700,1.84391300
H -2.71665600,1.45238000,2.34225900
H -0.72907500,5.25540200,-2.73909600
H -1.73238100,5.57933000,-1.29708800
H -2.49568900,5.00451400,-2.78499300
O -1.50594900,0.46116400,-2.50866400
H -1.96952500,-0.36771200,-2.30927100
H -2.14716600,1.18513000,-2.39120200

Int11
Opt @ BMK/def2SVP in PhCl
SCF Done: E(BMK) = -3867.118392a.u.

Zero-point correction = 1.033763Hartree/Particle
Sum of electronic and thermal Free Energies = -3862.653733a.u.

N -5.15380900,0.76657800,-0.29257400
C -5.19524400,1.97826800,0.51155600
H -6.18119200,2.45228300,0.36852700
H -5.11244100,1.71722000,1.58191100
C -4.16370100,3.04051400,0.14799100
C -4.25009900,-0.31120700,0.11990700
H -4.60340300,-1.24572400,-0.34395300
H -4.31459600,-0.43490400,1.21206900
C -2.81624600,-0.05672100,-0.32810300
C -1.79985000,0.21036400,0.51508900
C -1.66113700,0.37597300,1.98333700
C -2.67212900,0.24073300 3.09336800
C -0.34268200,0.65033400 2.08210600
C -3.54983500,3.74806500 1.11530100
S -6.38868700,0.45484200,-1.34954200
O -6.86010700,1.74686000,-1.80888700
O -5.89384100,-0.55290900,-2.26673600
C -7.72312800,-0.29596900,-0.40131800
C -7.76007700,-1.68703500,-0.24678600
C -8.70467200,0.52203800,0.17302200
C -8.78042300,-2.25748100,0.52070400
H -7.01448400,-2.31185200,-0.74581300
C -9.71619200,-0.06701600,0.93686700
H -8.68836000,1.60112600,-0.00225900
C -9.77030800,-1.46105000,1.12544900
H -8.81687200,-3.34498900,0.64073200
H -10.48794200,0.56756500,1.38446400
C -10.89366800,-2.08775000,1.91994200
H -10.60343200,-3.07146400,2.32033000
H -11.19712000,-1.44386600,2.76064500
H -11.78136000,-2.23634100,1.27947200
H -2.93100900,4.62061500,0.87526800
H -3.71408700,3.53199400,2.17711500
C -3.98745200,3.31344000,-1.32298400
H -3.52843800,2.43899400,-1.81644200
H -4.97124700,3.45385500,-1.80306100
H -3.35881600,4.19924700,-1.50147000
C -2.59525400,-0.15509600,-1.82663700
H -2.28756300,-1.18164500,-2.10653200
H -3.52043100,0.06134100,-2.38451300
H -1.80561400,0.53706400,-2.16780600
Rh 0.12635700,0.76287700,0.15856700
C -0.36658900,-2.68221600,0.68324000
C -0.89699200,-2.68782300,1.99332400
C -0.91308500,-3.58555300,-0.25181800
C -1.89951500,-3.58994100,2.36135700
H -0.54140400,-1.97368400,2.73539800
C -1.92638400,-4.48035100,0.11722600
H -0.56297800,-3.60013800,-1.28461800
C -2.41830200,-4.49318000,1.42495700
H -2.27765200,-3.58391400,3.38795500
H -2.32773500,-5.17079000,-0.63008600
H -3.20295800,-5.19833600,1.71392000

C 1.29292300,-1.93411800,-1.53799000
C 1.90552600,-3.17391800,-1.80465300
C 0.98086000,-1.08343400,-2.61322000
C 2.20329900,-3.54805200,-3.11764000
H 2.15584600,-3.84823300,-0.97924000
C 1.28432100,-1.45382400,-3.92873900
H 0.48936800,-0.11978000,-2.43268000
C 1.89685600,-2.68720700,-4.18067500
H 2.68447500,-4.51075300,-3.31162200
H 1.03741100,-0.78160500,-4.75495600
H 2.13267400,-2.98087700,-5.20753800
C 2.31177700,3.30072000,-0.92433300
C 3.00492400,4.46574600,-0.54292100
C 1.45016800,3.36504700,-2.04224200
C 2.84697000,5.65648400,-1.26586700
H 3.67280100,4.44870600,0.32196700
C 1.30159200,4.55044000,-2.76897300
H 0.89325300,2.47560600,-2.35841500
C 2.00061400,5.70180400,-2.37932900
H 3.39300400,6.55181600,-0.95493200
H 0.63611400,4.57660800,-3.63658700
H 1.88182700,6.63212300,-2.94227700
C 3.33936500,2.17000100,1.48800800
C 2.59662200,2.58572700,2.61048000
C 4.74440500,2.21305000,1.54363000
C 3.24740600,3.02698200,3.76835000
H 1.50346700,2.57605300,2.57568000
C 5.39331800,2.64627900,2.70540900
H 5.33513700,1.90904500,0.67559000
C 4.64701300,3.05224000,3.81903700
H 2.66003200,3.34939700,4.63283900
H 6.48627800,2.66791200,2.73897200
H 5.15641700,3.39121200,4.72575300
C 3.56451700,0.68726800,-1.04197000
C 3.91991600,-0.60577200,-0.63715900
C 3.94469300,1.14901600,-2.34176600
C 4.65488500,-1.45950600,-1.54018300
C 4.62271300,0.33548100,-3.21944000
H 3.69665300,2.16562600,-2.64974400
C 4.98175600,-0.99020400,-2.85296300
C 5.06047100,-2.78423600,-1.18364700
H 4.90077200,0.70900600,-4.20992700
C 5.67490700,-1.84537700,-3.76077500
C 5.73594700,-3.58763600,-2.07922200
H 4.82899100,-3.16966300,-0.18895100
C 6.04566400,-3.11877000,-3.38508000
H 5.91481700,-1.46589800,-4.75894800
H 6.03808300,-4.59574800,-1.78081600
H 6.58354900,-3.76622300,-4.08349300
C 2.39874600,-1.65679700,1.15351500
C 3.64315800,-1.14853200,0.74439400
C 2.28633700,-2.26214800,2.44524800
C 4.77574300,-1.22570200,1.64128500
C 3.35184900,-2.32349700,3.31202500
H 1.34669000,-2.71567500,2.75259500
C 4.61879500,-1.79789600,2.94449900

C 6.07919200,-0.76061900,1.27488000
H 3.23317800,-2.79708600,4.29148000
C 5.73155100,-1.86428000,3.83537900
C 7.14025900,-0.84325900,2.15261100
H 6.24303400,-0.33783300,0.28250600
C 6.96880100,-1.39580800,3.45140600
H 5.58319200,-2.30356100,4.82670700
H 8.12636500,-0.48442600,1.84373100
H 7.81973300,-1.45521300,4.13585600
P 0.86946700,-1.43823700,0.15463300
P 2.44606100,1.71597100,-0.02257500
H -3.50398400,0.95479300,2.96736200
H -3.10686200,-0.77491100,3.10506600
H -2.20227900,0.43190200,4.07163900
H 0.29313300,0.80294700,2.96319800
O -0.78484400,2.72543000,-0.08393500
H -1.66921400,2.81614800,0.32374300
H -0.29519900,3.54865600,0.03420500

Ts11

Opt @ BMK/def2SVP in PhCl

SCF Done: E(BMK) = -3867.086254a.u.

Zero-point correction = 1.029140Hartree/Particle

Sum of electronic and thermal Free Energies = -3862.62424a.u.

Imaginary Frequency is -1308.66cm^{-1}

N -5.28266200,0.63096600,-0.08837300
C -5.86851200,1.94443300,0.15845200
H -6.85736400,1.96143400,-0.33250700
H -6.04979200,2.06982900,1.24230800
C -5.07799900,3.12778100,-0.37588700
C -4.09376300,0.20963600,0.63788600
H -4.18816800,-0.86702800,0.86499400
H -4.05531200,0.76276400,1.59187100
C -2.77891700,0.38844400,-0.12682200
C -1.69998300,0.87468200,0.50052800
C -1.38594900,1.54536400,1.73488300
C -1.78535500,2.87480900,2.01136500
C -0.06354000,1.02971000,2.16113400
C -4.89008300,4.20905100,0.39574600
S -6.14394300,-0.45466700,-0.99125800
O -6.45900200,0.17208900,-2.26074000
O -5.39890000,-1.69935100,-0.93028200
C -7.70660800,-0.69669300,-0.12660100
C -7.73003900,-1.47756700,1.03688300
C -8.87536800,-0.12428200,-0.63712300
C -8.94204400,-1.66288200,1.70414800
H -6.81272200,-1.94568600,1.40426800
C -10.08192600,-0.32265400,0.04469100
H -8.83549100,0.45489800,-1.56353300
C -10.13604400,-1.09012100,1.22081800
H -8.96755000,-2.27182800,2.61368800
H -10.99957700,0.12394400,-0.35094700
C -11.44634200,-1.33024900,1.93553400
H -11.31118900,-1.33022100,3.02899100
H -12.19220800,-0.56331400,1.67633000

H -11.86418300,-2.31311900,1.65350900
H -4.40136900,5.10889400,0.00786100
H -5.23990000,4.23845300,1.43367800
C -4.61989700,3.04492600,-1.81177000
H -3.72842900,2.39958000,-1.89277200
H -5.39870100,2.59074600,-2.44800100
H -4.35913400,4.03987800,-2.20399800
C -2.73692700,-0.17771000,-1.52397200
H -2.91931300,-1.26678500,-1.49012900
H -3.53820200,0.24528000,-2.15275200
H -1.76758100,0.01410800,-2.00930200
Rh 0.34243500,1.11035400,0.13429000
C -0.68798700,-2.38638600,0.20390300
C -1.52265600,-2.22785500,1.33242100
C -1.02749900,-3.38424300,-0.7318970
C -2.63154700,-3.05452600,1.53157200
H -1.32543100,-1.44505900,2.06582800
C -2.14950600,-4.20082600,-0.53879300
H -0.43259600,-3.52236800,-1.63547600
C -2.95434600,-4.04183800,0.59172000
H -3.25204600,-2.91747400,2.42186000
H -2.39614800,-4.95843600,-1.28766500
H -3.83420800,-4.67456800,0.73538700
C 1.29147400, -1.68232700,-1.73756600
C 1.92862700,-2.90805100,-2.00891700
C 1.02241800,-0.80570900,-2.80392200
C 2.29930500,-3.24009600,-3.31516500
H 2.14166800,-3.60555400,-1.19319800
C 1.39455100,-1.13465900,-4.11294000
H 0.50195400,0.14133300,-2.61747500
C 2.03554400,-2.35280500,-4.36794800
H 2.79998100,-4.19279900,-3.51036700
H 1.17778200,-0.44371300,-4.93219200
H 2.32588400,-2.61420600,-5.38959700
C 2.82061300,3.04745300,-1.25815100
C 3.71601800,4.11921600,-1.09000900
C 1.93388200,3.07174600,-2.35481300
C 3.72506100,5.18484300,-2.00044400
H 4.40906100,4.12916500,-0.24443500
C 1.94413600,4.13430000,-3.26479100
H 1.22987600,2.24542000,-2.50922000
C 2.84273700,5.19565300,-3.08705000
H 4.42559400,6.01226800,-1.85523800
H 1.24969200,4.13552700,-4.10980600
H 2.85092500,6.03068800,-3.79347700
C 3.55979500,2.26163300,1.38935700
C 2.84007800,3.15589500,2.21049000
C 4.89171500,1.95102400,1.71131200
C 3.44600700,3.71225700,3.34112000
H 1.81724800,3.43273000,1.93626600
C 5.49088300,2.50609000,2.84962100
H 5.46752900,1.28099600,1.07012100
C 4.76943200,3.38274300,3.66731700
H 2.88384200,4.40934500,3.96929700
H 6.52654700,2.25268700,3.09300800
H 5.23929500,3.81605100,4.55503500

C 3.85603100,0.38407000,-0.85826100
C 3.98255800,-0.91126000,-0.34117600
C 4.51958300,0.72339300,-2.08157500
C 4.76375000,-1.89142000,-1.06101100
C 5.26166300,-0.20182400,-2.77563700
H 4.43492700,1.73602900,-2.47737100
C 5.39214800,-1.53348900,-2.29655300
C 4.90745800,-3.24074600,-0.60641000
H 5.75727600,0.08129600,-3.70932700
C 6.12753600,-2.51108200,-3.03048300
C 5.61950900,-4.16840500,-1.33925000
H 4.44140100,-3.54613800,0.33230300
C 6.23921900,-3.80397500,-2.56597400
H 6.60149000,-2.21291800,-3.97084100
H 5.71208400,-5.19386800,-0.96995300
H 6.80537100,-4.54836700,-3.13306500
C 2.03025200,-1.69921800,1.13113500
C 3.38378300,-1.36075600,0.96975400
C 1.59497200,-2.26597900,2.37187200
C 4.30412600,-1.56740800,2.06754500
C 2.45816800,-2.44991200,3.42614300
H 0.55877500,-2.58437100,2.48555900
C 3.82840600,-2.09445300,3.31182000
C 5.70393300,-1.28724200,1.96036400
H 2.09584500,-2.88934700,4.36064600
C 4.73270300,-2.29019600,4.39830300
C 6.55814100,-1.49200100,3.02409300
H 6.10745400,-0.91635100,1.01696000
C 6.07086000,-1.99331000,4.26220600
H 4.34191500,-2.68988100,5.33914200
H 7.62388200,-1.27299800,2.91140200
H 6.76023000,-2.15032000,5.09651200
P 0.71855600,-1.24754900,-0.07423500
P 2.74856800,1.63144900,-0.10657400
H -0.88193900,3.29693400,1.10169500
H -2.76136200,3.19428300,1.62375700
H -1.45714200,3.30966600,2.96427000
O 0.12681100,3.15487500,0.32413500
H -0.00161900,-0.00964800,2.50195500
H 0.54136100,1.70072800,2.78710000
H 0.34838000,3.75401300,-0.39936700

Int12

Opt @ BMK/def2SVP in PhCl
SCF Done: E(BMK) = -3867.113008a.u.
Zero-point correction = 1.034844Hartree/Particle
Sum of electronic and thermal Free Energies = -3862.644858a.u.

N 5.13151000,0.95252500,0.04591700
C 5.88328900,2.08554400,-0.48788300
H 6.81168900,2.17101000,0.10250400
H 6.17706000,1.88009100,-1.53447400
C 5.15796000,3.41660200,-0.39986700
C 3.94296600,0.49124200,-0.64605100
H 3.90895600,-0.61016200,-0.61292500
H 4.00266000,0.79795700,-1.70410200

C 2.63271300,0.99474100,-0.03372500
C 1.62450900,1.35266400,-0.81930000
C 1.00875000,1.94466700,-1.90129200
C 1.38372300,3.34821800,-2.31676700
C -0.28938900,1.38794500,-2.30486700
C 5.05004500,4.19679100,-1.48587300
S 5.84000200,0.00791500, 1.20573800
O 6.29530400,0.88899500,2.26221600
O 4.89698100,-1.06503100,1.46848300
C 7.30401100,-0.71086200,0.44379600
C 7.15155900,-1.77921100,-0.45004500
C 8.56821100,-0.20102200,0.75526400
C 8.28700500,-2.32413600,-1.05296700
H 6.15548500,-2.18349500,-0.65486200
C 9.69450600,-0.76175100,0.14237200
H 8.66269900,0.61262800,1.47952100
C 9.57440600,-1.82637100,-0.76799400
H 8.17746500,-3.15932500,-1.75227000
H 10.68662000,-0.36664700,0.38269100
C 10.79843600,-2.45023500,-1.39873800
H 10.58364800,-2.81604300,-2.41524100
H 11.63110900,-1.73213500,-1.45484400
H 11.14189100,-3.31308800,-0.80050400
H 4.60117400,5.19392500,-1.42767900
H 5.42874700,3.87455300,-2.46211100
C 4.65023200,3.81757900,0.96520100
H 3.68728700,3.32374000,1.18147000
H 5.35637300,3.50727200,1.75444400
H 4.48908600,4.90468500,1.02670800
C 2.46612300,0.87431400,1.46389100
H 2.32570000,-0.18643300,1.74389600
H 3.37282100,1.21808500,1.98552300
H 1.61030700,1.47054500,1.81978800
Rh -0.49821700,1.43939300,-0.25619800
C 1.04633600,-1.86078100,-0.77529200
C 1.58572500,-1.63387900,-2.06418300
C 1.77161300,-2.70282300,0.09182000
C 2.73713400,-2.29692300,-2.49656100
H 1.11509900,-0.93038800,-2.75167200
C 2.94243200,-3.34846900,-0.33326800
H 1.43870000,-2.86830200,1.11687200
C 3.41353600,-3.17270800,-1.63581100
H 3.11058700,-2.11964400,-3.50934000
H 3.48032700,-3.99076600,0.36891600
H 4.30771400,-3.70166500,-1.97841900
C -0.75155700,-1.70708900,1.44149800
C -1.02941500,-3.07766000,1.61607800
C -0.62193700,-0.88979000,2.57750700
C -1.17747900,-3.61319600,2.89793200
H -1.13541100,-3.72899600,0.74268700
C -0.77187200,-1.42467800,3.86326900
H -0.39486500,0.17511300,2.46150800
C -1.04939100,-2.78703400,4.02361300
H -1.39820600,-4.67750000,3.01901000
H -0.66662000,-0.77696000,4.73788500
H -1.16340600,-3.20767800,5.02683900

C -3.10512000,2.85811900,1.49758200
C -4.19350800,3.74799600,1.42875700
C -2.18173500,2.99250800,2.55577800
C -4.35635900,4.74278200,2.40229700
H -4.91679200,3.66798700,0.61271800
C -2.34801000,3.98235800,3.52812300
H -1.31954100,2.32077000,2.62219000
C -3.43831700,4.86033200,3.45226300
H -5.20496900,5.42949700,2.33519800
H -1.62118300,4.07483100,4.33988400
H -3.56655600,5.64046200,4.20819100
C -3.88262300,2.03742500,-1.12290200
C -3.37402200,3.05337100,-1.95976800
C -5.17640600,1.54039900,-1.34873400
C -4.14850600,3.54702800,-3.01361300
H -2.38006900,3.46998300,-1.76385300
C -5.94401800,2.03080600,-2.41331000
H -5.59105100,0.77513200,-0.69008800
C -5.43120200,3.03017700,-3.24762700
H -3.75052400,4.33977700,-3.65358100
H -6.94716500,1.63063000,-2.58530000
H -6.03270600,3.41345600,-4.07698700
C -3.65625700,0.06768500,1.08384600
C -3.65266000,-1.21070300,0.51308600
C -4.17828200,0.24457400,2.40687900
C -4.14025900,-2.33592700,1.27857900
C -4.63900400,-0.81847000,3.14535700
H -4.21091700,1.24098800,2.84665400
C -4.61311300,-2.13619600,2.61501800
C -4.13834200,-3.67184400,0.76550900
H -5.02857300,-0.65574500,4.15495000
C -5.05084800,-3.25074000,3.39027000
C -4.56674600,-4.73250700,1.53730400
H -3.78485900,-3.86212800,-0.24924300
C -5.02752100,-4.52515400,2.86610800
H -5.40862700,-3.07213500,4.40900800
H -4.55381600,-5.74396500,1.12101400
H -5.36702300,-5.37542200,3.46449100
C -1.85972900,-1.62280900,-1.27397600
C -3.20742500,-1.50605500,-0.89688100
C -1.55086100,-2.09198700,-2.59071400
C -4.24983700,-1.81983900,-1.85008700
C -2.53270200,-2.37656200,-3.51083400
H -0.51511200,-2.26237000,-2.87755600
C -3.90495300,-2.23130400,-3.17755400
C -5.64097800,-1.76263300,-1.51617900
H -2.25974900,-2.73742700,-4.50734400
C -4.93038300,-2.52963500,-4.12422200
C -6.61295100,-2.06257400,-2.44810700
H -5.94149500,-1.48731100,-0.50379100
C -6.25909600,-2.44449900,-3.77148100
H -4.63852900,-2.83677300,-5.13326300
H -7.66799100,-2.01361500,-2.16348000
H -7.04110700,-2.67946600,-4.49895600
P -0.48507600,-1.00662800,-0.21132800
P -2.86847000,1.51460000,0.28525400

H 0.70111100,4.05748500,-1.81891300
H 2.41634900,3.57797700,-2.01426400
H 1.28117000,3.44151300,-3.41055500
O -0.46840400,3.39868700,-0.07113100
H -1.24087200,3.79218800,0.34541100
H -0.37305800,0.36104200,-2.67308000
H -0.92416700,2.07761500,-2.87616900

Ts12

Opt @ BMK/def2SVP in PhCl
SCF Done: E(BMK) = -3867.075179a.u.
Zero-point correction = 1.029367Hartree/Particle
Sum of electronic and thermal Free Energies = -3862.609299a.u.
Imaginary Frequency is -1140.29cm^{-1}

N 5.24706800,0.83220400,0.58708500
C 5.64122200,2.12281700,0.01436400
H 6.30913600,2.60908400,0.74390300
H 6.20302000,1.97316100,-0.92970200
C 4.44780300,3.02809500,-0.23162500
C 4.33791300,0.00575200,-0.20387100
H 4.64778100,-1.05090100,-0.12327100
H 4.43001000,0.30527300,-1.25556500
C 2.88613100,0.08264900,0.27559200
C 1.85235400,0.42783300,-0.53297800
C 1.79481600,0.68392400,-1.95758100
C 2.71041000,0.23386500,-3.05997600
C 0.60164000,1.38463200,-2.13782900
C 4.16358500,3.45667700,-1.47089600
S 6.32379700,0.08089800,1.60518600
O 6.81107000,1.10011300,2.50742700
O 5.65078000,-1.11252300,2.08186200
C 7.70801600,-0.45289100,0.58304100
C 7.71607400,-1.74918400,0.05464500
C 8.75626700,0.44032200,0.32342300
C 8.77889800,-2.14152200,-0.76620500
H 6.91360900,-2.44631100,0.31020800
C 9.80799200,0.03059300,-0.50023200
H 8.75647500,1.43224000,0.78355300
C 9.83678700,-1.26191900,-1.05877300
H 8.79369800,-3.15634600,-1.17642600
H 10.63082600,0.72371800,-0.70322800
C 11.00198800,-1.70226500,-1.91568400
H 11.85408500,-2.00408600,-1.28094600
H 10.73468700,-2.56311300,-2.54769000
H 11.34994800,-0.88495600,-2.56748400
H 3.32593800,4.13661600,-1.65691500
H 4.78135000,3.16913200,-2.32972100
C 3.66242600,3.43945900,0.98768200
H 2.75560900,3.99416300,0.71010000
H 3.35580300,2.55430100,1.56898500
H 4.29175700,4.05882800,1.65224400
C 2.70173600,-0.25401000,1.72933900
H 3.34116500,0.38873700,2.35780600
H 1.65764900,-0.14856500,2.04728400
H 3.04893600,-1.28359800,1.92750900

Rh -0.12365400,0.95772400,-0.24072300
C 0.29323500,-2.45133800,-1.16646900
C 0.58558300,-2.24141500,-2.53159100
C 0.99085400,-3.46579700,-0.48356500
C 1.49592400,-3.05993900,-3.20642900
H 0.09897400,-1.42718300,-3.07659800
C 1.91831900,-4.27264800,-1.15726600
H 0.82632800,-3.63111400,0.58253100
C 2.16351700,-4.08368500,-2.52086300
H 1.69027700,-2.89299800,-4.27006800
H 2.44673100,-5.05604300,-0.60644300
H 2.87657400,-4.72469400,-3.04726700
C -1.03269600,-1.95138100,1.36741100
C -1.44616600,-3.27208100,1.62869500
C -0.78699500,-1.09003700,2.45194500
C -1.58673100,-3.72170800,2.94500900
H -1.66716600,-3.95054900,0.79821900
C -0.93307000,-1.53561700,3.76955800
H -0.50266900,-0.04466800,2.27818300
C -1.32996100,-2.85562900,4.01626500
H -1.90828900,-4.74933200,3.13598500
H -0.74020000,-0.85225900,4.60096400
H -1.44317900,-3.20923800,5.04498000
C -2.50070800,3.46725400,0.59887700
C -3.19631900,4.52720600,-0.01525900
C -1.68325800,3.76066200,1.71578800
C -3.10160400,5.83095300,0.49159600
H -3.81171300,4.34732800,-0.89885800
C -1.59937200,5.05954300,2.22563700
H -1.09515500,2.96857500,2.19088700
C -2.31293800,6.09990500,1.61466300
H -3.65009500,6.63913000,-0.00065400
H -0.96547700,5.26039300,3.09387600
H -2.24328200,7.11821800,2.00773500
C -3.58608500,1.78070800,-1.49371900
C -3.07380900,1.43483400,-2.75650600
C -4.94920000,2.11800600,-1.37406800
C -3.90661700,1.42909900,-3.88178800
H -2.02482200,1.14369000,-2.85044800
C -5.77708000,2.12029700,-2.50012900
H -5.36797800,2.36818500,-0.39369000
C -5.25756600,1.77334400,-3.75499100
H -3.49960000,1.15190700,-4.85822800
H -6.83401300,2.38145300,-2.39667800
H -5.90886200,1.76948900,-4.63370500
C -3.39180700,0.74721700,1.20995300
C -3.75650500,-0.57379900,0.91945000
C -3.58636400,1.25573800,2.53249600
C -4.30645300,-1.41047100,1.95964800
C -4.10026900,0.46510800,3.53474400
H -3.34537500,2.29542300,2.75557600
C -4.46168200,-0.88677100,3.28362200
C -4.71164100,-2.76122700,1.72244000
H -4.24969000,0.87990000,4.53659000
C -4.99608700,-1.71376000,4.31580100
C -5.23573500,-3.53565800,2.73720800

H -4.60480800,-3.18750600,0.72318500
C -5.37826700,-3.01157400,4.05063600
H -5.10705100,-1.29336000,5.32025000
H -5.54425100,-4.56427000,2.52912400
H -5.79670600,-3.63687800,4.84446600
C -2.52320900,-1.60363100,-1.08813800
C -3.70012900,-1.14942800,-0.47412600
C -2.60727200,-2.21430800,-2.37975300
C -4.96515600,-1.27485500,-1.16317300
C -3.80283100,-2.32697700,-3.05034500
H -1.71634700,-2.63079900,-2.84583200
C -5.00986700,-1.85080700,-2.47283800
C -6.19918500,-0.83800000,-0.58581600
H -3.83380800,-2.80570300,-4.03411300
C -6.25483300,-1.96598800,-3.15955400
C -7.39131300,-0.96925300,-1.26799800
H -6.20059000,-0.39067100,0.40940000
C -7.42472700,-1.53685400,-2.57045100
H -6.26321400,-2.41185800,-4.15902300
H -8.32120000,-0.63192200,-0.80109400
H -8.37775700,-1.63612800,-3.09782200
P -0.86918200,-1.32420800,-0.32616400
P -2.50459700,1.75726500,-0.04000300
H 3.50858900,0.99439800,-3.15784000
H 3.18547500,-0.73332300,-2.84203000
H 2.17986300,0.18311300,-4.02379400
H 0.09200600,1.48751000,-3.10463300
O 0.61336000,2.86758700,-0.12342600
H 0.72813900,2.44174300,-1.40236400
H -0.07182800,3.54764100,-0.09010900

Int13
Opt @ BMK/def2SVP in PhCl
SCF Done: E(BMK) = -3867.113008a.u.
Zero-point correction = 1.034844Hartree/Particle
Sum of electronic and thermal Free Energies = -3862.644858a.u.

N -5.18559900,0.70944900,-0.32130300
C -5.22649800,2.00091000,0.34971500
H -6.22738500,2.43815800,0.19555000
H -5.10061300,1.85895100,1.43692200
C -4.23060300,3.03564800,-0.16235500
C -4.25818700,-0.30977200,0.17486600
H -4.57932100,-1.28250800,-0.23108900
H -4.31952800,-0.36714400,1.27346900
C -2.83011200,-0.05452000,-0.28614600
C -1.82075600,0.26940700,0.54660000
C -1.71766800,0.52094500,2.00777000
C -2.60001800,0.90582800,2.95115400
C -0.21166500,0.47830100,2.18660800
C -3.59065000,3.84579800,0.70172700
S -6.43421100,0.27227000,-1.31730200
O -6.95021100,1.50520600,-1.87904300
O -5.93160900,-0.80026400,-2.15390300
C -7.72294600,-0.42429200,-0.27010500
C -7.72262300,-1.79750400,0.00121400

C -8.70847400,0.41822000,0.26124800
C -8.70696700,-2.32262800,0.84487800
H -6.98001100,-2.44690600,-0.47003800
C -9.68316500,-0.12549100,1.10206200
H -8.72693100,1.47734500,-0.00949800
C -9.69483400,-1.49856500,1.41423000
H -8.71800200,-3.39754500,1.05139100
H -10.46155600,0.52667700,1.51125200
C -10.74370100,-2.06657600,2.34327000
H -10.85246400,-3.15390500,2.21030700
H -10.46798200,-1.88253500,3.39700000
H -11.72482300,-1.59547200,2.17205100
H -2.99644100,4.69628400,0.34764100
H -3.70687000,3.73348500,1.78579900
C -4.12960000,3.16895700,-1.65968500
H -3.71048300,2.24665400,-2.09809100
H -5.13623200,3.28011000,-2.09845800
H -3.50483800,4.02725900,-1.95117900
C -2.60139700,-0.26419100,-1.77192600
H -2.15472200,-1.26173000,-1.95410200
H -3.54892800,-0.22629100,-2.33277400
H -1.91730100,0.49380000,-2.19121000
Rh 0.11402100,0.73888200,0.19785800
C -0.34936200,-2.81359000,0.08902100
C -1.23437900,-2.79472000,1.18971600
C -0.44452600,-3.88485600,-0.82214100
C -2.15712300,-3.82627100,1.38713600
H -1.21668000,-1.97122100,1.90445100
C -1.37724600,-4.91206100,-0.62682200
H 0.19713600,-3.92911200,-1.70294400
C -2.23167500,-4.89040100,0.47917000
H -2.82450600,-3.79125300,2.25286500
H -1.43235900,-5.73056400,-1.35007500
H -2.95704300,-5.69495700,0.63106600
C 1.43348200,-1.67604300,-1.84667900
C 2.31381800,-2.71313200,-2.20667200
C 0.93574600,-0.82686400,-2.85194600
C 2.70129500,-2.88380600,-3.53928600
H 2.70636300,-3.38742600,-1.44001600
C 1.31997700,-0.99754900,-4.18730000
H 0.21666900,-0.04141900,-2.59553000
C 2.20724700,-2.02487300,-4.53055500
H 3.39612900,-3.68697500,-3.80177800
H 0.92113300,-0.33346200,-4.95911700
H 2.51026500,-2.16019500,-5.57280200
C 2.22418000,3.16635100,-1.00198000
C 2.82288500,4.40724200,-0.71450100
C 1.38084000,3.06911900,-2.13140200
C 2.58737800,5.51751000,-1.53781400
H 3.47575300,4.51043300,0.15652000
C 1.14956300,4.17660700,-2.95531900
H 0.91889200,2.10739900,-2.38544000
C 1.75314300,5.40663200,-2.65656400
H 3.06103500,6.47458600,-1.30065800
H 0.50042800,4.07940000,-3.83038800
H 1.57210400,6.27570400,-3.29560600

C 3.17396400,2.34737000,1.55805500
C 2.32502200,3.02900000,2.45583000
C 4.54823900,2.26916700,1.84041900
C 2.84486000,3.61011200,3.61651100
H 1.25795300,3.12586700,2.23138800
C 5.06372700,2.84476900,3.00912800
H 5.22176900,1.76591500,1.14367100
C 4.21467500,3.51200500,3.89911200
H 2.17875800,4.14030900,4.30321700
H 6.13444800,2.77319300,3.22005500
H 4.61944400,3.96141500,4.81053400
C 3.72690900,0.70549000,-0.81590700
C 4.05395700,-0.59286200,-0.40600500
C 4.32930600,1.25025500,-1.99513700
C 4.98389900,-1.37174200,-1.19251800
C 5.20890800,0.51577000,-2.75361500
H 4.08736600,2.26874800,-2.30226000
C 5.54760600,-0.81493700,-2.38510900
C 5.34651500,-2.71251700,-0.84744600
H 5.65623300,0.95128700,-3.65241500
C 6.43199300,-1.59666000,-3.18653500
C 6.20014700,-3.44863500,-1.64377900
H 4.93720600,-3.16679300,0.05696200
C 6.75222300,-2.88848600,-2.82832200
H 6.85179000,-1.14851900,-4.09249700
H 6.45875000,-4.47224800,-1.35767900
H 7.43183500,-3.48169000,-3.44676500
C 2.25233900,-1.78386800,1.00077100
C 3.53741800,-1.23099400,0.86241100
C 1.92607700,-2.50408200,2.19497700
C 4.48937400,-1.37019800,1.94492500
C 2.82146300,-2.63602600,3.22935500
H 0.94687000,-2.97442900,2.28936700
C 4.11747700,-2.06236900,3.14300100
C 5.82296700,-0.85442200,1.86814900
H 2.54238800,-3.19518700,4.12769100
C 5.05181200,-2.19421800,4.21354900
C 6.70837200,-0.99955700,2.91619700
H 6.15438600,-0.34570100,0.96194100
C 6.32212500,-1.67259700,4.10740800
H 4.73928000,-2.72505200,5.11806000
H 7.72179100,-0.59779000,2.82665500
H 7.03543400,-1.78048400,4.92930200
P 0.85252900,-1.45231600,-0.14386300
P 2.43938300,1.67839300,0.04076100
H 0.22128900,1.30220800,2.77506600
O -0.60373600,2.79380100,0.43698500
H -1.57833100,2.86670500,0.49469900
H -0.29148700,3.45316600,-0.19796300
H -2.25428300,1.13957300,3.96425000
H -3.67053100,1.00094700,2.75819100
H 0.18760800,-0.47756600,2.55989500

Int14

Opt @ BMK/def2SVP in PhCl

SCF Done: E(BMK) = -3790.672078a.u.

Zero-point correction = 1.006639Hartree/Particle
Sum of electronic and thermal Free Energies = -3786.312971a.u.

N -5.25061600,0.52109600,-0.70181100
C -5.69862000,1.85824600,-1.09621800
H -6.76666600,1.80243200,-1.36579300
H -5.61307500,2.52888800,-0.22256700
C -4.92885800,2.43562000,-2.27476300
C -4.14930500,0.41107300,0.24391600
H -4.21891700,-0.57890900,0.72763900
H -4.26600100,1.17760600,1.02847800
C -2.75798500,0.47400400,-0.38330400
C -1.70769000,0.97474900,0.29221800
C -1.54422500,1.66743000,1.58026800
C -2.46493500,2.66218000,2.24949900
C -0.27853200,1.31175800,1.98051800
C -4.13486100,3.50347900,-2.11252100
S -6.26187100,-0.76694900,-0.93239500
O -6.89877800,-0.57060000,-2.21821300
O -5.48073600,-1.95495900,-0.63792200
C -7.55371900,-0.64404100,0.31856200
C -7.32608600,-1.16466600,1.59896400
C -8.76470100,-0.01846900,-0.00064000
C -8.31729600,-1.02852700,2.57446000
H -6.39218200,-1.68959600,1.81654100
C -9.74526700,0.10830100,0.98875700
H -8.94002900,0.34064300,-1.01835900
C -9.53930400,-0.38993400,2.28802800
H -8.14574300,-1.43665600,3.57576600
H -10.69421900,0.59528800,0.74223300
C -10.62251100,-0.28629700,3.33766400
H -11.29727300,-1.15932300,3.28093100
H -10.19740600,-0.26094400,4.35317600
H -11.23660900,0.61624700,3.19282900
H -3.59319400,3.94537800,-2.95555400
H -4.01486100,3.98253500,-1.13476700
C -5.16453900,1.76669800,-3.60698300
H -5.08025600,0.67131600,-3.51875300
H -6.19739300,1.96024400,-3.94931000
H -4.46806100,2.13510800,-4.37590500
C -2.62340900,-0.23698200,-1.70579900
H -1.56767200,-0.40828800,-1.96593800
H -3.15099700,-1.20731300,-1.67116500
H -3.08731700,0.35569200,-2.51272000
Rh 0.31765200,1.05259600,0.18229700
C -0.70719000,-2.25630800,0.23831700
C -1.49853700,-1.98657000,1.37630100
C -1.11955900,-3.27544100,-0.64119700
C -2.64709300,-2.73629900,1.64000400
H -1.22565900,-1.16814200,2.04949000
C -2.28431400,-4.01026900,-0.38372700
H -0.55664100,-3.48928600,-1.55108700
C -3.05110300,-3.74440300,0.75334400
H -3.24079900,-2.51844600,2.53249800
H -2.59868200,-4.78391900,-1.08940000
H -3.97002400,-4.30618900,0.93922400

C 1.28351300,-1.64013300,-1.77294100
C 1.81891200,-2.91249100,-2.05465400
C 1.14074600,-0.71144400,-2.81999600
C 2.19842000,-3.24563900,-3.35799000
H 1.94914500,-3.64289000,-1.24982600
C 1.52713100,-1.04271300,-4.12390500
H 0.72043000,0.28221000,-2.62318800
C 2.05416000,-2.31147300,-4.39296800
H 2.61690700,-4.23468500,-3.56450600
H 1.41216200,-0.31111200,-4.92825600
H 2.35313100,-2.57421400,-5.41174100
C 2.69059100,3.31272600,-0.74344900
C 3.61937700,4.34886400,-0.53091700
C 1.68966800,3.49069100,-1.72080800
C 3.55181200,5.52588200,-1.28689700
H 4.39689400,4.24123600,0.23070500
C 1.62490300,4.66391800,-2.48000200
H 0.94394900,2.70418400,-1.90034700
C 2.55905300,5.68496200,-2.26216900
H 4.27837300,6.32410000,-1.10966600
H 0.84170200,4.78380300,-3.23375100
H 2.50852700,6.60730500,-2.84801600
C 3.60360000,2.17428900,1.71415300
C 2.90227800,2.81723600,2.75380500
C 4.98738500,1.96473300,1.85056500
C 3.57130100,3.23261700,3.90956000
H 1.83084900,3.01269500,2.64944000
C 5.65374800,2.37556100,3.01171100
H 5.54918200,1.48576000,1.04506200
C 4.94802600,3.00667400,4.04272500
H 3.01726800,3.73459800,4.70791000
H 6.72953600,2.20361200,3.10768600
H 5.47063600,3.32709600,4.94862500
C 3.77336200,0.62858800,-0.77466500
C 3.93433200,-0.71543300,-0.41277400
C 4.34526500,1.10639500,-1.99604100
C 4.67915800,-1.60099200,-1.27548700
C 5.03731600,0.26622300,-2.83672100
H 4.22916600,2.15551100,-2.27263700
C 5.21414800,-1.10562900,-2.50856900
C 4.88953500,-2.98070500,-0.96094300
H 5.46282500,0.65071600,-3.76885900
C 5.91666300,-1.98828100,-3.38222100
C 5.57701000,-3.81065400,-1.82264700
H 4.49828600,-3.38666300,-0.02616900
C 6.09541100,-3.31403700,-3.04987900
H 6.31671600,-1.58924800,-4.31954900
H 5.72748600,-4.86124300,-1.55762000
H 6.64019500,-3.98329800,-3.72189000
C 2.06171100,-1.65239000,1.06992200
C 3.40599100,-1.29350400,0.87803600
C 1.66689700,-2.28466300,2.29167300
C 4.36848200,-1.56938900,1.92033000
C 2.57270200,-2.53275200,3.29566700
H 0.63113300,-2.59535000,2.42982900
C 3.94069000,-2.17928200,3.14426000

C 5.76183400,-1.27639800,1.77444900
H 2.24670200,-3.02245800,4.21847500
C 4.88963700,-2.44968400,4.17497600
C 6.65916700,-1.55572100,2.78423700
H 6.12371000,-0.83696300,0.84345700
C 6.22197900,-2.14426200,4.00257100
H 4.53835400,-2.91334300,5.10194100
H 7.71980500,-1.32809800,2.64407600
H 6.94538500,-2.36014300,4.79386000
P 0.73239000,-1.19844000,-0.10758600
P 2.70355900,1.75016900,0.19770600
H -2.93802900,3.31151600,1.49541300
H -3.26944100,2.13234400,2.78842700
H -1.92326800,3.28925200,2.97379800
H 0.26108900,1.41247800,2.92872900

Int15
Opt @ BMK/def2SVP in PhCl
SCF Done: E(BMK) = -3867.181337a.u.
Zero-point correction = 1.039196Hartree/Particle
Sum of electronic and thermal Free Energies = -3862.719919a.u.

N -4.36974800,0.10165000,-0.43341300
C -4.43765100,1.44998500,-0.97712900
H -4.95622300,1.41688000,-1.94553300
H -4.99366600,2.12523200,-0.29391700
C -2.99083100,1.93012300,-1.15502700
C -3.83088800,-0.01455700,0.90521800
H -3.48476100,-1.04805200,1.07638000
H -4.61341600,0.19238100,1.67246700
C -2.70293200,0.96331600,1.19923300
C -2.30020600,1.84227400,0.21899400
C -1.27560600,2.90014400,0.29428700
C -1.39967500,3.75701500,-0.96471800
C -0.42341100,3.11199100,1.38525600
C -2.83169900,3.43981300,-1.45427300
S -5.41469100,-1.05748500,-0.98475400
O -5.55835500,-0.82769500,-2.40542100
O -4.93132000,-2.31520000,-0.45130100
C -6.99912200,-0.70857900,-0.20498400
C -7.28255200,-1.25578800,1.05265900
C -7.91972800,0.12085400,-0.85797400
C -8.49857500,-0.94490200,1.66945700
H -6.56769200,-1.93545400,1.52430700
C -9.13014700,0.41896100,-0.22555800
H -7.69604900,0.50404700,-1.85731100
C -9.43893300,-0.10513400,1.04412100
H -8.72789100,-1.37299600,2.65057100
H -9.85572900,1.06251200,-0.73343400
C -10.76868000,0.19313000,1.69859700
H -10.70737500,0.10217000,2.79416600
H -11.11850600,1.20775500,1.45094700
H -11.53778100,-0.51763300,1.34701900
C -2.31387300,1.09446800,-2.25752400
H -2.26587200,0.03241300,-1.97300800
H -2.90758100,1.16650200,-3.18486000

H -1.29907800,1.45643600,-2.47826300
C -2.39677700,1.05133400,2.68821000
H -2.72417200,2.02852700,3.08518000
H -2.94495500,0.26486100,3.22809300
H -1.33084500,0.92918300,2.92743700
Rh -0.03488000,1.14155900,0.47814900
C -0.90135900,-2.20495800,0.99057300
C -1.15581200,-2.00453900,2.36625400
C -1.73168100,-3.08861800,0.27746900
C -2.16909900,-2.71159900,3.01849500
H -0.54363600,-1.29991100,2.93728400
C -2.76133400,-3.78260100,0.92972300
H -1.59574600,-3.23108200,-0.79649900
C -2.97156400,-3.61113300,2.30015400
H -2.33360700,-2.56108800,4.08984500
H -3.41371300,-4.43873200,0.34947600
H -3.76747700,-4.16388500,2.80725900
C 0.55774300,-1.85894500,-1.49698300
C 0.80769200,-3.21825600,-1.77107100
C 0.46593400,-0.94868700,-2.56213700
C 0.94353400,-3.65391300,-3.09263600
H 0.90111900,-3.93718000,-0.95023600
C 0.61017400,-1.38497300,-3.88394300
H 0.29821000,0.11001200,-2.35627000
C 0.84679600,-2.73836500,-4.15107700
H 1.13339100,-4.71109300,-3.29892800
H 0.52975800,-0.65812600,-4.69736800
H 0.95610000,-3.08417800,-5.18304200
C 2.52241100,3.42588400,-0.03917900
C 3.18121300,4.28645600,0.86092600
C 1.96107200,3.98241100,-1.20987200
C 3.29115600,5.65763700,0.58936000
H 3.60536000,3.90072600,1.78950800
C 2.08317600,5.34908000,-1.48159100
H 1.43429200,3.34581400,-1.92756800
C 2.74790000,6.19345600,-0.58211000
H 3.80603900,6.30628000,1.30393700
H 1.65025500,5.75740500,-2.39968700
H 2.83731200,7.26312700,-0.79264000
C 3.16279100,1.37498800,1.87173100
C 2.49270300,1.00899500,3.05229400
C 4.56328600,1.52735900,1.90023200
C 3.20677300,0.79413600,4.23767600
H 1.40589700,0.88032400,3.03461500
C 5.27483200,1.31972800,3.08504500
H 5.10245500,1.80249800,0.98773900
C 4.59791500,0.94928600,4.25515700
H 2.67490700,0.50416600,5.14817100
H 6.36213700,1.43666200,3.09296500
H 5.15667000,0.78180000,5.18043800
C 3.14664200,0.70793200,-0.95085700
C 3.39166900,-0.66296300,-0.80312300
C 3.51411400,1.34754300,-2.17901500
C 3.93061300,-1.42355100,-1.90809900
C 4.04344500,0.63812900,-3.23398800
H 3.40865200,2.42754200,-2.27633000

C 4.24384000,-0.76735100,-3.14080700
C 4.17546800,-2.82908900,-1.81868100
H 4.34376100,1.15872600,-4.14978000
C 4.77960200,-1.51507800,-4.22978200
C 4.69865400,-3.52947700,-2.88658800
H 3.94113400,-3.35676600,-0.89229700
C 5.00520300,-2.86989800,-4.10704800
H 5.01664100,-0.99178500,-5.16143000
H 4.87811800,-4.60432700,-2.79313000
H 5.42306900,-3.43761900,-4.94329900
C 1.98209600,-1.80760000,1.00181900
C 3.22253100,-1.39381800,0.50416200
C 1.94015800,-2.59708500,2.19366600
C 4.43380700,-1.71906400,1.22063700
C 3.08379400,-2.90363300,2.89407400
H 0.98947600,-2.98690200,2.55449200
C 4.35555800,-2.45651400,2.44505800
C 5.72931700,-1.31863000,0.76491700
H 3.02216400,-3.51084300,3.80265000
C 5.54514300,-2.75367900,3.17436200
C 6.86476400,-1.62491500,1.48673800
H 5.82503200,-0.75629100,-0.16543100
C 6.77683800,-2.34718800,2.70772900
H 5.46031800,-3.31915700,4.10759800
H 7.84371300,-1.30884800,1.11476000
H 7.68587700,-2.58408700,3.26789200
P 0.42652000,-1.23516600,0.19973600
P 2.23194700,1.65702000,0.33520600
H -3.56464600,4.02228900,-0.86758100
H -2.98170500,3.67458500,-2.52029100
O 0.72378700,2.01339000,-3.69309900
H 1.55493200,1.52017500,-3.68225400
H 0.70795100,2.46663900,-4.54253400
H -0.68672000,2.84051000,2.40838900
H 0.31852800,3.91151700,1.29454400
H -0.66588000,3.45207000,-1.72640800
H -1.23489600,4.82246900,-0.74215100

Ts13

Opt @ BMK/def2SVP in PhCl
SCF Done: E(BMK) = -3867.133982a.u.
Zero-point correction = 1.029686Hartree/Particle
Sum of electronic and thermal Free Energies = -3862.677738a.u.
Imaginary Frequency is -1279.72cm^{-1}

N 5.99859800,0.63192700,0.28046000
C 5.16981200,0.98113900,1.42415500
H 5.16785300,0.14210200,2.13288700
H 5.60720700,1.86443100,1.92628700
C 3.74419000,1.30020900,0.94570000
C 6.12185000,1.62323300,-0.76515400
H 6.30651700,1.11127700,-1.72759600
H 6.99673400,2.28552800,-0.59010000
C 4.90850000,2.51419600,-0.95395200
C 3.81158900,2.35916800,-0.16157700
C 2.59862100,3.17623300,-0.10313200

C 1.88560300,2.85372100,1.18769600
C 2.06901800,3.99151400,-1.09687300
C 2.87599800,2.01002200,2.01304600
S 6.39551200,-0.94326000,-0.05519500
O 5.82626800,-1.73635200,1.01467900
O 6.06280500,-1.19549300,-1.44708100
C 8.18678000,-1.01105000,0.07964800
C 8.96730400,-1.02934000,-1.07922000
C 8.77191900,-1.04621100,1.35308300
C 10.36224600,-1.06271800,-0.95517900
H 8.48539000,-1.03565200,-2.06042400
C 10.16267700,-1.07976800,1.45546500
H 8.14284200,-1.06479500,2.24735200
C 10.98016800,-1.08742100,0.30604100
H 10.97983800,-1.07748500,-1.85868400
H 10.62760500,-1.10832200,2.44636400
C 12.48391800,-1.16695000,0.44045400
H 12.87943800,-0.29253100,0.98374400
H 12.77184900,-2.06508800,1.01466200
H 12.97681200,-1.22005700,-0.54148600
C 3.04564500,0.03135500,0.40993600
H 3.60107000,-0.39487600,-0.44111300
H 2.97860300,-0.74000300,1.19504600
H 2.02469300,0.26345700,0.05952600
C 5.10522200,3.55467700,-2.02913100
H 4.75849700,4.55103300,-1.71291600
H 6.17119400,3.63321600,-2.29653300
H 4.56010700,3.27725600,-2.94825500
Rh -1.19905700,1.50998200,-0.92493200
C -3.03649700,3.07222500,1.23012200
C -1.85971300,3.31355900,1.97674400
C -4.00475400,4.09103600,1.15104200
C -1.67760600,4.52324700,2.65445800
H -1.08618900,2.53973900,2.03232100
C -3.81330300,5.30695800,1.82149900
H -4.91461300,3.93804800,0.56526100
C -2.65640300,5.52407500,2.57807000
H -0.77137600,4.68696500,3.24496500
H -4.57710000,6.08682300,1.75156800
H -2.51462700,6.47197600,3.10526500
C -4.69974700,1.62846500,-0.64592100
C -5.95700400,1.53063800,-0.01833900
C -4.63831300,1.83118700,-2.03663300
C -7.13059400,1.64050200,-0.76983000
H -6.01749400,1.35630600,1.06118100
C -5.81550800,1.93685200,-2.78815500
H -3.66294400,1.89051500,-2.53218200
C -7.06079900,1.84081300,-2.15610000
H -8.10268600,1.55946800,-0.27527600
H -5.75833700,2.08976900,-3.86960100
H -7.98051100,1.92064600,-2.74289000
C -0.11138500,-1.62633800,-2.15793900
C -0.38344700,-2.94073600,-2.59613100
C 1.06800400,-0.99849300,-2.60302500
C 0.50158500,-3.60587800,-3.44950800
H -1.30151700,-3.44742900,-2.28613500

C 1.95747700,-1.67191100,-3.45185100
H 1.29009900,0.02349400,-2.28933900
C 1.67844100,-2.97502400,-3.87521900
H 0.27132300,-4.62213200,-3.78231900
H 2.87540400,-1.17521300,-3.77842100
H 2.37415900,-3.49935100,-4.53657000
C -0.65365500,-1.57380400,0.62744000
C -0.24037000,-0.78324400,1.71560200
C -0.62022900,-2.97480400,0.76214200
C 0.17807700,-1.37414300,2.91322000
H -0.25407600,0.30610600,1.61511600
C -0.19621200,-3.56678500,1.95585200
H -0.92774700,-3.61712900,-0.06664500
C 0.20174100,-2.76867600,3.03576400
H 0.48384200,-0.74423100,3.75384000
H -0.17837000,-4.65673000,2.04229600
H 0.53282000,-3.23365600,3.96872800
C -2.86829700,-1.33024800,-1.32370800
C -3.88637300,-1.41731300,-0.37002300
C -3.18997800,-1.46752400,-2.71432300
C -5.23817200,-1.71452800,-0.78741200
C -4.47331600,-1.72948600,-3.12726800
H -2.39831100,-1.35833400,-3.45965600
C -5.52808800,-1.87411100,-2.18068100
C -6.31744300,-1.84357100,0.14037300
H -4.69824000,-1.83042800,-4.19364200
C -6.86014600,-2.16029400,-2.59880900
C -7.59763100,-2.12844500,-0.29223800
H -6.12808700,-1.71922100,1.20799900
C -7.87664500,-2.29008000,-1.67532700
H -7.05972800,-2.27993100,-3.66828400
H -8.40511300,-2.23097600,0.43848300
H -8.89509900,-2.51667700,-2.00341800
C -3.45616700,0.18495600,1.52952800
C -3.64951600,-1.13005000,1.08934000
C -3.33272700,0.45426400,2.92629500
C -3.67356900,-2.20737700,2.04125800
C -3.35267700,-0.56795000,3.84884800
H -3.23023800,1.48615800,3.26879500
C -3.50215200,-1.92073200,3.43456700
C -3.84420900,-3.56997300,1.64509900
H -3.26013500,-0.34651800,4.91678300
C -3.49248300,-2.99246000,4.37641600
C -3.83421600,-4.58529100,2.57930600
H -3.98331500,-3.80568500,0.58731700
C -3.65345900,-4.29700000,3.96026900
H -3.35839800,-2.75851500,5.43719200
H -3.96785900,-5.62188200,2.25665300
H -3.64823500,-5.11242700,4.68923600
P -3.15724100,1.50637300,0.29997100
P -1.16816300,-0.77381500,-0.93641800
H 2.74066500,4.23476600,-1.92368700
H 1.17915100,3.06700500,-1.63163000
H 1.40176000,4.79887700,-0.76425400
H 3.50718500,2.66945400,2.63453600
H 2.36586100,1.29621600,2.68183300

O 0.45140600,2.12041100,-2.05262900
H 1.00135400,2.22286100,0.92545600
H 1.49217200,3.74999500,1.69627900
H 0.40321100,2.04725400,-3.01202000

Int16

Opt @ BMK/def2SVP in PhCl
SCF Done: E(BMK) = -3867.160489a.u.
Zero-point correction = 1.040558Hartree/Particle
Sum of electronic and thermal Free Energies = -3862.691878a.u.

N -4.40912700,-0.63734600,-0.08857400
C -4.53071200,0.10202500,-1.33899200
H -4.97977600,-0.56139200,-2.09371500
H -5.18669600,0.98901900,-1.21194100
C -3.13653400,0.55165900,-1.78901000
C -3.88889200,0.12657800,1.02997900
H -3.54303800,-0.57370700,1.80853800
H -4.68569200,0.74629400,1.50629400
C -2.77541900,1.09370000,0.66633900
C -2.42637900,1.30425300,-0.64281300
C -1.44159400,2.26697300,-1.24098800
C -1.82344500,2.38125800,-2.74338000
C -1.30287000,3.64244100,-0.57620400
C -3.17333800,1.64396300,-2.87925500
S -5.61034200,-1.73983300,0.25911600
O -5.76450900,-2.55434700,-0.92382200
O -5.25708400,-2.30358400,1.54978300
C -7.11728500,-0.78391500,0.48514300
C -7.45720700,-0.32418200,1.76402300
C -7.92536300,-0.50395000,-0.62490800
C -8.61247500,0.44836400,1.92097400
H -6.83838900,-0.59524400,2.62369600
C -9.07532700,0.26979600,-0.44691400
H -7.66686000,-0.91041000,-1.60656600
C -9.43646700,0.75904100,0.82336300
H -8.88647800,0.80613800,2.91857400
H -9.71306500,0.48805300,-1.30952100
C -10.70430500,1.56113400,1.00756300
H -10.64444900,2.21039400,1.89468600
H -10.91246500,2.18924800,0.12705800
H -11.56808100,0.88639600,1.14499700
C -2.31920500,-0.66582800,-2.26182000
H -2.32814700,-1.47386100,-1.51643800
H -2.72745100,-1.06041300,-3.20857100
H -1.26771400,-0.39390300,-2.51235400
C -2.37547400,1.92065900,1.86905200
H -3.16420400,2.66979100,2.06856300
H -2.30817200,1.28501500,2.76833300
H -1.42928400,2.45498700,1.75622400
Rh -0.11409200,0.65797900,-0.92703100
C -0.21701200,-2.92460600,0.00629700
C -1.42131400,-2.59600200,0.66007800
C 0.02808500,-4.27193100,-0.32450100
C -2.35228200,-3.58624400,0.99243500
H -1.63686700,-1.54987100,0.90600400

C -0.90479200,-5.26271400,0.00359400
H 0.94583500,-4.55309200,-0.84596700
C -2.09173800,-4.92295600,0.66233100
H -3.29253800,-3.31316800,1.47843200
H -0.70206100,-6.30369700,-0.26421800
H -2.82489900,-5.69610300,0.90946000
C 1.98474900,-2.34410800,-1.69175200
C 3.09780900,-3.16098500,-1.42006500
C 1.56490100,-2.18935800,-3.02911300
C 3.79350600,-3.78599100,-2.46030900
H 3.42262100,-3.31205100,-0.38800800
C 2.25877300,-2.82131300,-4.06836500
H 0.69089600,-1.57691400,-3.26703700
C 3.37730700,-3.61495600,-3.78681400
H 4.66251100,-4.40944800,-2.23071700
H 1.92118900,-2.69138100,-5.10047400
H 3.92103500,-4.10470200,-4.59982800
C 1.79272000,3.54848300,-0.74307200
C 1.95103300,4.66486900,0.10091100
C 1.59461800,3.77118500,-2.12563300
C 1.95266900,5.96336600,-0.42629000
H 2.04853500,4.54483200,1.18009500
C 1.61199900,5.06815900,-2.64604300
H 1.39619700,2.91675100,-2.77933200
C 1.79576900,6.17015500,-1.80006200
H 2.07405100,6.81531200,0.24877700
H 1.46491000,5.21745300,-3.71964700
H 1.80284000,7.18493200,-2.20850900
C 1.84719500,1.94726400,1.69911700
C 0.88442600,1.32963000,2.51728500
C 2.93515100,2.60744600,2.30740000
C 0.98359100,1.39829400,3.91123400
H 0.07003400,0.75807200,2.06339900
C 3.02677500,2.68549000,3.69973200
H 3.72266700,3.05167500,1.69112000
C 2.04833200,2.08677200,4.50422800
H 0.23281600,0.90450500,4.53448700
H 3.87443000,3.20204000,4.15845400
H 2.12471500,2.14548100,5.59366900
C 3.26090700,1.01365500,-0.67078900
C 3.80347700,-0.11523000,-0.03884700
C 3.89578400,1.52876700,-1.84780900
C 5.00809500,-0.72021700,-0.56672600
C 5.01559200,0.93702300,-2.37886200
H 3.49761000,2.41544700,-2.33662500
C 5.60257200,-0.19926800,-1.75925600
C 5.63980300,-1.84510000,0.05335600
H 5.47465200,1.35021700,-3.28217200
C 6.77147500,-0.81131900,-2.30114400
C 6.77522900,-2.41360200,-0.48619000
H 5.21888400,-2.26264800,0.96936200
C 7.34896200,-1.89742700,-1.68014300
H 7.20366000,-0.39608800,-3.21677300
H 7.23993200,-3.26953600,0.01176700
H 8.24828200,-2.35940400,-2.09724900
C 2.03919800,-1.52104700,1.13599900

C 3.20512100,-0.74520200,1.19086500
C 1.56361600,-2.15503200,2.32793400
C 3.90734400,-0.58975500,2.44223300
C 2.21751500,-2.00525500,3.52909000
H 0.66854100,-2.77772200,2.28704000
C 3.39704200,-1.21747600,3.62382900
C 5.10699500,0.18058600,2.55857800
H 1.83770100,-2.50660700,4.42493000
C 4.08301800,-1.05790400,4.86466800
C 5.75615600,0.30928900,3.76937800
H 5.51890700,0.67447400,1.67649500
C 5.24102900,-0.31345900,4.93874500
H 3.67722900,-1.54869900,5.75487300
H 6.67661200,0.89717800,3.83039600
H 5.76770400,-0.20477100,5.89115800
P 0.98414600,-1.60603300,-0.36519100
P 1.71536700,1.83896500,-0.10407800
H -0.77727100,3.63949400,0.38750300
H -0.76362100,4.33628800,-1.23447300
H -2.31060100,4.06561100,-0.40703800
H -3.99880300,2.34352000,-2.65853400
H -3.34731400,1.22612800,-3.88480600
O 0.86433100,0.86210200,-2.61732000
H -1.04777600,1.93315800,-3.38533600
H -1.91439100,3.43791900,-3.04257900
H 1.63657300,0.28569500,-2.66360500

Ts14
Opt @ BMK/def2SVP in PhCl
SCF Done: E(BMK) = -3867.138561a.u.
Zero-point correction = 1.033690Hartree/Particle
Sum of electronic and thermal Free Energies = -3862.682379a.u.
Imaginary Frequency is -1129.33cm^{-1}

N 5.11457000,0.35291600,-0.38233300
C 4.50003100,0.67755800,0.89388600
H 4.14147100,-0.25347300,1.35599600
H 5.22761000,1.15981700,1.57692000
C 3.32606600,1.63480300,0.61733300
C 5.66960400,1.43846500,-1.16331600
H 5.68592700,1.13898300,-2.22594400
H 6.72616500,1.65832900,-0.88111700
C 4.90803500,2.74068500,-1.03679300
C 3.84470800,2.83051600,-0.19319700
C 3.13359900,4.02432900,0.25497700
C 2.37812300,3.70854800,1.38548900
C 3.15171500,5.37507200,-0.38470000
C 2.73585100,2.30927700,1.88285400
S 5.54490600,-1.20169600,-0.75121700
O 4.61324500,-2.05595000,-0.04325900
O 5.68659100,-1.24606900,-2.19332500
C 7.17597100,-1.45392900,-0.03756000
C 8.31359800,-1.18001400,-0.80654100
C 7.28372800,-1.90813700,1.28331000
C 9.57654300,-1.34297200,-0.22798700
H 8.20676400,-0.86964200,-1.84953300

C 8.55474200,-2.06470500,1.84255100
H 6.38356700,-2.15718000,1.85192300
C 9.71851800,-1.78459700,1.10050200
H 10.47016500,-1.13487500,-0.82500700
H 8.64651100,-2.42368900,2.87270100
C 11.08758000,-1.99680700,1.70506000
H 11.84334400,-1.35686800,1.22394900
H 11.08583800,-1.78460200,2.78581300
H 11.40641900,-3.04625100,1.57349000
C 2.23878900,0.87896800,-0.17984100
H 2.68229000,0.31813100,-1.01692400
H 1.72838600,0.15205200,0.47758100
H 1.52214100,1.59249500,-0.64567200
C 5.50774100,3.87787300,-1.83304300
H 5.96987100,4.63287100,-1.17344500
H 6.29473600,3.50619900,-2.50736300
H 4.75207400,4.38657200,-2.45061600
Rh -0.52674300,1.22768700,-0.01620500
C 0.45630000,-1.30460800,-1.83127100
C 0.59338900,-0.39106600,-2.90217400
C 1.45761500,-2.27445000,-1.64313600
C 1.68684800,-0.46324200,-3.76930500
H -0.15508700,0.39619800,-3.04373900
C 2.56664100,-2.32782600,-2.49948300
H 1.40430700,-2.97229100,-0.80453100
C 2.68358200,-1.42852800,-3.56344500
H 1.76977700,0.24672100,-4.59768300
H 3.35827800,-3.05680000,-2.31036600
H 3.56080800,-1.46947000,-4.21460300
C -0.72305600,-2.29958500,0.62575300
C -0.93415700,-3.65127300,0.29070500
C -0.43229600,-1.95940200,1.95891400
C -0.85060100,-4.64265100,1.27255400
H -1.17359700,-3.92759100,-0.74186700
C -0.35709200,-2.95302500,2.94302500
H -0.29273600,-0.90517700,2.22282900
C -0.56679400,-4.29400100,2.60085800
H -1.01702200,-5.68988900,1.00443900
H -0.14172000,-2.67846100,3.97961800
H -0.51173900,-5.07063500,3.36918200
C -2.88988100,2.90175600,1.72156200
C -3.87139500,3.90344700,1.60427600
C -1.93924400,3.01261800,2.76228900
C -3.91442900,4.97141700,2.51256600
H -4.60547000,3.86404200,0.79668400
C -1.99180900,4.07050700,3.67504900
H -1.15560400,2.25253000,2.85311000
C -2.98255000,5.05542800,3.55177800
H -4.68446100,5.74040800,2.40209700
H -1.25746100,4.12850400,4.48400300
H -3.02277000,5.88708700,4.26130300
C -3.90810300,1.85651700,-0.77044600
C -3.42288200,2.18789100,-2.04878400
C -5.29942200,1.81737400,-0.55362100
C -4.31266600,2.47085600,-3.09240600
H -2.34123200,2.21025400,-2.21933700

C -6.18645400,2.10511700,-1.59534900
H -5.69238300,1.54051800,0.43040500
C -5.69395200,2.42904000,-2.86703800
H -3.92546900,2.72075600,-4.08430200
H -7.26488500,2.06256100,-1.41845300
H -6.38940800,2.64614800,-3.68299800
C -3.40580000,0.03811600,1.44413700
C -3.67811700,-1.15868700,0.76835100
C -3.50706600,0.07473500,2.87102000
C -4.02866000,-2.34253200,1.51727600
C -3.84609400,-1.04450800,3.59546700
H -3.32822500,1.00906900,3.40443000
C -4.10565800,-2.28262300,2.94585300
C -4.30162700,-3.59554100,0.88448400
H -3.92735200,-0.98767300,4.68559300
C -4.45077900,-3.45086500,3.68776200
C -4.63860300,-4.70917500,1.62649900
H -4.23946200,-3.67301300,-0.20254700
C -4.71596500,-4.64074100,3.04396000
H -4.50698500,-3.38166300,4.77868600
H -4.84643400,-5.65529000,1.11845600
H -4.98683800,-5.53107800,3.61863900
C -2.45680600,-1.30671500,-1.45929500
C -3.65326900,-1.26518600,-0.73490600
C -2.49780400,-1.47255100,-2.87823600
C -4.91505300,-1.36723500,-1.42621800
C -3.69471500,-1.54953200,-3.55300800
H -1.56677300,-1.55062000,-3.44111100
C -4.93112900,-1.48451500,-2.85330400
C -6.16996200,-1.34250800,-0.74100400
H -3.70441700,-1.67470900,-4.64033900
C -6.17779000,-1.55508400,-3.54375600
C -7.36072800,-1.42103800,-1.43374700
H -6.18660600,-1.25781800,0.34730200
C -7.36955400,-1.52407900,-2.85157500
H -6.16840500,-1.64264700,-4.63474600
H -8.30790400,-1.40432700,-0.88669400
H -8.32098900,-1.58545100,-3.38761500
P -0.86236800,-0.97924200,-0.61235700
P -2.72209400,1.49536200,0.55740900
H 2.87567200,5.30940200,-1.45027900
H 2.46012500,6.06274100,0.12329900
H 4.16763100,5.80303800,-0.33573400
H 3.49069600,2.38353100,2.68727800
H 1.86829500,1.75877600,2.28283100
O -0.03223300,3.24610900,0.18140900
H 1.23779600,3.55011700,0.70267900
H 2.02268900,4.49930000,2.05986100
H -0.66298800,3.79841900,0.65839300

Int17
Opt @ BMK/def2SVP in PhCl
SCF Done: E(BMK) = -3867.175873a.u.
Zero-point correction = 1.039124Hartree/Particle
Sum of electronic and thermal Free Energies = -3862.718158a.u.

N -5.05018400,0.30978700,0.20284400
C -4.59433200,0.92716000,-1.03231400
H -4.96959800,0.34732700,-1.88747400
H -4.98805500,1.95815700,-1.10534800
C -3.05722000,0.95118900,-1.01145000
C -4.69931500,0.97124400,1.44312100
H -4.70912000,0.22124200,2.25099300
H -5.44912700,1.74737900,1.71615300
C -3.33694500,1.64427900,1.41042000
C -2.59509400,1.65499700,0.27952800
C -1.33719900,2.39876400,-0.03514900
C -1.15303700,2.35542600,-1.43176100
C -0.75523300,3.48735100,0.83845900
C -2.38434900,1.78559700,-2.12662100
S -5.87475600,-1.11614700,0.20205300
O -5.50659200,-1.80770500,-1.01819300
O -5.66447900,-1.71240900,1.50944800
C -7.62060900,-0.70018500,0.07926200
C -8.34643500,-0.43468700,1.24665400
C -8.22827100,-0.64623600,-1.18077000
C -9.69530600,-0.08329000,1.14007000
H -7.86559600,-0.53023600,2.22393300
C -9.57839100,-0.29384100,-1.26618400
H -7.65532700,-0.90438200,-2.07526100
C -10.33060000,-0.00035000,-0.11322800
H -10.27057800,0.11574200,2.04986900
H -10.06187100,-0.26034900,-2.24782300
C -11.78329500,0.40406100,-0.22172900
H -12.33569300,0.16836400,0.70105600
H -11.86957700,1.49180400,-0.39436800
H -12.27822400,-0.10509200,-1.06373400
C -2.52963200,-0.49251300,-1.08386800
H -2.80933400,-1.09122100,-0.20637600
H -2.90114000,-0.99770900,-1.99130800
H -1.41465500,-0.55413200,-1.20167600
C -2.99053200,2.33535600,2.71389700
H -3.23543700,3.41205100,2.68082600
H -3.56928200,1.89957100,3.54483000
H -1.92531300,2.24263600,2.96572200
Rh -0.00540500,0.69998800,-0.52821400
C -0.51432800,-2.20931600,1.06132400
C -1.22259200,-1.46864400,2.03438700
C -1.04058500,-3.44108700,0.63422500
C -2.42349000,-1.94860100,2.56023100
H -0.84575100,-0.49173600,2.35664700
C -2.25270800,-3.91442500,1.15818800
H -0.52323200,-4.02515300,-0.13080300
C -2.95108600,-3.16925600,2.11238200
H -2.96579200,-1.36250900,3.30781000
H -2.65929500,-4.86440700,0.80019200
H -3.91694100,-3.51458700,2.48825000
C 1.42758500,-2.56193300,-1.05947800
C 2.07662000,-3.77606700,-0.76509900
C 1.18737200,-2.21417600,-2.40055400
C 2.48276800,-4.62333300,-1.80103500
H 2.27581600,-4.05439200,0.27525800

C 1.60153300,-3.06077500,-3.43589500
H 0.68678300,-1.27165400,-2.64464700
C 2.25188700,-4.26427900,-3.13701600
H 2.98939500,-5.56384200,-1.56656300
H 1.41070900,-2.77108800,-4.47329500
H 2.57754000,-4.92745700,-3.94373900
C 2.20875400,3.23577500,-1.37312000
C 2.61160500,4.46602800,-0.81779200
C 1.77800800,3.21767300,-2.72014900
C 2.61604100,5.63476400,-1.59275800
H 2.91269900,4.53247500,0.22902400
C 1.79769300,4.38320000,-3.49249000
H 1.41705600,2.28563400,-3.17076400
C 2.21945100,5.59701500,-2.93243000
H 2.93169600,6.57839200,-1.13855600
H 1.46865200,4.34549800,-4.53515500
H 2.22750800,6.50936200,-3.53581100
C 2.73441300,2.11749200,1.24673800
C 1.95243300,1.90217700,2.39520100
C 4.03682500,2.63515600,1.39598000
C 2.45306600,2.21018500,3.66537900
H 0.95695900,1.46158100,2.28733600
C 4.53269500,2.95288200,2.66380400
H 4.67179800,2.78262000,0.51619200
C 3.74141400,2.74158900,3.80074200
H 1.83790200,2.02883100,4.55120400
H 5.54488400,3.35413600,2.76590900
H 4.13276400,2.98524100,4.79259500
C 3.36481700,0.54962800,-1.09785800
C 3.83191800,-0.56956700,-0.39281700
C 3.80925200,0.74890600,-2.44549000
C 4.70000500,-1.52455100,-1.04645400
C 4.63391100,-0.15293700,-3.07904200
H 3.51942400,1.65117900,-2.98200100
C 5.08692600,-1.32258400,-2.40935800
C 5.19453400,-2.68927300,-0.37981500
H 4.97243200,0.03877500,-4.10282500
C 5.93416000,-2.26793500,-3.05860800
C 6.01864300,-3.58580500,-1.02895600
H 4.91309300,-2.87510700,0.65795700
C 6.39434900,-3.37826100,-2.38337200
H 6.21894300,-2.09007300,-4.10032200
H 6.38591400,-4.46693700,-0.49499000
H 7.04977500,-4.09670400,-2.88379500
C 2.25815000,-1.34450000,1.44335400
C 3.50313100,-0.84272100,1.05328100
C 2.02096900,-1.68098700,2.81109000
C 4.53601900,-0.64846700,2.03898000
C 2.99605500,-1.49402900,3.76456500
H 1.05672100,-2.10208200,3.10240700
C 4.26778200,-0.96289600,3.41126000
C 5.83200100,-0.14568600,1.70439900
H 2.80264100,-1.76236400,4.80794100
C 5.28161800,-0.75599500,4.39363500
C 6.79573600,0.03630400,2.67482000
H 6.06248000,0.09925800,0.66554200

C 6.52042000,-0.26776900,4.03603700
H 5.05913700,-1.00164900,5.43676500
H 7.78180900,0.41822700,2.39493000
H 7.29469000,-0.11961200,4.79423800
P 0.89613100,-1.41327600,0.23354400
P 2.08807600,1.69009700,-0.39796400
H -0.40426300,3.13192000,1.81545300
H 0.08369700,3.98366600,0.32721400
H -1.53196900,4.25147400,1.01959700
H -0.51510200,3.07594700,-1.94789700
H -3.02772800,2.63140000,-2.43785700
H -2.15983900,1.18938800,-3.02628900
O 1.12309300,0.26876100,-4.30681600
H 1.19982000,0.48962300,-5.24077700
H 2.00655400,-0.01898100,-4.04129700

2

Opt @ BMK/def2SVP in PhCl
SCF Done: E(BMK) = -1302.786141a.u.
Zero-point correction = 0.380214Hartree/Particle
Sum of electronic and thermal Free Energies = -1301.226471a.u.

C 0.59438700,0.63758800,1.24179200
H 0.49378100,0.45090900,2.32416600
H -0.12940000,1.44642500,0.98469700
C 1.99072900,1.15257700,0.94669700
C 2.78568300,0.53359700,0.04714500
C 4.13842900,0.91364900,-0.43309400
S -1.09037400,-1.40121500,0.95630100
O -1.05603400,-2.67365700,0.26691900
O -1.19569700,-1.30792700,2.39738500
C -2.45797600,-0.45032600,0.25964300
C -3.05873100,0.55622600,1.02485900
C -2.88338800,-0.71120800,-1.04903200
C -4.08407700,1.32173300,0.45897800
H -2.73588700,0.71699500,2.05709700
C -3.90907900,0.06381700,-1.59836700
H -2.42640700,-1.52705800,-1.61587500
C -4.52342100,1.09060100,-0.85702000
H -4.55772600,2.10869800,1.05489300
H -4.24606500,-0.13835000,-2.62039000
C -5.65734200,1.89755100,-1.44990000
H -5.52692100,2.03378700,-2.53531200
H -6.62212600,1.38249100,-1.29359300
H -5.73090600,2.89124500,-0.98081500
C 2.35566100,2.40265200,1.71150500
H 2.97020900,3.08255800,1.09980200
H 1.44818400,2.94330900,2.02963700
H 2.92904000,2.16352800,2.62624300
C 2.84719200,-1.98550800,-0.21088100
H 2.62366600,-2.82188000,-0.89580400
H 3.93780500,-1.94558000,-0.05454500
H 2.36923200,-2.19505100,0.75868600
C 5.07303600,1.65352800,0.19047800
C 4.34633000,0.24266900,-1.79375800
N 0.28205500,-0.59325700,0.53398100

C 0.77920500,-0.68434300,-0.83284700
C 2.31304400,-0.65821400,-0.78880600
C 2.96435400,-0.34284100,-2.15719800
H 0.41947900,-1.62507700,-1.27444500
H 0.40393400,0.16559600,-1.44066600
H 3.02767100,-1.22753500,-2.81223300
H 2.36253500,0.42309200,-2.67823100
H 6.03870900,1.84430600,-0.29038700
H 4.93051800,2.05684100,1.19477900
H 5.10008500,-0.55920500,-1.70304800
H 4.72182000,0.95238300,-2.54895300

3

Opt @ BMK/def2SVP in PhCl
SCF Done: E(BMK) = -1302.790578a.u.
Zero-point correction = 0.379513Hartree/Particle
Sum of electronic and thermal Free Energies = -1301.232446a.u.

N -0.27287400,0.73398500,0.44771300
C -0.79275200,0.67393800,-0.91224100
H -0.44211300,1.56433700,-1.45451900
H -0.42894900,-0.23418000,-1.43698100
C -2.32411000,0.65335700,-0.83209200
C -0.62189800,-0.36172700,1.34204900
H -0.56303500,0.00982400,2.37877400
H 0.11597300,-1.19415500,1.26055900
C -1.99900800,-0.95239500,1.08338900
C -2.77051500,-0.50660400,0.06835500
C -4.05384800,-1.00861300,-0.49310800
C -4.19538900,-0.52157000,-1.74546100
C -5.04153300,-1.92576600,0.18018100
C -3.03228900,0.34267100,-2.17731600
S 1.12442500,1.55020000,0.74816800
O 1.12678700,2.71539000,-0.11129000
O 1.23954800,1.65567900,2.18790100
C 2.45598600,0.46792200,0.18588300
C 3.04788600,-0.42592900,1.08566000
C 2.86712300,0.52115800,-1.15213100
C 4.04741500,-1.29168500,0.62766000
H 2.74304200,-0.41718400,2.13570400
C 3.86662400,-0.35124500,-1.59232500
H 2.42389000,1.25887800,-1.82677800
C 4.46648200,-1.27422500,-0.71470900
H 4.51937800,-1.98582000,1.33059600
H 4.19754400,-0.30615000,-2.63512400
C 5.52695800,-2.23252400,-1.21030300
H 6.18506300,-2.56133800,-0.39081900
H 5.06163900,-3.13419100,-1.64747300
H 6.15019100,-1.76977000,-1.99213700
C -2.84641800,1.99315000,-0.27022000
H -2.41385700,2.19527300,0.72108000
H -2.57094800,2.82328600,-0.94369900
H -3.94457800,1.96786100,-0.17795500
C -2.30979600,-2.09045100,2.02793800
H -1.57926000,-2.91076700,1.89997600
H -2.22513400,-1.75159600,3.07650200

H -3.31398900,-2.50459000,1.88677800
H -5.30663900,-1.56222800,1.18746200
H -5.96511000,-1.99329200,-0.41601600
H -4.63922100,-2.94739100,0.29019000
H -2.37095600,-0.21953400,-2.86621300
H -3.34527200,1.25876200,-2.70792900
H -5.04450900,-0.75246600,-2.39679200

RhL+

Opt @ BMK/def2SVP in PhCl
SCF Done: E(BMK) = -2487.945462a.u.
Zero-point correction = 0.628145Hartree/Particle
Sum of electronic and thermal Free Energies = -2485.149345a.u.

Rh -0.04178700,-2.48601000,-0.02666100
C 2.76538000,-1.88354500,1.42274500
C 2.24392300,-2.75628200,2.40648600
C 4.15838300,-1.84313500,1.21887000
C 3.09742500,-3.54870000,3.18126700
H 1.16100800,-2.80342300,2.57521700
C 5.00817400,-2.63966200,1.99622100
H 4.58332200,-1.19789500,0.44618000
C 4.48244500,-3.48949800,2.97721500
H 2.68052800,-4.21490300,3.94191200
H 6.08823800,-2.59797100,1.82897500
H 5.15155000,-4.11136200,3.57896400
C 2.50806800,-0.27875000,-0.98861600
C 3.41903200,0.77886600,-0.79626900
C 2.32585400,-0.80766500,-2.27917500
C 4.13922200,1.29151400,-1.87937300
H 3.55452000,1.21434300,0.19952200
C 3.04308700,-0.28708100,-3.36246900
H 1.60729200,-1.61983800,-2.43277700
C 3.94990100,0.76130300,-3.16310000
H 4.84269600,2.11417800,-1.72346500
H 2.89195300,-0.70011400,-4.36370500
H 4.51023200,1.16839100,-4.00967800
C -2.82441000,-1.75196600,-1.46727400
C -4.21622200,-1.64765400,-1.27870100
C -2.33310000,-2.63414600,-2.45780400
C -5.09355800,-2.39242600,-2.07643500
H -4.61820400,-0.99238500,-0.50202300
C -3.21392400,-3.37539100,-3.25253800
H -1.25173100,-2.72912200,-2.61656500
C -4.59685900,-3.25363100,-3.06273800
H -6.17237200,-2.30154200,-1.92105400
H -2.82010300,-4.05042800,-4.01768300
H -5.28739800,-3.83548700,-3.68010700
C -2.52484000,-0.22798100,0.98540900
C -2.40608900,-0.83646800,2.24819800
C -3.37134500,0.88766100,0.83149800
C -3.12376800,-0.33804400,3.34160700
H -1.73861800,-1.69596100,2.37257000
C -4.09001100,1.38040000,1.92492300
H -3.45791600,1.38231500,-0.14176100
C -3.96566100,0.76945800,3.18038000
H -3.02340300,-0.81414000,4.32099500

H -4.74254700,2.24893300,1.79922700
H -4.52557500,1.15987900,4.03505800
C -0.94101600,0.52257500,-1.34921700
C -0.09552600,1.45025900,-0.72593800
C -1.15397000,0.58247300,-2.76094700
C 0.55418000,2.46265000,-1.51970000
C -0.52649600,1.53568900,-3.52978100
H -1.82836400,-0.13332200,-3.23596800
C 0.34552100,2.49145100,-2.93737200
C 1.42026900,3.44517600,-0.94628200
H -0.70135200,1.57223500,-4.60956700
C 1.00059300,3.48234200,-3.72736500
C 2.03420100,4.39675800,-1.73456400
H 1.59599500,3.44311000,0.13136100
C 1.82649000,4.41761700,-3.14081200
H 0.82811300,3.49039100,-4.80807700
H 2.68719400,5.14339700,-1.27346900
H 2.31886400,5.17903100,-3.75241300
C 0.95986000,0.45735200,1.36625200
C 0.15325700,1.43107900,0.76187800
C 1.17281700,0.47808700,2.77892800
C -0.45515400,2.45314500,1.57600900
C 0.58210600,1.43883700,3.56729800
H 1.81747300,-0.27402400,3.23893600
C -0.24817500,2.44249400,2.99442900
C -1.27600500,3.48572200,1.02395000
H 0.75499200,1.44457900,4.64798100
C -0.86614700,3.43973300,3.80611700
C -1.85382100,4.44239800,1.83320600
H -1.44775600,3.52045300,-0.05356400
C -1.65214600,4.42086200,3.24029300
H -0.69727600,3.41504600,4.88714000
H -2.47288200,5.22652500,1.38776800
H -2.11656800,5.18620100,3.86860300
P 1.57779000,-0.96153400,0.40263800
P -1.60866600,-0.89779100,-0.42123800

H_2O
Opt @ BMK/def2SVP in PhCl
SCF Done: E(BMK) = -76.429054a.u.
Zero-point correction = 0.021866Hartree/Particle
Sum of electronic and thermal Free Energies = -76.316607a.u.

O 3.78715822,0.08917955,0.00000000
H 4.74715822,0.08917955,0.00000000
H 3.46670363,0.99411538,0.00000000

9. References

[1]K.-H. Rui, S. Yang, Y. Wei and M. Shi, Rh(I)-Catalyzed Stereoselective Intramolecular Cycloaddition Reactions of Ene-Vinylidenecyclopropanes for the Construction of Fused 6,5-Bicyclic Skeletons with a Quaternary all-Carbon Stereocenter, *Org. Chem. Front.*, 2019, **6**, 2506-2513.

[2] (a) L. Castle, C. A. Honeybone, S. M. Jickells, M. R. Philo and M. Sharman, Practical Aspects of Testing Food Contact Materials for Migration, *Food Additives and Contaminants*, 1994, **11**, 177–185. (b) C. Decaro, K. Ruegg and A. Deagostini, Coulometric Karl Fischer Titration with a Diaphragm-free Cell: Cell Design and Applications, *Food Chemistry*, 2006, **96**, 431–435. (c) Y. S. Tan, S. Chen, W. M. Hong, J. M. Kan, E. S. H. Kwek, S. Y. Lim, Z. H. Lim, M. E. Tessensohn, Y. Zhang and R. D. Webster, The Role of Low Levels of Water in the Electrochemical Oxidation of α-Tocopherol (Vitamin E) and other Phenols in Acetonitrile, *Phys. Chem. Chem. Phys.*, 2011, **13**, 12745; (d) Q. Wang, X.-Y. Tang and M. Shi, Metal-Free Cross-Coupling of Arylboronic Acids and Derivatives with DAST-Type Reagents for Direct Access to Diverse Aromatic Sulfinamides and Sulfonamides, *Angew. Chem. Int. Ed.*, 2016, **55**, 10811–10815; (e) D. Zhao, B. Zhu, L. Li, X. Liu, L. Wen, Y. Song, H. Shen, M. Li, X. Li and D. Wu, A Review of Methods for Measuring Oil Moisture, *Measurement*, 2023, **217**, 113119-113131.

[3]
